# Role of mRNA Stability during Bacterial Adaptation

**DOI:** 10.1371/journal.pone.0059059

**Published:** 2013-03-13

**Authors:** Clémentine Dressaire, Flora Picard, Emma Redon, Pascal Loubière, Isabelle Queinnec, Laurence Girbal, Muriel Cocaign-Bousquet

**Affiliations:** 1 Université de Toulouse; The Institut National des Sciences Appliquées, UPS, INP, LISBP, Toulouse, France; 2 Institut National de la Recherche Agronomique, UMR792 Ingénierie des Systèmes Biologiques et des Procédés, Toulouse, France; 3 Centre national de la recherche scientifique, UMR5504, Toulouse, France; 4 Université de Toulouse; LAAS-Centre national de la recherche scientifique, Toulouse, France; University of Cambridge, United Kingdom

## Abstract

Bacterial adaptation involves extensive cellular reorganization. In particular, growth rate adjustments are associated with substantial modifications of gene expression and mRNA abundance. In this work we aimed to assess the role of mRNA degradation during such variations. A genome-wide transcriptomic-based method was used to determine mRNA half-lives. The model bacterium *Lactococcus lactis* was used and different growth rates were studied in continuous cultures under isoleucine-limitation and in batch cultures during the adaptation to the isoleucine starvation. During continuous isoleucine-limited growth, the mRNAs of different genes had different half-lives. The stability of most of the transcripts was not constant, and increased as the growth rate decreased. This half-life diversity was analyzed to investigate determinants of mRNA stability. The concentration, length, codon adaptation index and secondary structures of mRNAs were found to contribute to the determination of mRNA stability in these conditions. However, the growth rate was, by far, the most influential determinant. The respective influences of mRNA degradation and transcription on the regulation of intra-cellular transcript concentration were estimated. The role of degradation on mRNA homeostasis was clearly evidenced: for more than 90% of the mRNAs studied during continuous isoleucine-limited growth of *L. lactis*, degradation was antagonistic to transcription. Although both transcription and degradation had, opposite effects, the mRNA changes in response to growth rate were driven by transcription. Interestingly, degradation control increased during the dynamic adaptation of bacteria as the growth rate reduced due to progressive isoleucine starvation in batch cultures. This work shows that mRNA decay differs between gene transcripts and according to the growth rate. It demonstrates that mRNA degradation is an important regulatory process involved in bacterial adaptation. However, its impact on the regulation of mRNA levels is smaller than that of transcription in the conditions studied.

## Introduction

Living microorganisms continuously face unsettled growth conditions as their physico-chemical environment and their nutritional resources often fluctuate. They have consequently developed efficient adaptation mechanisms that sustain survival and growth, even in adverse conditions. In response to environmental stimuli or stress, complex regulatory networks adjust the physiological and metabolic status of the cell. There are multiple levels of regulation to ensure an adequate functional state of the cell, and they involve modulating mRNA concentrations, protein concentrations and/or protein activities. We investigated the regulation of the mRNA pool in the cell, which is the first level of the control of gene expression. The post-genomic tools now available allow quantification of mRNA at a genomic scale (transcriptome) in a wide range of microorganisms and are routinely used to access directly the modulation of gene expression in response to various stresses [1], [2], [3].

**Figure 1:**
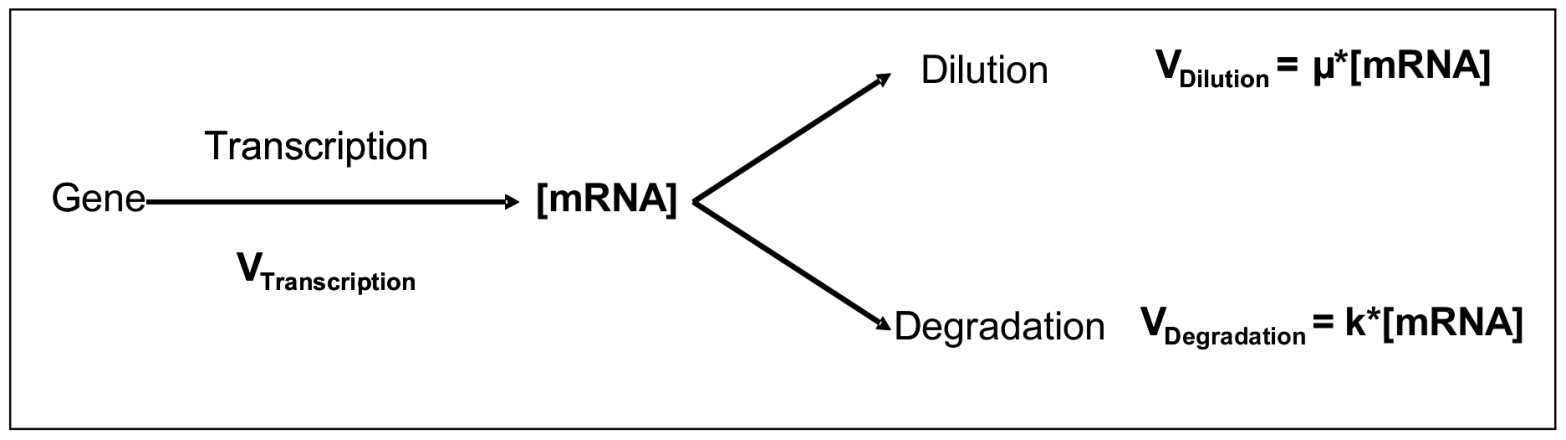
Cellular processes influencing mRNA concentration. Transcription, mRNA decay and dilution due to growth are involved. Dilution and degradation rates can be modeled as µ*[mRNA] and k*[mRNA], respectively, where µ is the growth rate and k the degradation rate constant.

In most such transcriptomic analyses, changes in mRNA levels are attributed to transcriptional regulation. This assumption could however be misleading [4], [5]. Indeed, as illustrated in figure 1, the mRNA concentration in the cell results from the balance between synthesis and elimination of the transcript. Therefore, three processes, namely transcription, mRNA degradation and dilution due to growth, influence mRNA concentration and thereby protein production. mRNA degradation has been widely studied but most studies have addressed the mechanisms involved [6], [7], [8], [9]; there have been relatively few studies of genome-wide mRNA stability [10]. For the model yeast *Saccharomyces cerevisiae*, mRNA half-lives were found to be heterogeneous, ranging from 3 to about 100 min with a mean of 23 min [11]. Shorter mean half-lives, less than 10 min have been reported for the model bacteria *Escherichia coli* [12], [13] and *Bacillus subtilis* [14].

The role of mRNA decay in the regulation of gene expression has been documented at the level of individual genes (see [15], [16] for reviews). It has also been evidenced at the genomic scale: generalized stabilization of *Lactococcus lactis* transcripts has been observed in response to carbon starvation [17], with mean half-lives of 5.8 min during exponential growth and 19.4 min in conditions of starvation. Similarly, mRNA degradation in *S. cerevisiae* is modulated during the response to stress [18]. Growth rate reduction is a characteristic response encountered in all stressing conditions. It affects gene expression in both yeast [19], [20] and in *L. lactis* [21], [22], [23], so the growth rate reduction may affect mRNA stability. We used a genome-wide transcriptomic-based method to test this possibility. *L. lactis* was cultured in chemostat under conditions of isoleucine limitation, and mRNA half-lives were determined at different growth rates. The contribution of mRNA degradation to the regulation of transcript concentration in response to growth modification was evaluated. Dynamic adaptation to isoleucine starvation was also studied in batch culture, as the growth rate changed in response to the increasing stress of falling isoleucine availability.

## Results

### The Effect of Growth Rate on mRNA Half-life Variability

The growth rate alone can influence various cellular processes including transcription [21], translation and protein degradation [22]. This raises the issue of whether mRNA decay is dependent on the growth rate. To study the effects of growth rate on mRNA degradation, we used continuous cultures, as previously described [21], [22] and a transcriptomic-based method to measure mRNA half-lives (cf. Material & Methods for details). Cultures were maintained in a chemostat at steady state at two growth rates (µ): 0.11 and 0.51 h^−1^ (doubling time of 6.30 and 1.36 h respectively). A third growth rate, µ = 0.80 h^−1^ (doubling time = 0.87 h), was studied, but as this growth rate could not be reached in continuous culture without any wash out of the cells from the chemostat, exponentially growing batch cultures were used (during the exponential growth phase, the cells are physiologically stable and can be considered to be at steady-state [24]).

The half-lives of mRNAs were determined by microarrays-based analysis in cultures growing at 0.11, 0.51 and 0.80 h^−1^ (see Material & Methods). 356, 559 and 448 mRNAs were scored as being extremely stable (Table S1). Indeed, their stability could not be precisely quantified because of the short time-course of the experiment. Many of these extremely stable mRNAs were common to the three growth rates: 400 were classified as extremely stable in at least two out of the three studied growth rates. Thus, some mRNAs are always very stable, independent of the growth rate. Most of them correspond to genes in the functional categories “others” and “unknown” as defined by Bolotin *et al* [25].

**Figure 2:**
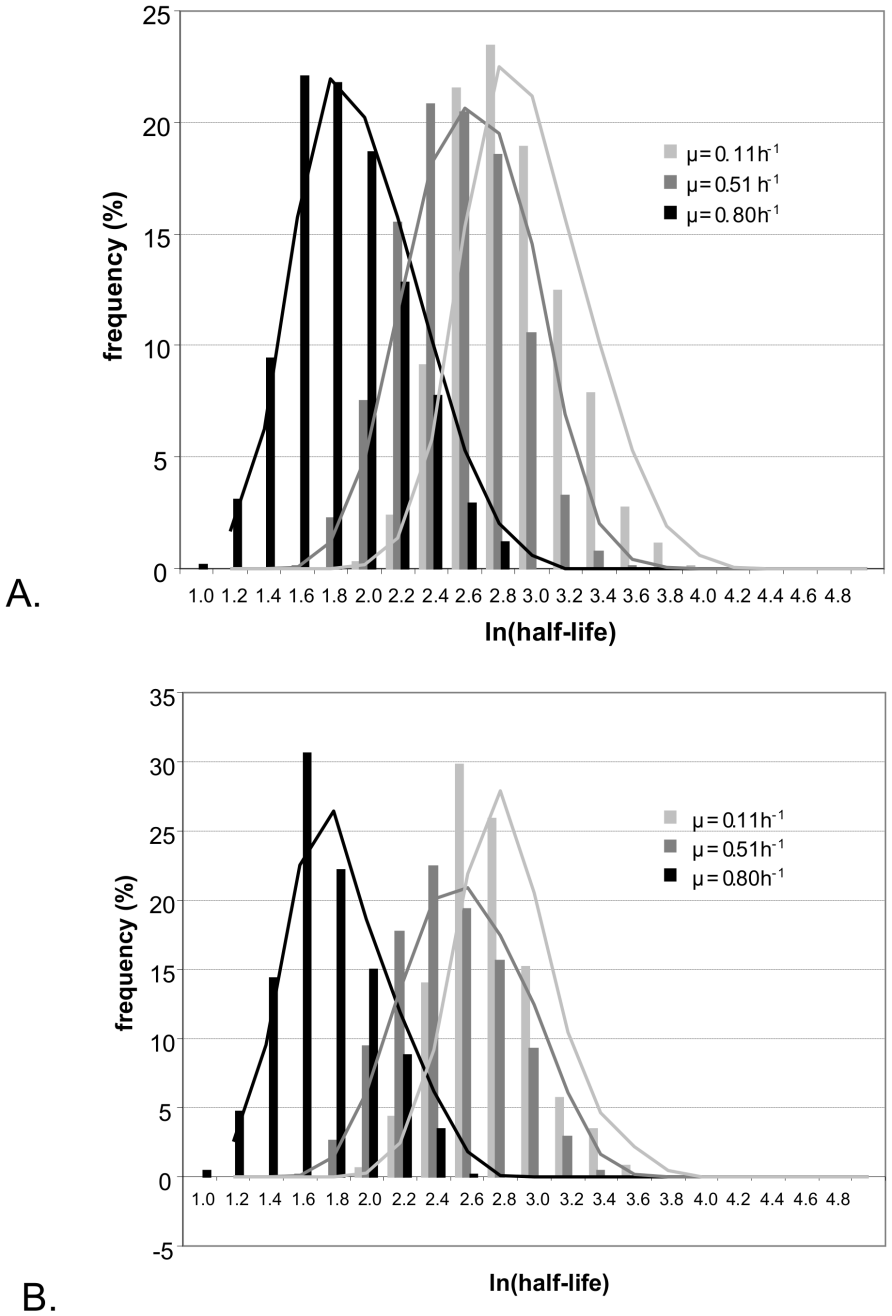
mRNA half-life distribution. A. For transcripts for which stability values at each growth rate were available. B. For the 486 transcripts with half-lives values available for all three growth rates. The darker the histogram, the higher is the growth rate: black for µ = 0.80 h^−1^, dark gray for µ = 0.51 h^−1^ and light gray for µ = 0.11 h^−1^. Half-lives are in reported in minutes. The lines are the rolling averages and thus represent the overall tendency of the data.

The degradation rate of 994, 787 and 996 transcripts were associated with a standard deviation (σ_k_) lower than 30% at 0.11, 0.51 and 0.80 h^−1^ respectively. These mRNAs were further analyzed (see Material & Methods). At a given growth rate, half-lives can be very different from one gene to another and ranged over one order of magnitude. The half-life of a given mRNA was not constant either. It increased with decreasing growth rate : the mean half-lives were 6.2±0.1, 12.1±0.3 and 16.9±0.4, and median half-lives were 5.8, 11.4 and 15.5, at 0.80, 0.51 and 0.11 h^−1^ respectively (figure 2 and table S1). Thus, mRNA stability increased with decreasing growth rate. This correlation is clearly illustrated by the shift of histograms from lower to higher half-lives with decreasing growth rate (figure 2A).

**Figure 3:**
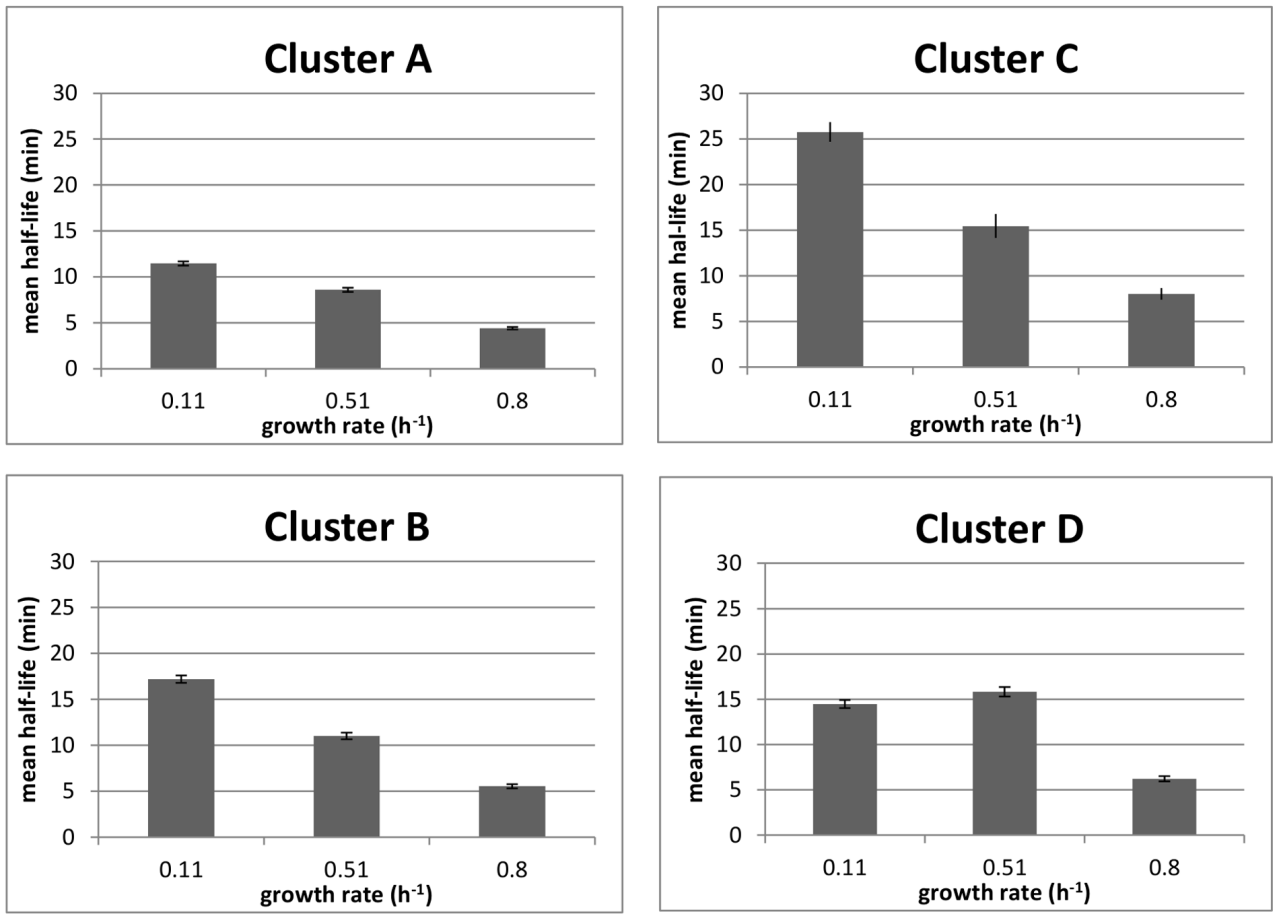
Average mRNA half-life profiles according to classification into the different clusters. X axis: growth rate; Y axis: average half-life; Error bars represent the 5% confidence interval.

The stabilities of the mRNAs for 486 genes were available at all three growth rates: the mean values were of 5.4±0.2, 11.5±0.3 and 14.5±0.4 min at 0.80, 0.51 and 0.11 h^−1^, respectively. This gene subset displays half-life distributions similar to those of the whole population and were therefore used as a representative sample (figure 2B). A hierarchical classification based on the three half-life values of these 486 genes identified four main clusters. Mean stabilities of genes of these different clusters, named clusters A to D are represented in figure 3. Again, the mean half-lives tended to decrease as the growth rate increased (except for the point at 0.51 h^−1^ of cluster D): 73% of these mRNAs were more stable at lower growth rates. Between 0.11 and 0.80 h^−1^, the differences on the average half-lives were about 7, 12 and 17 min for cluster A to C respectively, indicating a stronger growth rate effect from A to C (figure 3). The mRNAs of cluster C were generally more stable than the overall mean stability, independent of the growth rate. Thus, growth rate-dependent stability is a common trait, but the size of the effect varies between mRNAs. Presumably, mRNA half-lives are also affected by gene-specific features.

### Determinants of mRNA Stability

We searched for half-life determinants using a modeling approach. A covariance model was constructed for the 486 mRNAs for which the stability was available at the three growth rates. This type of model was designed to take into account both quantitative and qualitative parameters and has been used successfully to identify determinants of protein abundance [26]. AGGAG in *L. lactis* genes has been identified as a motif stabilizing the mRNA [17]. The number of AGGAG motifs in the upstream regions of the mRNAs (between −100 to +1 bp relative to the translational start codon) was therefore included in the model as a qualitative variable. Functional categories and growth rate values were similarly introduced as qualitative variables. The values of mRNA concentrations were obtained from the t0 time point of our stability assays and from other independent biological repetitions [21], [22]; mRNA concentrations and half-lives were thus measured in rigorously identical conditions and included as quantitative variables. Gene-specific characteristics, including gene length and position, the number of codons, the codon adaptation index (CAI, [27]), the tRNA adaptation index (tAI, [28], [29]) and the GC content, all possibly associated with translational bias were also collected and integrated into the model as quantitative variables. CAI is a measure of codon bias: a number proportional to the frequency of usage in the most expressed proteins (ribosomal proteins) is assigned to each codon; the CAI of a gene is then calculated as the geometric mean of all codons of this gene [27]. tAI is similar to CAI but also takes into account the genomic copy-number of the corresponding tRNA for each codon [28], [29]. The folding free energy (ΔG) of the upstream region of the transcript (from −100 to +1 bp relative to the start codon) was also calculated and included as a qualitative variable: this last value correlates strongly with mRNA secondary structure (the lower the ΔG (*ie.* the more negative), the more strongly folded the structure) which plays a role in translation initiation and in mRNA stability [30], [31].

The Akaike Information Criterion (AIC) was used to select the best model, as that with the most influent variables. [32]. The resulting model, given in table 1, was associated with a high determination coefficient (R^2^) of 0.63. In this model, mRNA concentration and gene length were the most influential quantitative variables. They were both associated with negative coefficients. Both CAI and ΔG were positively correlated with mRNA half-life.

**Table 1:** Covariance model selected with the Akaïke criterion to identify the determinants of mRNA stability.

Selected parameter	Coefficient estimate	Standard error	p-value
[mRNA]	−0.065	0.019	8.10E−4
Length	−0.059	0.013	9.65E−6
ΔG|	0.030	0.012	1.56E−2
CAI	0.029	0.015	4.73E−2
Functional category			
AMI	−0.095	0.068	1.65E−1
CEL	−0.108	0.071	1.28E−1
COF	−0.067	0.072	3.50E−1
ENV	−0.121	0.057	3.50E−2
FAT	−0.143	0.090	1.10E−1
INT	0.202	0.105	5.51E−2
NRJ	0.087	0.044	4.67E−2
OTH	0.023	0.057	6.93E−1
PUR	0.082	0.062	1.81E−1
REG	−0.108	0.044	1.40E−2
REP	0.035	0.066	5.96E−1
TRD	0.108	0.047	2.06E−2
TRS	0.044	0.079	5.68E−1
TSP	0.082	0.041	4.40E−2
Growth rate			
0.11 h^−1^	0.763	0.023	<2.00E−16
0.51 h^−1^	0.189	0.018	<2.00E−16
0.80 h^−1^	−0.952	0.025	<2.00E−16

Light gray parameters were selected by the model but the confidence for their estimated coefficients is not sufficiently high to be considered as significant (p-value>0.05).

AMI = Amino acid biosynthesis, CEL = cellular process, COF = biosynthesis of cofactors, ENV = cell envelope, FAT = fatty acids metabolism, INT = central intermediary metabolism, NRJ = energy metabolism, OTH = other categories, PUR = purines, pyrimidines, nucleosides and nucleotides, REG = regulatory functions, REP = replication, TRD = translation, TRS = transcription, TSP = transport and binding proteins.

The stabilizing motif AGGAG [17] was surprisingly not selected as a significant variable in the model. However, the model was built using all the available half-life data and not only the most stable genes. We used RSAtools to search the upstream region of messengers that were classified as extremely stable [17] for common motifs, and again identified this ribosome binding site-like motif.

All the factors selected in the model (table 1) made a significant contribution to determining mRNA half-life. The relative influence of each quantitative variable is given by the absolute value of the associated parameter. However, the influences of quantitative and qualitative variables cannot be directly compared. To assess the importance of growth rate relative to other factors for determining mRNA half-life, three different models (one for each growth rate) were constructed without this variable. This led to a substantially lower associated R^2^: maximum 0.06 (compared to 0.63 when growth rate was included), demonstrating that growth rate is a very major determinant of mRNA stability. The variable functional category was also retained in the final model, highlighting a link between mRNA half-life and cellular function. The coefficients and significances related to the qualitative parameters indicate, in particular, that the mRNAs of genes with growth-related functions, (such as those classified in the energy and translation functional groups) are generally more stable than others. The mRNAs of genes coding for proteins with regulatory functions are among the least stable. Fine tuning of this determinant analysis by covariance modeling of mRNA stability in each of the four clusters identified (data not shown) provided further evidence of the sensitivity of mRNA stability to growth rate. It also identified determinants specific for some of the clusters (*i.e.* GC % in cluster B, gene position in cluster C and the number of RBS-like sequences in genes in cluster D). These observations should, however, be interpreted with caution, because of the numbers of genes included in the corresponding partial models were small.

### Antagonistic Roles of Transcription and Stability on mRNA Intracellular Concentration

The cellular concentration of mRNA can be changed by modification of mRNA synthesis or elimination rates. mRNA synthesis is governed by transcription, whereas mRNA can be eliminated by degradation by ribonucleases and dilution as cells divide. Transcription, decay and growth dynamics therefore need to be taken into account when analyzing the control of the mRNA pool. To evaluate the contributions of these processes on the regulation of the mRNA concentration, we used a regulation analysis method [17], derived from metabolic control analysis formalism [33], [34]. Briefly, assuming that a steady-state is established, the following equation can be written: 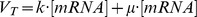 (1), where V_T_ is the transcription rate, [mRNA] the transcript concentration, µ the growth rate and k the degradation rate constant inversely proportional to the mRNA half-life (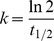 (2)). The rate of dilution of messengers due to cellular growth can be neglected in this context, because the generation time is usually very much longer than mRNA half-lives (this assumption was true, at all growth rates, for more than 85% of the measured stabilities). Assuming that V_T_ and k are independent, a difference in mRNA concentration between two steady-states can be described as a function of the differences in the transcription and degradation rates: 
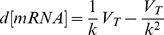
 (3). This equation is mathematically equivalent to: 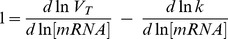 (4). Two regulation coefficients were defined from equation (4): ρ_D_, the degradation coefficient which represents the influence of decay on the mRNA pool 
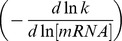
 and ρ_T_, the transcription coefficient which describes the influence of the transcription rate on the mRNA pool 
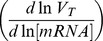
. The degradation regulation coefficient ρ_D_ can be calculated as the opposite slope of the double-logarithmic plot of the degradation rate constant k *versus* the initial mRNA concentration (before rifampicin treatment) in the two conditions studied. The transcription coefficient ρ_T_ can then be estimated from ρ_D_ as 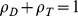 (5).

**Figure 4:**
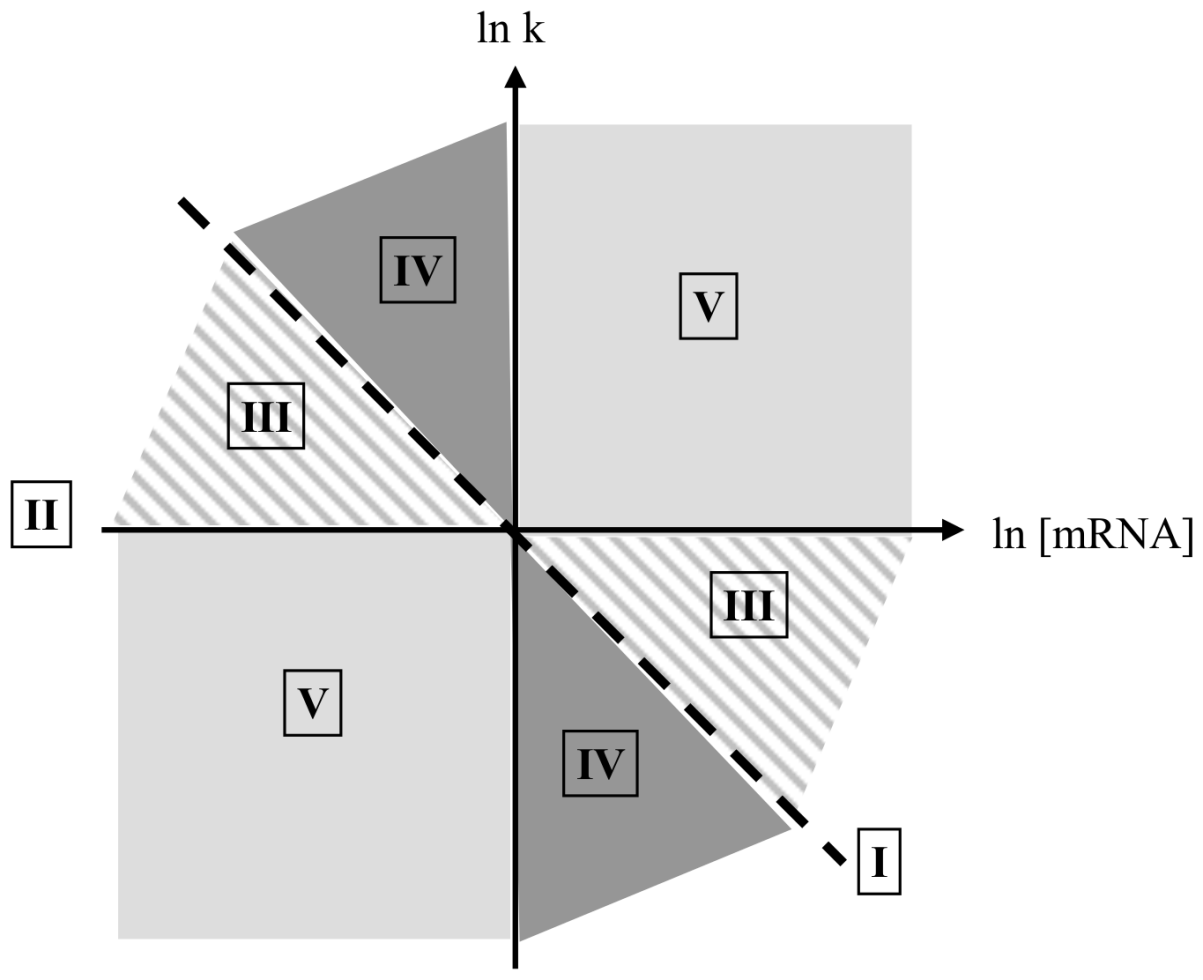
Graphical representation of the different modes of gene expression control. This plot of lnk *versus* ln[mRNA] represents, for each mRNA, the modulations of these parameters between two conditions (*i.e.* different growth rates). The opposite slope of this plot corresponds to the degradational regulation coefficient ρ_D_. Control solely by degradation is represented by the dotted line (I), control solely by transcription is associated to the x-axis (II). Slopes grouped in the hatched, dark gray and light gray areas reveal respectively shared (III), mainly degradation-related (IV) and mainly transcription-related (V) control.

Five patterns of regulation, numbered I to V, could be distinguished according to the value of ρ_D_ (see the double-logarithmic plot of the degradation constant rate k *versus* mRNA concentration in figure 4). I - Control solely by degradation - If ρ_D_ equals 1 (equivalent to ρ_T_ = 0), changes in mRNA concentration are directly proportional to changes in the rate of degradation. Consequently, the mRNA concentration is regulated exclusively by degradation and transcription makes no contribution to this regulation. II - Control solely by transcription - When ρ_D_ equals 0 (equivalent to ρ_T_ = 1), mRNA stability is constant *i.e.* no stability control occurs. The regulation of the mRNA pool is therefore exclusively transcriptional. III - Shared control - When ρ_D_ displays values between 0 and 1, ρ_T_ values are also between 0 and 1. Hence, the mRNA pool is regulated by changes in both transcription and degradation having the same effects: increased mRNA levels associated with transcription rate increases and degradation constant decreases, and the inverse. IV - Mainly degradational control (ρ_D_ >1) and V - Mainly transcriptional control (ρ_D_<0) - In these two lasts cases, the changes in transcription and degradation have the opposite effects on the transcript level: when transcription rate increases, the degradation rate also increases and *vice versa*. In case IV, for example, the mRNA concentration increases due to a reduced degradation rate (mRNA stabilization) in the presence of a reduced transcription rate; the mRNA pool is mainly controlled by degradation although a smaller transcription effect in the opposite direction is also present. Similarly, in case V, the mRNA concentration increases as the result of a higher transcription rate associated with a smaller increase of the degradation rate; the major regulation by transcription is in part counteracted by decreased transcript stability thereby limiting the change in the mRNA pool.

Table 2 (upper part, chemostat) summarizes the number of genes with a given type of control explaining the differences in mRNA concentration between two growth conditions. Most of the genes displayed ρ_D_>1 or <0 (cases IV and V) and only 1 to 14% of the genes exhibited a coordinated control between transcription and degradation (case III). The major control was exerted at the transcriptional level with 84 to 98% of the genes with ρ_D_≤0. Even though degradational regulation attenuates its effect, transcription thus clearly dominates the control of mRNA concentrations. For example, for a gene exhibiting different mRNA concentrations at two different growth rates, a ρ_D_<0 indicates major transcriptional control and thus that difference in mRNA concentration is due to the difference in transcription rate, despite smaller differences in stability. Consequently, the reduction of transcription rate must be significantly higher than the decrease of degradation rate to ensure transcript down-expression and vice-versa. Most of the differences in transcript concentrations could be explained mainly by transcriptional control. However, the differences of four transcripts (*busAA, mreC, serA* and *yghD*) between 0.80 and 0.51 h^−1^ and 14 (*busAA, fadD, gidA, guaC, hflX, hmcM, ispB, nah, ybdK, ybeH, ybeM, yfcG, yobA* and *yphA*) between 0.51 and 0.11 h^−1^ were controlled by degradation. There are no evidence characteristics or features of those genes that could explain this mode of regulation. The levels of the *busAA* gene transcript (a messenger encoding a betaine ABC transporter) were driven by degradation in both cases.

**Table 2:** Main mechanisms controlling mRNA concentrations.

	Compared cultures	Mainly transcriptional control ρ_D_ ≤0		Shared control 0<ρ_D_<1		Mainly degradation control ρ_D_ ≥1	
		Number of genes	Percentage of the total	Number of genes	Percentage of the total	Number of genes	Percentage of the total
Chemostat	0.80 *vs.* 0.51 h^−1^	479	98%	4	1%	4	1%
	0.51 *vs.* 0.11 h^−1^	454	84%	75	14%	14	3%
Batch	Exponential phase (0.8 h^−1^) *vs.* deceleration (0.38 h^−1^)	452	72%	109	17%	67	11%
	Deceleration (0.38 h^−1^) *vs.* stationary (0.04 h^−1^)	490	77%	66	10%	77	12%

*vs.* indicates the growth conditions considered for the calculation of the regulation coefficients. Chemostat and batch indicate that RNA half-lives were determined in continuous or discontinuous cultures, respectively. In chemostat cultures, cells were at a steady state, with growth limited by the isoleucine concentrations. In batch cultures, isoleucine in the medium was progressively consumed (until starvation was reached) and the cells were in a dynamic process of adaptation.

Cells grown in chemostat are in a steady-state; they are fully adapted to their environment. This method of cultivation allows growth parameters such as pH, temperature or nutrient limitation, to be studied one by one. We took advantage of this unique property to specifically study the effect of growth rate on the mRNA levels [21], [22] and stabilities of *L. lactis* (this study). In this work, we showed that, when only growth rate varies, mRNA levels are mainly controlled by transcription rates and that degradation act antagonistically to transcription. In their natural environment, cells have to cope with multiple and often simultaneous changes at the same time: the environment is dynamic and cells are constantly adapting (unlike the situation in chemostat cultures). We therefore investigated the regulation of mRNA concentrations in cells during dynamic adaptation.

**Figure 5:**
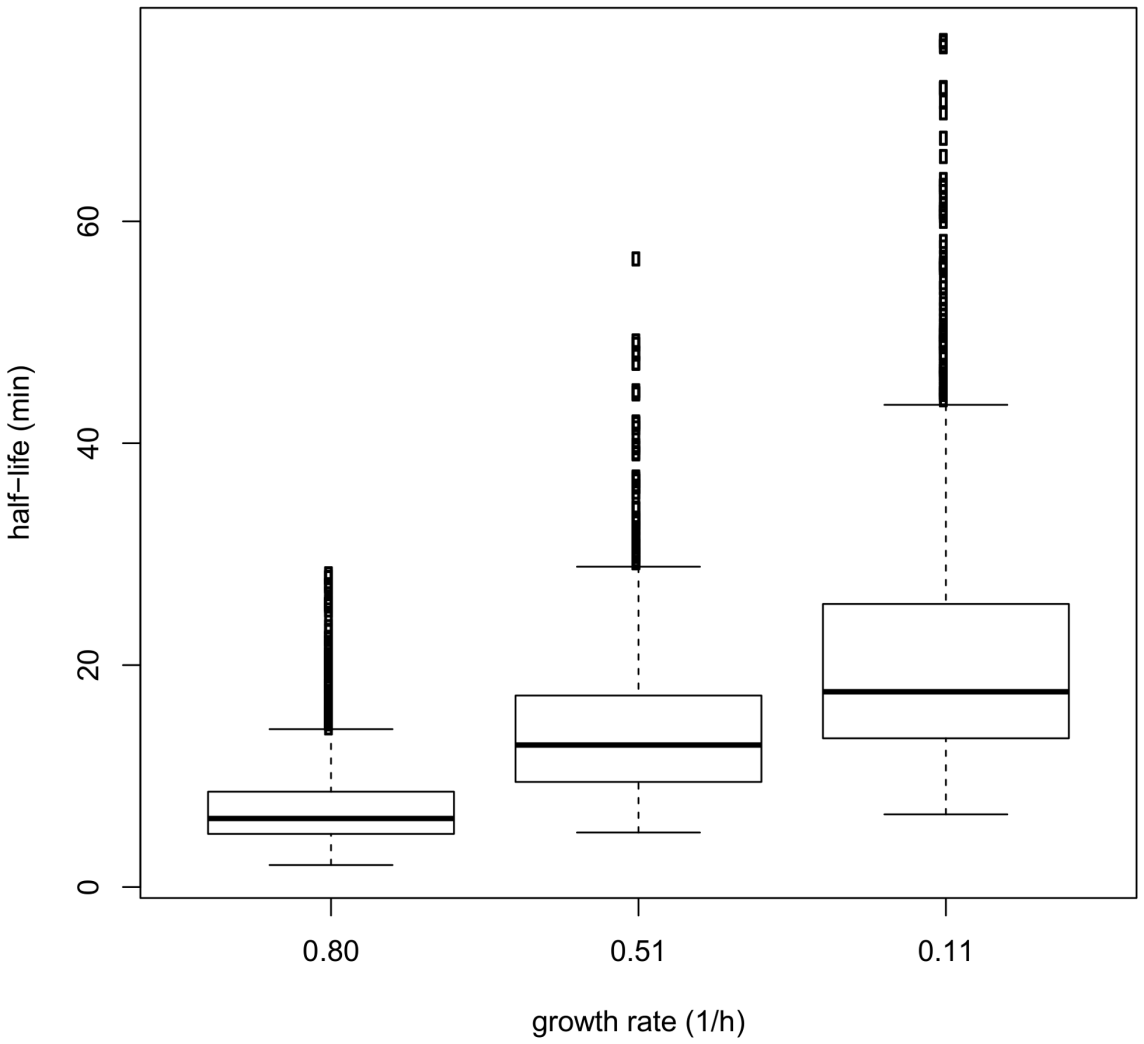
Box plot representation of mRNA half-life distribution during the progressive adaptation to isoleucine starvation (batch). This box plot represents the half-life values in four quartiles separated by horizontal bars. The central bar (in the middle of the rectangle) represents the median value.

We studied mRNA stability during the adaptation of *L. lactis* to progressive isoleucine starvation in batch culture. We have already reported transcriptome and proteome analyses of this progressive adaptation [35]. In the present work, the growth conditions described previously were reproduced and mRNA half-lives were measured during the exponential phase (0.8 h^−1^), as the growth rate was decreasing (0.38 h^−1^) and in the pseudo-stationary phase (0.04 h^−1^): the mean values were 6.24, 7.63 and 12.16 and median values 5.75, 7.49 and 11.11, respectively, again showing a significant effect of the growth rate on mRNA stability (figure 5). Application of the control coefficient theory, however, showed that in these specific conditions of progressive adaptation to isoleucine starvation, control by degradation (the case for 11 to 12% of the genes) was stronger than when only the growth rate was altered (Table 2, lower part, batch, compared to upper part, chemostat). We previously demonstrated that transcription was the main mechanism controlling mRNA levels during the transition between exponential and deceleration phases when *L. lactis* was progressively subjected to carbon starvation [17]. The results were different during the adaptation to isoleucine starvation. The influence of shared (0<ρ_D_<1) and degradational (ρ_D_ >1) controls was not negligible during the transition between deceleration and starvation phases, and these mechanisms applied to 22 to 28% of the mRNAs.

## Discussion

The wide range of values (two orders of magnitude) covered by the observed half-lives of *L. lactis* messengers is in agreement with previous findings for bacteria [12], [13], [14], [17]. This indicates that mRNA stability cannot be considered to be a constant for all transcripts. The preponderance of the “other” functional category, and particularly transposon- and phage-related transcripts, among the very stable mRNAs suggests that the cellular degradation machinery is specific for endogenous RNA. This is consistent with exogenous and/or recently (laterally) acquired genes being less efficiently degraded, which could confer a selective advantage as was recently demonstrated in *E. coli* [36]. Another possibility is that regulatory mechanisms are not required for genes that are expressed in a burst, as is usually the case for phage genes.

Bernstein *et al.* did not identify any significant differences in mean half-lives of mRNAs between *E. coli* grown at high growth rate (rich medium) and low growth rate (poor medium) [12]. However, the initial study of *L. lactis* mRNA stability showed longer half-lives during the carbon starvation phase than during the exponential phase [17]. Similarly, Shalem *et al.* evidenced modulation of mRNA stability in yeast during the stress response [18]. These various studies strongly argue for the involvement of mRNA degradation processes in the adaptation of microorganisms to their environments. Changes in these processes could be related either to the stress effect or to a growth rate effect or to both. Our results clearly demonstrate, for the first time, that the modulation of the growth rate alone is sufficient to affect mRNA stability.

The significant inverse relationship between messenger half-lives and their respective concentrations corroborates the results reported by Redon *et al.* [17]. Thus, the most concentrated mRNAs are the least stable and transcription and degradation have opposite effects. This argues in favor of the hypothesis previously raised stating that mRNA degradation tends to counterbalance transcription [18], [37]. The negative correlation between mRNA stability and length suggests that, as in other bacteria, transcript decay in *L. lactis* involves endonucleolytic cleavage [10], [38]. If endonucleolytic cleavage sites are evenly distributed in RNA, longer transcripts would be expected to contain more sites and thus be more prone to degradation than shorter transcripts. It is generally assumed that there is a mechanism in bacteria by which ribosomes protect mRNA from degradation [39], [40]. The tRNA adaptation index (tAI) is a derivative of the CAI taking the tRNA copy-number into account. In *E. coli*, the tAI was found to correlate negatively with mRNA stability [36]. This finding was attributed to there being less ribosome stalling in the presence of optimal codons, and therefore less protection of the RNA by stalled ribosomes. We report for *L. lactis* a positive correlation between CAI and mRNA half-life, and thus the opposite of the relationship described in *E. coli*. This may reflect a fundamental divergence in mRNA decay mechanisms between Gram-positive and Gram-negative bacteria, leading for example in *B. subtilis* to a single stalled ribosome protecting the mRNA from RNase cleavage for kilobases downstream [39], [40]. Possibly, the ribosome protective effect in *L. lactis* is less dependent on the number of stalled ribosomes than on their strategic position. This is supported by recent polysome profiling experiments in *L. lactis* showing that the most ribosome-crowded transcripts were not the most stable [41]. The more negative ΔG is (*i.e.* the higher the absolute value), the more structured the mRNA is expected to be. The positive correlation between mRNA half-life and the absolute value of ΔG indicates that the most strongly structured messengers may be the least prone to degradation. This seems logical because structured mRNAs are less accessible to the degradation machinery [42]. The extremely low determination coefficient (R^2^) associated with models in which growth rate was not included clearly demonstrates that this variable has the largest influence on mRNA half-life. The high stability of the transcripts encoding proteins with housekeeping functions, such as those involved in energy supply and translation, may reflect their physiological importance. Ribosomal proteins (major proteins of the functional category ‘translation’) are the most abundant proteins in cells and limiting the degradation of their transcripts favors the constitution of a backup pool ready for translation when more protein is required. By contrast, the abundance of regulatory proteins needs to be rapidly adapted to environmental changes to ensure appropriate regulation. Therefore, modulation through mRNA degradation allows a post-transcriptional control of the levels of such proteins; it is faster and less energy-demanding than direct protein synthesis or protein degradation [43].

The control analysis proved to be a powerful method for determining whether transcription- or degradation-related mechanisms had a larger influence on the regulation mRNA levels, without requiring time-consuming experimental determination of transcription rates for individual genes. Moreover, to our knowledge, no genome-wide methods to determine transcription rate have been described. Our study demonstrates that, as the growth rate changes in *L. lactis*, the change in the transcription rate is antagonised by the change in the degradation rate. This finding is counter-intuitive but was previously proposed to occur in yeast [18], [37]. It most likely allows fine tuning of mRNA pool. The general stabilization of mRNAs observed when the growth rate decreases is thus accompanied by a large decrease of the transcription rate. This is consistent with our previous observation that the lower the growth rate, the lower the intracellular concentration of messengers [22]: mRNA stabilization may therefore partially compensate for the decrease in the transcription rate to limit the diminution of transcript concentration. This type of regulatory pattern may minimize energy consumption while ensuring a sufficient mRNA pool.

The control of mRNA levels differed according to the growth conditions (*i.e.* adapted cells in chemostat, or cells subjected to stress conditions in batch cultures). The relative influence of degradation on transcript level regulation was larger in cells in the process of adapting to stress than in cells fully adapted to their environment. There appears to be a link (independent of the growth rate) between messenger stability regulation and the dynamics of the stress response. Further investigations are required to confirm and clarify this link, which may involve the degradation machinery (the levels of some RNases are stress-dependent) [10]. The distribution of the different regulatory mechanisms (transcription-related, shared and degradation-related controls) seems to depend on the nutritional/environmental conditions the kinetics differ between situations of carbon and nitrogen starvation [17]. These various findings and analyses underline the crucial role of mRNA stability modulation during adaptation to stress and the importance of this phenomenon.

### Conclusions

Global measurement of *L. lactis* mRNA half-lives at different growth rates under isoleucine-limited conditions led to the first formal demonstration that the growth rate determines mRNA degradation rates. The results clearly revealed a large effect of the growth rate with higher degradation rates at high growth rates. Covariance modeling of mRNA half-lives indicated that growth rate was the main determinant of mRNA stability, but also that mRNA concentration, length and codon adaptation index made significant contributions to determining the mRNA degradation rate. Regulation analysis formalism was used to provide insights into the regulation of gene expression. It revealed a very weak control of the mRNA pool by degradation-related processes when only the growth rate was varied (chemostat cultures) but a larger effect of this regulatory mechanism during adaptation to isoleucine starvation (batch cultures). However expression variations related to transcription rate changes were in most of the cases antagonised by the change in the degradation rate.

## Materials and Methods

### Strain and Growth Conditions

*Lactococcus lactis ssp. lactis* IL1403, whose genome has been entirely sequenced [25], was grown as previously described [21]. Briefly, continuous cultures were performed in chemically defined medium with low Branched-Chain Amino Acid (BCAA; *i.e.*, valine, leucine, isoleucine, exact composition given in table S2) concentrations leading to isoleucine-limited growth. A 0.5 L bioreactor (Verrerie Wagner, Toulouse, France) was maintained at a constant temperature of 30°C and under a nitrogen atmosphere. The pH was regulated at 6.6 by automatic addition of KOH (10 N). Two different growth rates were studied, namely 0.11 and 0.51 h^−1^, corresponding to doubling times of 6.30 h and 1.36 h, respectively. The minimum interval between collections of samples was five doubling times.

To study mRNA stability during isoleucine starvation response, *L. lactis* was grown in batch cultures in the same medium as the continuous cultures, as previously described [35]. Cultures were grown under a nitrogen atmosphere in a 2 L bioreactor (Setric Génie Industriel, Toulouse, France) at 30°C. mRNA half-lives were measured during the exponential phase (µ = 0.8 h^−1^, doubling time of 0.87 h), while the growth rate was decreasing (µ = 0.38 h^−1^, doubling time of 1.82 h) and during the pseudo-stationary phase (µ = 0.04 h^−1^, doubling time of 17.32 h).

### Transcriptome-based mRNA Stability Measurements

This method was adapted from that used to study *L. lactis* mRNA stability during carbon starvation [17]; its principles are explained in figure S1. Briefly, when the desired steady-state was reached, transcription was stopped by addition of rifampicin to a final concentration of 500 µg.mL^−1^. Cell samples were harvested immediately before and during 20 minutes after antibiotic addition. All the samples were then treated in parallel including the reference point before rifampicin addition. The transcriptome experiment protocols were followed: RNA extraction, cDNA preparation, hybridization and detection. At least two independent biological experiments were performed and two methodological replicates.

Total RNA was quantified by measuring the OD at 260 nm and RNA quality was checked on an Agilent Bioanalyzer®. Transcript levels were measured using nylon macro-arrays containing ∼800 bp-length *L. lactis* IL1403 gene-specific PCR fragments (EUROGENTEC). The same amount, 15 µg, of total RNA was used to for all reverse transcription experiments. Synthesis of radiolabeled cDNA, nylon array hybridization and washing were performed as previously described [17]. Membranes were exposed to a phosphor-imager screen for three days and scanned with a phosphor-fluoro-imager (Storm 860, Molecular Dynamics).

Raw data were processed to access mRNA concentrations and were normalized to the mean intensity of the reference membrane (before rifampicin addition) for each batch. mRNA half-lives (t_1/2_) were calculated from the degradation rate constant (k), which corresponds to the slope of mRNA concentration logarithm as a function of time with the relation 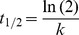 (6) (see figure S1); mRNA dilution after rifampicin addition can be neglected and the following equation can be written (figure 1): 
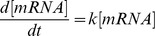
 (7) hence 
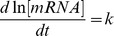
 (8). The standard deviations (σ_k_) associated to the slopes were calculated for the data from the four repeat experiments combined. Half-life values were considered as reliable only if σ_k_ ≤30%. A group of transcripts that were extremely stable was also identified: those with horizontal slopes, corresponding to σ_k_ ≥70%. The time-course of the experiment was too short to allow accurate calculation of the t_1/2_ values of these mRNAs, and consequently they were not included in the analysis.

### Clustering Method

R free statistical software (www.r-project.org) with the Ward classification method was used for clustering. The number of classes was determined graphically from the dendrogram.

### Motif Research

The presence of DNA patterns in untranslated regions of genes was explored using RSAtools software (http://rsat.ulb.ac.be/rsat/). The sequences were also obtained from RSAtools (retrieve sequence section, default parameters).

### Folding Free Energy Calculation

The upstream mRNA sequences (from −100 to +1 bp relative to the start codon) were also obtained from RSAtools and processed with RNAfold software (http://mobyle.pasteur.fr/cgi-bin/portal.py) specifying a temperature of 30°C. For each sequence, we used the free energy of the predicted minimum free energy structure (the most negative ΔG) as a measure of secondary structure formation.

### Data Availability

Raw data were deposited on Gene Expression Omnibus data repository (GSE43875, http://www.ncbi.nlm.nih.gov/geo/query/acc.cgi?acc=GSE43875). Half-lives of individual genes in the different conditions studied are given in Table S1.

## Supporting Information

Figure S1 **Principles of the method to determine individual mRNA half-life.** Transcription is stopped by the addition of rifampicin at time 0. Degradation is then considered to become the major phenomenon responsible for lowering mRNA levels. The concentration of each single mRNA is monitored over time after rifampicin addition. The slope resulting from the semi-logarithmic plot of ln[mRNA] versus time gives access to the degradation rate (k) that is directly linked to half-live (t1/2).
(PDF)

Table S1 **Value of half-lives for** ***L. lactis*** **transcripts at the different growth rates studied NA indicates that the probe corresponding to the gene is not available on the micro-array**. ND stands for Not Determined and represents mRNA associated with 30% ≤ σ_k_ ≤70%. Very stable genes display σ_k_ ≥70% and their half-life could not be accurately determined.
(PDF)

Table S2 **Composition of the growth medium.**
(PDF)
