# Supplementary figures and images for: Role of mRNA Stability during Bacterial Adaptation
Source: PLoS One. 2013 Mar 13;8(3):e59059. doi: 10.1371/journal.pone.0059059 (PMC3596320; doi:10.1371/journal.pone.0059059)

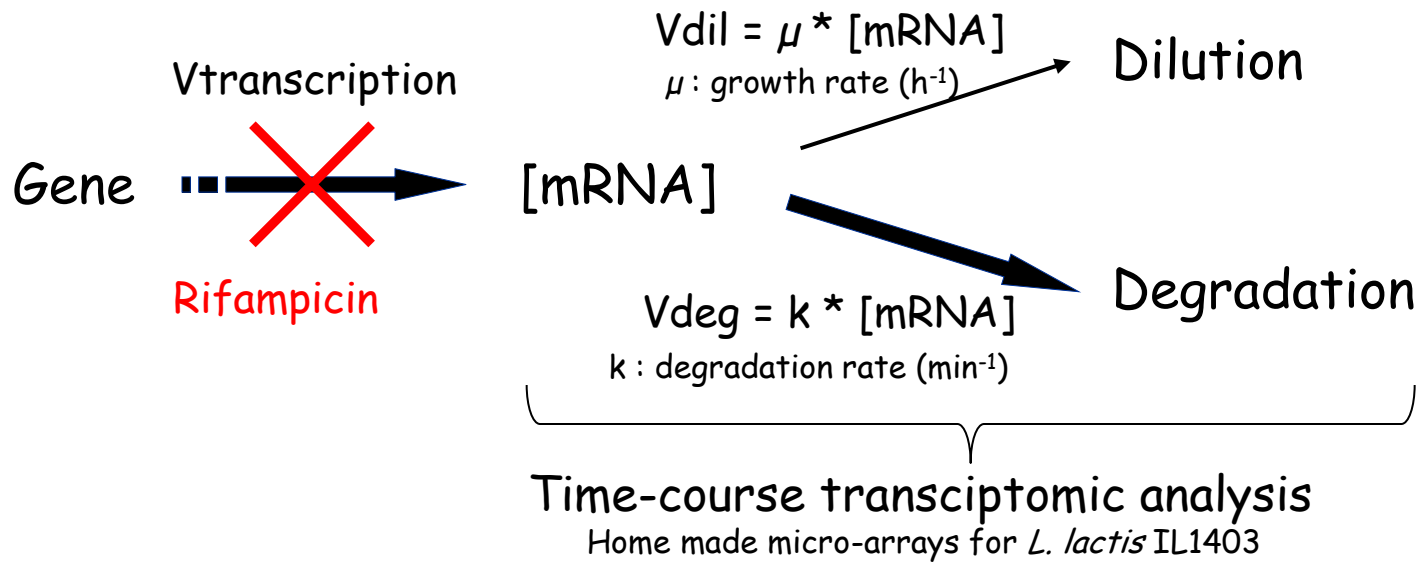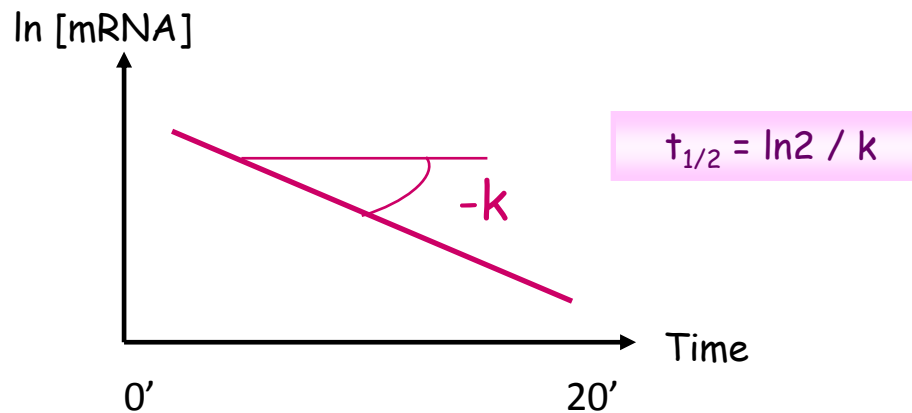

Supplement: Figure S1 — Principles of the method to determine individual mRNA half-life. Transcription is stopped by the addition of rifampicin at time 0. Degradation is then considered to become the major phenomenon responsible for lowering mRNA levels. The concentration of each single mRNA is monitored over time after rifampicin addition. The slope resulting from the semi-logarithmic plot of ln[mRNA] versus time gives access to the degradation rate (k) that is directly linked to half-live (t1/2). (PDF) [file pone.0059059.s001.pdf]
