# Supplementary material for: Role of mRNA Stability during Bacterial Adaptation
Source: PLoS One. 2013 Mar 13;8(3):e59059. doi: 10.1371/journal.pone.0059059 (PMC3596320; doi:10.1371/journal.pone.0059059)
Supplement: Table S1 — Value of half-lives for L. lactis transcripts at the different growth rates studied NA indicates that the probe corresponding to the gene is not available on the micro-array. ND stands for Not Determined and represents mRNA associated with 30% ≤ σk ≤70%. Very stable genes display σk ≥70% and their half-life could not be accurately determined. (PDF) [file pone.0059059.s002.pdf]

| gene | $t_{1/2}$ (min) at μ=0.11h$^{-1}$ | $t_{1/2}$ (min) at μ=0.51h$^{-1}$ | $t_{1/2}$ (min) at μ=0.80h$^{-1}$ | $t_{1/2}$ (min) at μ=0.38h$^{-1}$ | $t_{1/2}$ (min) at μ=0.04h$^{-1}$ |
|---|---|---|---|---|---|
| accA | NA | NA | NA | NA | NA |
| accB | 14.73 | 17.07 | 9.90 | ND | ND |
| accC | 9.63 | 13.38 | 4.50 | ND | 6.02 |
| accD | 13.48 | ND | 6.13 | 6.98 | ND |
| ackA1 | 14.38 | 14.66 | 5.18 | ND | ND |
| ackA2 | 14.53 | ND | 6.90 | ND | ND |
| acmA | 17.34 | 19.19 | 4.92 | 6.91 | 5.23 |
| acmB | 13.70 | 8.98 | 4.80 | ND | 5.06 |
| acmC | 11.60 | 6.49 | 4.73 | ND | ND |
| acmD | 10.23 | 7.29 | 3.75 | 5.85 | 4.34 |
| acpA | very stable | very stable | ND | ND | ND |
| acpD | 13.87 | 16.43 | 4.83 | 6.64 | ND |
| acpS | NA | NA | NA | NA | NA |
| adaA | 13.29 | 15.99 | 5.87 | ND | ND |
| add | 15.77 | 7.47 | 5.20 | 6.99 | ND |
| adhA | NA | NA | NA | NA | NA |
| adhE | 13.30 | 18.80 | 4.59 | ND | 5.92 |
| adk | 16.93 | 12.05 | 5.79 | 9.17 | 5.02 |
| agl | NA | NA | NA | NA | NA |
| ahpC | 20.26 | ND | 8.14 | 7.81 | ND |
| ahpF | 20.79 | ND | 9.84 | 9.52 | ND |
| ahrC | 21.51 | 18.77 | 9.07 | ND | ND |
| alaS | 17.95 | 12.90 | 6.69 | ND | ND |
| aldB | 45.25 | ND | ND | ND | 7.88 |
| aldC | NA | NA | NA | NA | NA |
| aldR | very stable | ND | very stable | ND | ND |
| als | 9.79 | 6.30 | 3.25 | 3.89 | ND |
| amtB | 12.70 | 10.34 | ND | ND | ND |
| amyL | 12.79 | 10.33 | 5.03 | 5.42 | ND |
| amyY | NA | NA | NA | NA | NA |
| ansB | 33.86 | ND | 14.31 | ND | ND |
| apbE | 37.19 | 29.98 | ND | ND | ND |
| apl | very stable | very stable | very stable | ND | ND |
| apt | 29.54 | 13.32 | 5.95 | ND | ND |
| apu | ND | very stable | very stable | ND | ND |
| araT | 10.05 | 9.38 | 3.91 | ND | 5.82 |
| arcA | 22.00 | very stable | ND | ND | ND |
| arcB | 17.47 | very stable | very stable | ND | ND |
| arcC1 | 30.52 | very stable | very stable | ND | ND |
| arcC2 | ND | very stable | very stable | ND | ND |
| arcC3 | 45.97 | very stable | very stable | ND | ND |
| arcD1 | very stable | ND | very stable | ND | ND |
| arcD2 | 27.17 | 13.87 | 7.50 | ND | ND |
| arcT | very stable | ND | very stable | ND | ND |
| argB | NA | very stable | NA | NA | NA |
| argC | 42.01 | very stable | NA | NA | NA |
| argD | very stable | ND | NA | NA | NA |
| argE | 69.75 | 17.39 | ND | ND | ND |

| | | | | | |
|---|---|---|---|---|---|
| argF | ND | ND | very stable | ND | ND |
| argG | very stable | very stable | very stable | ND | ND |
| argH | ND | very stable | ND | ND | ND |
| argJ | ND | ND | ND | ND | ND |
| argR | 16.71 | 8.84 | 4.30 | ND | ND |
| argS | 21.12 | 12.96 | 4.86 | 7.08 | 5.20 |
| aroA | 9.60 | 11.15 | 4.51 | ND | ND |
| aroB | 36.50 | very stable | 9.13 | 8.87 | ND |
| aroC | 11.67 | ND | 6.06 | 5.42 | ND |
| aroD | 8.99 | 5.27 | 5.97 | ND | ND |
| aroE | 14.86 | 9.63 | 6.56 | ND | ND |
| aroH | 13.24 | 8.90 | 3.66 | 6.79 | 5.37 |
| aroK | NA | NA | 8.05 | ND | ND |
| arsC | 17.56 | 13.77 | 8.94 | ND | ND |
| asd | 7.86 | 5.22 | 2.83 | ND | 4.45 |
| asnB | 11.43 | 9.36 | 4.95 | ND | ND |
| asnH | 10.57 | 7.81 | 4.63 | ND | ND |
| asnS | 12.58 | very stable | NA | NA | NA |
| aspB | 13.90 | ND | 5.23 | 5.34 | 6.53 |
| aspC | 16.38 | ND | 6.66 | 9.10 | 7.73 |
| aspS | 10.04 | 17.55 | 4.88 | 6.34 | ND |
| atpA | NA | NA | NA | NA | NA |
| atpB | 22.38 | 18.08 | 9.66 | 8.57 | ND |
| atpD | 23.37 | ND | 11.04 | ND | ND |
| atpE | 36.80 | ND | very stable | ND | ND |
| atpF | 17.42 | ND | 12.32 | ND | ND |
| atpG | 19.30 | ND | 10.71 | 8.60 | ND |
| atpH | 14.89 | ND | 8.94 | 8.46 | ND |
| bacA | 13.32 | 9.58 | 5.77 | ND | ND |
| bar | ND | very stable | 24.94 | 6.33 | ND |
| bcaT | 10.34 | 10.28 | 4.73 | 4.34 | ND |
| bglA | very stable | very stable | very stable | ND | ND |
| bglH | 14.56 | ND | 4.84 | ND | ND |
| bglR | very stable | very stable | very stable | ND | ND |
| bglS | 33.03 | ND | 14.72 | ND | ND |
| birA1 | 15.40 | 9.25 | 4.66 | ND | ND |
| birA2 | 17.29 | 10.56 | 5.33 | ND | ND |
| blt | 26.08 | 28.34 | 22.48 | ND | ND |
| bmpA | 12.34 | 8.24 | 6.82 | 8.94 | ND |
| busAA | 11.35 | 15.69 | 7.38 | 6.22 | ND |
| busAB | 12.91 | ND | very stable | ND | ND |
| busR | 15.46 | 13.87 | ND | ND | ND |
| butA | 25.55 | ND | 5.23 | ND | ND |
| butB | 21.56 | very stable | ND | ND | ND |
| cadA | 15.77 | very stable | 8.68 | ND | ND |
| carA | 13.89 | 20.17 | 6.17 | 9.11 | ND |
| carB | 29.30 | 13.87 | 5.50 | 12.29 | ND |
| cbr | 26.63 | ND | ND | ND | ND |
| ccpA | 11.26 | 8.65 | 4.04 | ND | 5.63 |
| cdd | 27.03 | very stable | 5.09 | ND | ND |

| | | | | | |
|---|---|---|---|---|---|
| cdsA | 10.00 | 10.36 | 5.29 | ND | ND |
| celB | ND | ND | 10.88 | ND | ND |
| ceo | 22.71 | ND | very stable | ND | ND |
| chiA | 14.58 | ND | 11.86 | ND | ND |
| choQ | NA | NA | 7.01 | 5.19 | ND |
| choS | ND | ND | very stable | ND | ND |
| citB | 18.38 | very stable | ND | 9.95 | ND |
| citC | 16.92 | very stable | 7.41 | 9.84 | ND |
| citD | 17.84 | ND | ND | ND | ND |
| citE | ND | very stable | ND | ND | ND |
| citF | 28.45 | very stable | ND | ND | ND |
| citR | 18.35 | 14.45 | 4.56 | 5.13 | 5.10 |
| clpB | 13.09 | 28.72 | 6.64 | 5.31 | ND |
| clpC | 11.45 | 9.62 | 6.01 | 7.68 | ND |
| clpE | 13.82 | ND | 5.81 | 6.66 | ND |
| clpP | 12.05 | 20.48 | 5.74 | 5.19 | ND |
| clpX | 25.89 | 32.92 | 15.79 | 8.07 | ND |
| clsA | 13.81 | 16.65 | 6.66 | 10.42 | ND |
| clsB | 44.36 | very stable | very stable | ND | ND |
| cmk | 26.81 | 16.44 | 9.47 | ND | 7.47 |
| coaA | 12.63 | 6.80 | 3.74 | ND | 4.96 |
| cobC | 11.46 | 7.66 | 4.54 | ND | ND |
| cobQ | 10.40 | 14.71 | 4.31 | 6.48 | ND |
| codY | 11.20 | 7.04 | 3.34 | 4.70 | 3.98 |
| codZ | 42.86 | ND | very stable | ND | ND |
| coiA | NA | ND | ND | ND | ND |
| comC | 30.25 | 39.01 | 8.31 | ND | ND |
| comEA | very stable | 34.12 | very stable | ND | ND |
| comEC | very stable | very stable | very stable | ND | ND |
| comFA | very stable | ND | very stable | ND | ND |
| comFC | NA | NA | NA | NA | NA |
| comGA | ND | very stable | ND | ND | ND |
| comGB | 23.66 | very stable | very stable | ND | ND |
| comGC | 63.21 | very stable | very stable | ND | ND |
| comGD | very stable | very stable | very stable | ND | ND |
| comX | very stable | very stable | very stable | ND | ND |
| copA | NA | NA | 13.88 | ND | ND |
| copB | NA | NA | ND | ND | ND |
| copR | 10.22 | 17.34 | 6.31 | ND | ND |
| cpo | 37.64 | very stable | 7.83 | 9.23 | ND |
| cpsM | ND | ND | ND | 10.09 | ND |
| crtK | 21.25 | very stable | ND | ND | ND |
| cshA | 11.58 | ND | 4.90 | ND | ND |
| cspE | 8.26 | 5.59 | very stable | ND | ND |
| cstA | 25.92 | ND | ND | ND | ND |
| ctrA | 11.85 | 12.13 | 4.39 | 5.64 | 4.94 |
| ctsR | 33.54 | 24.75 | 15.02 | ND | ND |
| cydA | 21.01 | ND | 6.50 | ND | ND |
| cydC | 25.55 | 18.20 | ND | ND | ND |
| cydD | 39.33 | very stable | ND | ND | ND |

| | | | | | |
|---|---|---|---|---|---|
| cysD | 32.07 | ND | very stable | ND | ND |
| cysE | 13.84 | 15.93 | 5.74 | 7.33 | 5.72 |
| cysK | very stable | very stable | 13.79 | ND | ND |
| cysM | ND | 25.52 | 8.20 | ND | ND |
| cysS | very stable | ND | very stable | ND | ND |
| dacA | very stable | ND | very stable | ND | ND |
| dacB | 30.28 | ND | 11.77 | 8.74 | 8.39 |
| dal | 28.61 | very stable | 10.98 | 10.23 | ND |
| dapA | 24.53 | very stable | 7.37 | 9.25 | 9.63 |
| dapB | 27.11 | ND | 5.48 | 8.90 | 6.42 |
| dcdA | 34.62 | very stable | 17.93 | ND | ND |
| ddl | 12.60 | 8.22 | 6.65 | ND | ND |
| def | 11.59 | 9.18 | 5.34 | 6.94 | 4.75 |
| deoB | 16.48 | 13.92 | 6.71 | 7.35 | 6.55 |
| deoC | 14.84 | ND | 5.94 | ND | 8.84 |
| deoD | 13.37 | ND | 6.73 | 5.86 | 6.41 |
| dexA | NA | NA | NA | NA | NA |
| dexB | 24.97 | very stable | very stable | ND | ND |
| dexC | NA | NA | NA | NA | NA |
| dfpA | NA | NA | 3.80 | 6.48 | 4.81 |
| dfpB | 18.22 | 10.22 | 6.32 | ND | 8.62 |
| dfrA | 14.23 | 6.30 | 6.51 | ND | ND |
| dgkA | 13.65 | 10.19 | 4.24 | 9.12 | ND |
| dhaK | 14.82 | 18.84 | 12.62 | 13.72 | ND |
| dhaL | 9.28 | 19.02 | 5.83 | ND | ND |
| dhaM | 13.80 | 19.33 | 27.90 | ND | ND |
| dinF | 14.02 | 8.68 | 3.15 | ND | 4.19 |
| dinG | NA | NA | NA | NA | NA |
| dinP | NA | NA | NA | NA | NA |
| dltB | NA | NA | NA | NA | NA |
| dltC | 13.64 | ND | 4.98 | 6.09 | ND |
| dltD | NA | NA | NA | NA | NA |
| dltE | NA | NA | NA | NA | NA |
| dnaA | NA | NA | NA | NA | NA |
| dnaB | NA | NA | NA | NA | NA |
| dnaC | 10.89 | 12.69 | 4.97 | 7.11 | 6.89 |
| dnaD | 17.19 | 13.32 | 5.82 | ND | ND |
| dnaE | 13.26 | 9.78 | 4.85 | 6.79 | ND |
| dnaG | NA | NA | ND | ND | ND |
| dnaH | ND | ND | 5.19 | 7.45 | ND |
| dnaJ | very stable | very stable | 4.35 | ND | 6.95 |
| dnaK | 18.09 | ND | 5.57 | 8.20 | 7.15 |
| dnaN | 9.51 | 11.61 | NA | NA | NA |
| dnaQ | 13.79 | 7.79 | 4.75 | ND | ND |
| dpsA | 52.53 | ND | 6.89 | 7.93 | 6.34 |
| dtpT | NA | NA | 4.41 | ND | ND |
| dukA | ND | 20.71 | 6.01 | 7.96 | ND |
| dukB | ND | 14.93 | 7.99 | ND | ND |
| dut | 18.20 | 15.97 | 11.30 | 6.86 | ND |
| dxsA | very stable | very stable | ND | ND | ND |

| | | | | | |
|---|---|---|---|---|---|
| dxsB | 13.24 | 11.68 | 5.42 | ND | ND |
| ecsA | NA | NA | NA | NA | NA |
| ecsB | 17.12 | ND | 5.28 | 7.17 | 7.70 |
| efp | 14.12 | 10.05 | 5.19 | 7.29 | 7.33 |
| enoA | 23.89 | 12.05 | 9.40 | 9.10 | ND |
| enoB | 29.33 | ND | 11.71 | ND | 9.59 |
| eraL | 14.66 | 8.00 | 4.28 | 6.22 | 3.63 |
| exoA | 10.87 | 7.15 | 3.88 | 5.61 | 4.96 |
| ezrA | 13.10 | 7.73 | 4.72 | ND | 4.66 |
| fabD | NA | NA | 3.99 | 5.59 | 5.51 |
| fabF | 17.53 | 13.22 | 6.09 | ND | ND |
| fabG1 | 8.92 | 8.70 | 4.88 | ND | ND |
| fabG2 | NA | NA | very stable | ND | ND |
| fabI | 20.64 | 19.59 | 6.44 | ND | 7.24 |
| fabZ1 | 13.90 | 13.89 | 4.38 | ND | 6.82 |
| fabZ2 | 16.93 | ND | 6.54 | 9.47 | 9.83 |
| fadA | NA | NA | NA | NA | NA |
| fadD | 9.48 | 11.87 | 5.13 | ND | 6.85 |
| fbaA | ND | 28.23 | 8.69 | 6.68 | 7.69 |
| fbp | 19.15 | 21.87 | 5.78 | ND | ND |
| femD | ND | ND | NA | NA | NA |
| feoA | 17.15 | 6.92 | NA | NA | NA |
| feoB | 24.12 | 23.85 | 17.08 | ND | ND |
| fer | 12.99 | 7.38 | 4.77 | ND | ND |
| ffh | 8.23 | 7.43 | 5.36 | ND | ND |
| fhs | 29.03 | 9.01 | 4.44 | ND | ND |
| fhuB | ND | 15.07 | 7.27 | ND | ND |
| fhuD | 46.68 | ND | ND | ND | ND |
| fhuG | 39.35 | very stable | ND | ND | ND |
| fhuR | 8.79 | 5.41 | 2.92 | ND | 4.08 |
| floL | NA | NA | 7.52 | ND | ND |
| fmt | 23.28 | very stable | 10.09 | ND | ND |
| folB | 23.58 | 16.33 | 9.13 | ND | ND |
| folC | very stable | ND | 11.76 | ND | ND |
| folD | 14.32 | 9.99 | 4.82 | ND | ND |
| folE | 23.21 | 20.40 | 13.30 | 13.93 | ND |
| folP | 35.27 | 16.92 | 9.02 | 10.86 | 8.94 |
| frdC | 26.03 | very stable | 10.64 | 11.40 | ND |
| frr | 16.75 | 15.74 | 4.61 | ND | 5.06 |
| fruA | NA | NA | 5.72 | ND | ND |
| ftsA | 14.67 | 15.97 | 4.11 | 6.30 | 3.66 |
| ftsE | 29.76 | 34.02 | 11.28 | ND | ND |
| ftsH | 17.56 | 18.66 | 6.19 | 10.24 | 7.04 |
| ftsK | NA | NA | 5.93 | ND | ND |
| ftsQ | NA | NA | 4.55 | ND | ND |
| ftsW1 | 14.31 | 8.63 | 4.00 | 6.76 | 6.13 |
| ftsW2 | 27.02 | 17.05 | 7.09 | ND | ND |
| ftsX | 15.00 | 10.84 | 5.92 | 8.75 | ND |
| ftsY | 13.23 | 14.64 | 6.11 | 7.39 | ND |
| ftsZ | 14.07 | 14.36 | 3.60 | 4.61 | 4.48 |

| | | | | | |
|---|---|---|---|---|---|
| fur | 15.04 | 10.43 | 9.23 | ND | ND |
| fusA | ND | ND | 8.49 | 7.49 | ND |
| gadB | 19.62 | ND | 12.75 | ND | ND |
| gadC | ND | very stable | ND | ND | ND |
| gadR | 71.93 | 23.54 | 9.46 | ND | ND |
| galE | 14.79 | 14.49 | 5.85 | 6.37 | 8.20 |
| galK | 32.37 | ND | NA | NA | NA |
| galM | ND | very stable | ND | ND | ND |
| galT | 28.16 | 19.31 | 7.64 | ND | ND |
| gapA | ND | 20.52 | 12.99 | 9.85 | ND |
| gapB | ND | 18.82 | 11.29 | 7.63 | ND |
| gatB | 13.22 | ND | 5.03 | ND | 7.65 |
| gatC | 12.46 | 11.04 | 4.63 | ND | ND |
| gcp | 10.09 | 12.53 | 4.80 | ND | 5.94 |
| gidA | 17.52 | 13.99 | 6.28 | ND | ND |
| gidB | 9.19 | ND | 4.18 | 5.75 | 5.71 |
| gidC | 12.64 | 5.50 | 3.34 | ND | ND |
| glgA | 21.50 | very stable | 14.88 | ND | ND |
| glgC | 25.81 | 24.85 | 7.32 | ND | ND |
| glgD | 20.36 | ND | 11.93 | 8.85 | ND |
| glgP | 15.94 | ND | 7.47 | ND | ND |
| glk | 25.01 | 18.92 | 7.26 | 7.60 | ND |
| glmS | 24.69 | 10.51 | 6.06 | ND | ND |
| glmU | 13.25 | 13.53 | 4.97 | 4.55 | ND |
| glnA | 12.58 | 7.56 | 6.38 | ND | 6.48 |
| glnB | 36.27 | ND | ND | ND | ND |
| glnP | 15.55 | 12.09 | 6.26 | ND | 6.62 |
| glnQ | NA | NA | ND | ND | ND |
| glnR | 15.88 | 10.49 | 6.26 | 6.34 | 7.00 |
| glpD | very stable | very stable | ND | ND | ND |
| glpF1 | 17.57 | 22.74 | 8.40 | 11.83 | ND |
| glpF2 | very stable | very stable | ND | ND | ND |
| glpK | very stable | ND | very stable | ND | ND |
| glpT | ND | ND | ND | ND | ND |
| gltA | 15.82 | 13.06 | ND | 6.17 | ND |
| gltD | ND | very stable | 8.66 | 7.83 | ND |
| gltQ | very stable | very stable | ND | ND | ND |
| gltS | 55.68 | 36.05 | ND | ND | ND |
| gltX | NA | NA | 4.24 | 5.84 | ND |
| glyA | 12.05 | ND | 4.09 | 5.65 | ND |
| glyS | 27.18 | 20.20 | 6.74 | ND | ND |
| gmk | NA | NA | 6.95 | ND | 7.04 |
| gnd | 20.86 | 18.37 | 5.53 | 7.52 | ND |
| gntK | 13.03 | 10.72 | 3.80 | ND | ND |
| gntR | 9.67 | 7.51 | 3.26 | ND | ND |
| gntZ | 17.09 | 17.82 | 6.76 | ND | ND |
| gpdA | ND | 18.44 | 6.44 | 7.39 | 7.02 |
| gpo | 17.25 | 14.45 | 9.56 | ND | ND |
| greA | 11.63 | ND | 4.48 | ND | ND |
| groEL | NA | NA | NA | NA | NA |

| | | | | | |
|---|---|---|---|---|---|
| groES | 14.20 | 19.51 | 8.13 | 8.18 | ND |
| grpE | 14.66 | 14.01 | 4.69 | 6.33 | ND |
| gshR | 11.05 | 9.82 | 4.05 | ND | ND |
| guaA | 11.97 | 7.72 | 4.81 | 4.24 | ND |
| guaB | 11.99 | 13.02 | 6.59 | 5.61 | ND |
| guaC | 25.42 | 12.99 | ND | ND | ND |
| gyrA | very stable | very stable | very stable | ND | ND |
| gyrB | 24.33 | 19.71 | 19.37 | 7.44 | ND |
| hasC | NA | NA | NA | NA | NA |
| hemH | 27.47 | 9.50 | 7.54 | 7.73 | ND |
| hemK | 20.25 | 12.99 | 7.18 | ND | ND |
| hemN | 14.83 | 7.37 | 3.74 | ND | ND |
| hexA | 14.66 | 10.97 | 5.87 | ND | ND |
| hexB | 13.04 | 11.13 | 5.82 | ND | ND |
| hflX | 14.30 | 12.55 | 5.64 | ND | ND |
| hisA | 56.09 | very stable | very stable | 12.54 | ND |
| hisB | very stable | very stable | very stable | ND | ND |
| hisC | NA | 16.84 | ND | ND | ND |
| hisD | very stable | very stable | ND | ND | ND |
| hisF | NA | NA | NA | NA | NA |
| hisG | very stable | ND | NA | NA | NA |
| hisH | NA | ND | NA | NA | NA |
| hisI | ND | very stable | very stable | ND | ND |
| hisK | ND | very stable | very stable | ND | ND |
| hisS | 20.67 | 13.45 | 7.27 | ND | ND |
| hisZ | 26.31 | ND | very stable | ND | ND |
| hly | 11.75 | 9.32 | 4.27 | ND | 6.09 |
| hmcM | 10.84 | 8.79 | 3.84 | ND | ND |
| holB | 15.92 | 11.15 | 5.89 | ND | 8.72 |
| hom | 6.55 | 6.59 | 4.82 | ND | ND |
| hprT | NA | 48.00 | 5.11 | ND | ND |
| hpt | 13.39 | 9.17 | 6.29 | 6.98 | 6.47 |
| hrcA | 14.90 | 8.54 | 5.25 | ND | ND |
| hsdM | 15.26 | ND | 4.56 | 4.53 | 5.30 |
| hsdR | 31.49 | 22.83 | 18.07 | ND | ND |
| hsdS | 17.54 | ND | 6.92 | 6.39 | ND |
| hslA | very stable | very stable | very stable | ND | ND |
| hslB | 28.56 | very stable | 14.91 | ND | ND |
| htrA | 9.34 | 10.66 | 3.75 | 4.19 | 4.96 |
| icaA | 13.62 | very stable | NA | NA | NA |
| icaB | NA | NA | ND | ND | ND |
| icaC | 17.84 | ND | 6.42 | 5.20 | 8.72 |
| icd | 22.36 | very stable | ND | ND | ND |
| ileS | 21.66 | ND | 8.23 | 5.06 | ND |
| ilvA | NA | NA | NA | NA | NA |
| ilvB | ND | ND | ND | ND | ND |
| ilvC | ND | very stable | very stable | ND | ND |
| ilvD | ND | very stable | ND | ND | ND |
| ilvN | ND | ND | very stable | ND | ND |
| infA | 25.51 | 16.01 | 8.75 | ND | 5.57 |

| | | | | | |
|---|---|---|---|---|---|
| infB | 18.17 | ND | 6.41 | 5.01 | ND |
| infC | 20.63 | 19.00 | 8.17 | 7.56 | ND |
| ipd | 22.95 | very stable | ND | ND | ND |
| ispA | 17.80 | 10.92 | 3.19 | ND | ND |
| ispB | 9.99 | 8.81 | 3.84 | ND | 6.56 |
| kdgA | very stable | 28.07 | ND | ND | ND |
| kdgK | very stable | ND | very stable | ND | ND |
| kdgR | NA | NA | 9.10 | ND | ND |
| kdtB | 14.34 | 8.36 | 10.74 | ND | ND |
| kinA | 20.60 | 19.04 | 7.98 | ND | ND |
| kinB | 12.87 | 7.69 | 3.77 | ND | 5.17 |
| kinC | very stable | very stable | 15.63 | 9.69 | ND |
| kinD | 26.17 | 30.05 | 10.57 | 6.83 | ND |
| kinE | ND | very stable | 18.03 | ND | ND |
| kinF | 15.89 | 14.20 | 5.21 | ND | ND |
| kinG | NA | NA | 6.23 | ND | ND |
| ksgA | very stable | 14.25 | 9.64 | ND | ND |
| kupA | 11.52 | ND | 3.96 | ND | ND |
| kupB | 33.32 | 15.81 | 4.77 | ND | 5.86 |
| lacC | very stable | 15.57 | very stable | ND | ND |
| lacR | ND | 18.20 | 8.59 | ND | ND |
| lacZ | very stable | very stable | ND | ND | ND |
| lcnC | ND | 16.09 | 15.15 | ND | ND |
| lcnD | 16.85 | 10.36 | 4.32 | ND | ND |
| lctO | 76.12 | 21.51 | ND | 10.63 | ND |
| ldh | ND | very stable | 15.34 | 6.38 | ND |
| ldhB | very stable | very stable | very stable | ND | ND |
| ldhX | 27.97 | 22.32 | ND | ND | ND |
| lepA | 18.15 | 14.73 | 9.81 | ND | ND |
| leuB | 31.67 | very stable | ND | 6.40 | ND |
| leuC | ND | ND | very stable | 6.65 | ND |
| leuD | ND | ND | ND | 7.30 | ND |
| leuS | 12.19 | 11.49 | 6.94 | ND | ND |
| lgt | ND | ND | 5.72 | ND | ND |
| ligA | 55.98 | ND | 14.80 | ND | ND |
| llrA | 16.80 | ND | 6.07 | ND | ND |
| llrB | 31.72 | 6.16 | 4.72 | ND | ND |
| llrC | 11.73 | 8.03 | 6.31 | 4.68 | ND |
| llrD | 17.40 | 17.70 | 8.59 | ND | ND |
| llrE | 51.52 | very stable | ND | ND | ND |
| llrF | 10.33 | 8.59 | 5.84 | ND | ND |
| llrG | 18.88 | 12.28 | 8.76 | ND | ND |
| llrH | very stable | very stable | very stable | ND | ND |
| lmrA | ND | very stable | 12.88 | ND | ND |
| lmrP | NA | NA | 18.68 | ND | ND |
| lnbA | 15.36 | 14.26 | very stable | ND | ND |
| lplL | 60.80 | ND | ND | ND | ND |
| lspA | 8.59 | 4.91 | 4.35 | ND | ND |
| lysA | 12.47 | very stable | ND | ND | ND |
| lysP | 14.24 | 9.09 | 1.98 | ND | ND |

| | | | | | |
|---|---|---|---|---|---|
| lysQ | 13.02 | 13.79 | 3.44 | 4.45 | 4.72 |
| lysS | 10.73 | 7.60 | 4.87 | 7.79 | 5.04 |
| maa | NA | NA | NA | NA | NA |
| mae | 10.90 | 9.21 | 3.89 | ND | 6.41 |
| malE | NA | NA | NA | NA | NA |
| malF | NA | NA | NA | NA | NA |
| malG | NA | NA | NA | NA | NA |
| malQ | 23.91 | very stable | very stable | ND | ND |
| mapA | NA | NA | NA | NA | NA |
| menB | 15.06 | ND | 5.69 | ND | ND |
| menD | ND | 15.65 | 10.17 | ND | ND |
| menE | 15.02 | ND | ND | ND | ND |
| menF | 41.29 | 6.03 | 3.63 | ND | ND |
| menX | 17.99 | 15.93 | ND | ND | ND |
| mesJ | 10.87 | 10.66 | 4.22 | ND | ND |
| metA | NA | NA | NA | NA | NA |
| metB1 | very stable | ND | 6.59 | ND | ND |
| metB2 | 19.51 | ND | NA | NA | NA |
| metE | very stable | very stable | ND | ND | ND |
| metF | NA | NA | NA | NA | NA |
| metK | 24.56 | ND | 9.76 | 7.64 | ND |
| metS | 15.22 | 12.72 | ND | ND | ND |
| mfd | NA | NA | NA | NA | NA |
| mgtA | 60.71 | 26.57 | very stable | ND | ND |
| miaA | 13.16 | 12.73 | 4.14 | ND | 6.29 |
| mleP | NA | NA | ND | ND | ND |
| mleR | 16.42 | 9.50 | NA | NA | NA |
| mleS | 12.93 | 11.59 | 6.11 | 7.70 | ND |
| mraY | NA | NA | very stable | ND | ND |
| mreC | 20.67 | 11.60 | 7.97 | 9.03 | 11.87 |
| mreD | 11.58 | 10.22 | 4.42 | 4.85 | ND |
| mscL | 15.80 | 13.57 | 5.66 | 6.96 | ND |
| msmK | 12.07 | 40.54 | 5.00 | ND | ND |
| mtlD | ND | very stable | ND | ND | ND |
| mtlF | NA | NA | NA | NA | NA |
| mtlR | NA | NA | NA | NA | NA |
| mtsA | 11.02 | very stable | 3.94 | 4.52 | 6.25 |
| mtsB | 11.55 | 9.19 | 4.12 | 6.07 | 4.70 |
| mtsC | 10.49 | ND | 3.62 | 4.67 | 5.95 |
| murA1 | 29.24 | 13.97 | 6.16 | ND | 7.27 |
| murA2 | 16.54 | 10.73 | 4.51 | ND | 5.39 |
| murB | 15.03 | 14.79 | 5.71 | ND | ND |
| murC | 20.72 | ND | 6.81 | ND | ND |
| murD | 24.25 | 10.49 | 6.44 | ND | ND |
| murE | 21.11 | 13.36 | 7.20 | ND | ND |
| murF | 12.87 | 10.28 | 3.83 | ND | ND |
| murG | NA | NA | 4.19 | ND | ND |
| murI | 20.48 | 36.03 | 9.42 | 12.70 | ND |
| mutM | 15.61 | ND | 4.98 | 4.98 | ND |
| mutS | 11.20 | 15.28 | 4.09 | ND | ND |

| | | | | | |
|---|---|---|---|---|---|
| mutX | ND | very stable | 12.19 | ND | ND |
| mutY | NA | NA | 7.00 | ND | ND |
| mvaA | 18.17 | 10.93 | 5.57 | ND | ND |
| mycA | 63.74 | very stable | NA | NA | NA |
| nadE | 11.62 | 17.11 | 6.66 | ND | 6.44 |
| nadR | 10.92 | 16.76 | 5.08 | ND | ND |
| nagA | 18.05 | ND | 6.69 | 8.94 | 6.89 |
| nagB | 9.67 | ND | 3.24 | ND | ND |
| nah | 10.86 | 13.79 | 4.96 | ND | ND |
| napB | ND | very stable | ND | ND | ND |
| napC | 13.06 | 27.77 | NA | NA | NA |
| ndrH | 24.20 | 14.60 | NA | NA | NA |
| ndrI | 32.91 | ND | ND | ND | ND |
| nifJ | NA | NA | 5.20 | ND | ND |
| nifS | 13.57 | 12.89 | 5.11 | ND | ND |
| nifU | 20.42 | ND | ND | ND | ND |
| nifZ | 19.57 | 10.77 | 4.15 | ND | ND |
| noxA | 20.45 | 10.25 | 4.65 | ND | ND |
| noxB | 12.37 | 5.75 | 3.55 | 6.21 | 5.52 |
| noxC | 28.69 | ND | ND | ND | ND |
| noxD | 18.42 | very stable | ND | ND | ND |
| noxE | 9.38 | very stable | 7.92 | ND | ND |
| nrdD | 23.94 | 41.89 | 8.90 | ND | ND |
| nrdE | 23.98 | very stable | ND | 8.47 | ND |
| nrdF | 23.25 | ND | ND | ND | ND |
| nrdG | 19.86 | ND | 9.17 | ND | ND |
| nth | 13.44 | 9.14 | 4.12 | ND | ND |
| nucA | 21.01 | 36.78 | very stable | ND | ND |
| nusA | 21.40 | 10.43 | 4.93 | ND | ND |
| nusB | 11.48 | 9.58 | 6.06 | ND | ND |
| nusG | 10.43 | 11.00 | 5.62 | ND | 7.54 |
| obgL | very stable | very stable | very stable | ND | ND |
| ogt | 22.42 | ND | ND | ND | ND |
| oppA | NA | ND | NA | NA | NA |
| oppB | ND | ND | very stable | ND | ND |
| oppC | ND | ND | ND | ND | ND |
| oppD | 40.88 | ND | ND | ND | ND |
| oppF | NA | ND | NA | NA | NA |
| optA | 8.43 | 6.52 | 4.20 | 4.77 | 5.14 |
| optB | 10.79 | 13.21 | 7.03 | 6.58 | ND |
| optC | 11.79 | ND | 5.98 | 7.77 | ND |
| optD | 12.32 | very stable | 7.61 | 6.14 | ND |
| optF | ND | very stable | 8.58 | ND | ND |
| optS | 16.17 | 8.15 | ND | 5.36 | ND |
| osmC | 23.57 | 47.17 | 25.33 | ND | ND |
| otcA | very stable | ND | very stable | ND | ND |
| pabA | NA | NA | NA | NA | NA |
| pabB | 26.26 | ND | 9.61 | ND | ND |
| pacA | NA | NA | NA | NA | NA |
| pacB | 13.74 | 24.46 | 5.76 | 8.54 | 6.18 |

| | | | | | |
|---|---|---|---|---|---|
| pacL | very stable | very stable | very stable | ND | ND |
| panE | 22.11 | 8.39 | 4.19 | ND | 5.13 |
| papL | 15.76 | 11.67 | 5.07 | ND | ND |
| parA | 11.77 | ND | ND | ND | ND |
| parC | 25.24 | 11.97 | 7.01 | 12.85 | ND |
| parE | 16.00 | 8.01 | 5.12 | ND | ND |
| pbp1B | 13.38 | 9.15 | 5.15 | ND | ND |
| pbp2A | 19.16 | ND | 6.70 | 6.82 | ND |
| pbp2B | 48.97 | 41.72 | 10.44 | ND | ND |
| pbpX | 11.57 | 14.54 | 5.74 | ND | ND |
| pbuX | NA | ND | 3.75 | ND | ND |
| pcaC | 25.54 | 14.93 | ND | ND | ND |
| pcrA | 11.73 | 11.72 | 4.08 | ND | ND |
| pdc | NA | ND | NA | NA | NA |
| pdhA | 12.90 | ND | 5.21 | ND | ND |
| pdhB | 19.22 | very stable | ND | 9.16 | ND |
| pdhC | 15.68 | very stable | NA | NA | NA |
| pdhD | 16.17 | ND | 11.34 | 9.58 | ND |
| pdp | 17.22 | 15.96 | 5.31 | ND | ND |
| pepA | NA | NA | ND | ND | ND |
| pepC | 18.75 | very stable | ND | 5.78 | ND |
| pepDA | 23.55 | 22.94 | 12.21 | 6.98 | ND |
| pepDB | 16.56 | very stable | 7.45 | ND | ND |
| pepF | 12.12 | 20.47 | 5.22 | ND | ND |
| pepM | 13.19 | 8.46 | 3.92 | ND | ND |
| pepN | 9.18 | 9.49 | 4.31 | 6.44 | 5.21 |
| pepO | 15.25 | 21.24 | 5.96 | 5.89 | ND |
| pepP | 11.16 | 10.54 | 7.50 | ND | ND |
| pepQ | NA | NA | 12.08 | ND | ND |
| pepT | very stable | very stable | very stable | ND | ND |
| pepV | 15.00 | 14.76 | 11.54 | ND | ND |
| pepXP | 13.41 | 21.24 | 7.19 | 5.62 | ND |
| pfk | NA | NA | NA | NA | NA |
| pfl | 8.72 | 11.93 | 4.11 | ND | ND |
| pflA | 13.13 | 10.15 | 3.05 | ND | 5.25 |
| pfs | 15.54 | ND | 8.51 | ND | ND |
| pgiA | NA | NA | 4.25 | ND | ND |
| pgk | 15.64 | 17.42 | 5.45 | 5.74 | ND |
| pgmB | 25.30 | 29.88 | ND | ND | ND |
| pgsA | 11.37 | 11.17 | 5.89 | 6.84 | ND |
| pheA | 15.07 | ND | ND | 8.52 | ND |
| pheS | 15.41 | 9.90 | very stable | ND | ND |
| pheT | 16.10 | ND | 6.07 | ND | 7.28 |
| phnA | 63.04 | 22.65 | ND | 12.22 | ND |
| phnB | very stable | ND | very stable | ND | ND |
| phnC | NA | NA | 7.59 | ND | ND |
| phnE | very stable | very stable | ND | ND | ND |
| phoL | 9.68 | 6.87 | 2.16 | ND | ND |
| phoU | 25.41 | very stable | ND | ND | ND |
| pi101 | 13.87 | 13.29 | ND | ND | ND |

| | | | | | |
|---|---|---|---|---|---|
| pi102 | 14.34 | ND | 7.66 | 7.59 | 7.94 |
| pi103 | 22.06 | 16.32 | 9.52 | 12.58 | ND |
| pi104 | very stable | very stable | ND | ND | ND |
| pi105 | ND | very stable | NA | NA | NA |
| pi106 | 44.54 | 17.76 | NA | NA | NA |
| pi107 | 38.13 | ND | very stable | ND | ND |
| pi108 | very stable | very stable | NA | NA | NA |
| pi109 | NA | NA | NA | NA | NA |
| pi110 | very stable | very stable | ND | ND | ND |
| pi111 | very stable | ND | NA | NA | NA |
| pi113 | ND | ND | very stable | ND | ND |
| pi114 | very stable | ND | very stable | ND | ND |
| pi115 | very stable | ND | very stable | ND | ND |
| pi116 | very stable | ND | very stable | ND | ND |
| pi117 | 36.81 | very stable | NA | NA | NA |
| pi118 | ND | ND | NA | NA | NA |
| pi119 | NA | NA | very stable | ND | ND |
| pi120 | 58.16 | very stable | ND | ND | ND |
| pi122 | 40.52 | ND | ND | ND | ND |
| pi123 | very stable | ND | ND | ND | ND |
| pi124 | very stable | very stable | ND | ND | ND |
| pi125 | 37.16 | very stable | 12.82 | ND | ND |
| pi127 | 27.43 | 23.89 | 7.03 | ND | ND |
| pi128 | 52.04 | very stable | ND | ND | ND |
| pi129 | very stable | very stable | very stable | ND | ND |
| pi130 | very stable | very stable | NA | NA | NA |
| pi132 | NA | NA | very stable | ND | ND |
| pi133 | ND | very stable | very stable | ND | ND |
| pi135 | very stable | very stable | very stable | ND | ND |
| pi137 | very stable | very stable | very stable | ND | ND |
| pi138 | NA | NA | NA | NA | NA |
| pi139 | very stable | very stable | very stable | ND | ND |
| pi140 | very stable | ND | very stable | ND | ND |
| pi141 | very stable | ND | very stable | ND | ND |
| pi142 | ND | ND | very stable | ND | ND |
| pi143 | NA | NA | NA | NA | NA |
| pi144 | 50.59 | very stable | ND | ND | ND |
| pi145 | 12.32 | ND | ND | ND | ND |
| pi147 | NA | 12.26 | ND | ND | ND |
| pi201 | NA | NA | 5.68 | 7.27 | ND |
| pi202 | 27.11 | ND | 5.79 | ND | ND |
| pi203 | NA | NA | 4.86 | ND | ND |
| pi204 | NA | NA | ND | ND | ND |
| pi205 | ND | ND | NA | NA | NA |
| pi207 | NA | NA | NA | NA | NA |
| pi208 | very stable | very stable | ND | ND | ND |
| pi209 | NA | ND | NA | NA | NA |
| pi210 | NA | NA | NA | NA | NA |
| pi211 | 17.81 | ND | 8.24 | ND | 7.22 |
| pi213 | ND | very stable | very stable | ND | ND |

| | | | | | |
|---|---|---|---|---|---|
| pi215 | NA | NA | NA | NA | NA |
| pi216 | NA | NA | NA | NA | NA |
| pi217 | ND | very stable | very stable | ND | ND |
| pi218 | very stable | very stable | very stable | ND | ND |
| pi222 | ND | ND | ND | ND | ND |
| pi223 | very stable | ND | 7.51 | ND | ND |
| pi224 | very stable | 24.64 | 25.50 | ND | ND |
| pi225 | NA | very stable | NA | NA | NA |
| pi226 | 23.33 | 15.86 | ND | ND | ND |
| pi227 | very stable | ND | ND | ND | ND |
| pi228 | very stable | very stable | very stable | ND | ND |
| pi229 | NA | NA | NA | NA | NA |
| pi230 | very stable | ND | very stable | ND | ND |
| pi231 | very stable | ND | ND | ND | ND |
| pi232 | very stable | very stable | NA | NA | NA |
| pi233 | ND | ND | very stable | ND | ND |
| pi234 | very stable | very stable | very stable | ND | ND |
| pi235 | ND | 49.19 | NA | NA | NA |
| pi236 | very stable | very stable | very stable | ND | ND |
| pi237 | ND | very stable | ND | ND | ND |
| pi238 | 24.30 | ND | very stable | ND | ND |
| pi239 | ND | ND | very stable | ND | ND |
| pi240 | NA | NA | ND | ND | ND |
| pi241 | 54.44 | very stable | NA | NA | NA |
| pi242 | very stable | very stable | ND | ND | ND |
| pi243 | very stable | very stable | very stable | ND | ND |
| pi244 | ND | very stable | very stable | ND | ND |
| pi245 | very stable | ND | very stable | ND | ND |
| pi246 | very stable | ND | very stable | ND | ND |
| pi247 | very stable | very stable | very stable | ND | ND |
| pi248 | NA | very stable | ND | ND | ND |
| pi249 | ND | very stable | NA | NA | NA |
| pi251 | 38.45 | 15.81 | ND | ND | ND |
| pi301 | NA | very stable | NA | NA | NA |
| pi302 | very stable | very stable | NA | NA | NA |
| pi303 | ND | very stable | very stable | ND | ND |
| pi307 | ND | very stable | NA | NA | NA |
| pi308 | very stable | very stable | NA | NA | NA |
| pi310 | NA | NA | NA | NA | NA |
| pi316 | ND | very stable | very stable | ND | ND |
| pi317 | very stable | very stable | very stable | ND | ND |
| pi318 | very stable | very stable | very stable | ND | ND |
| pi319 | very stable | ND | very stable | ND | ND |
| pi320 | very stable | very stable | very stable | ND | ND |
| pi321 | ND | very stable | NA | NA | NA |
| pi322 | very stable | ND | ND | ND | ND |
| pi323 | very stable | ND | ND | ND | ND |
| pi324 | very stable | very stable | very stable | ND | ND |
| pi325 | ND | ND | NA | NA | NA |
| pi326 | very stable | very stable | ND | ND | ND |

| | | | | | |
|---|---|---|---|---|---|
| pi327 | very stable | NA | very stable | ND | ND |
| pi328 | 48.05 | very stable | very stable | ND | ND |
| pi329 | ND | very stable | very stable | ND | ND |
| pi330 | very stable | ND | very stable | ND | ND |
| pi331 | very stable | ND | very stable | ND | ND |
| pi333 | very stable | very stable | 22.44 | ND | ND |
| pi334 | very stable | ND | ND | ND | ND |
| pi336 | ND | ND | NA | NA | NA |
| pi337 | very stable | 35.56 | ND | ND | ND |
| pi338 | very stable | very stable | very stable | ND | ND |
| pi339 | very stable | very stable | ND | ND | ND |
| pi341 | NA | NA | ND | ND | ND |
| pi343 | 23.16 | very stable | very stable | ND | ND |
| pi345 | very stable | very stable | very stable | ND | ND |
| pi347 | NA | very stable | very stable | ND | ND |
| pi348 | 31.06 | 26.08 | very stable | ND | ND |
| pi349 | very stable | ND | ND | ND | ND |
| pi350 | very stable | very stable | very stable | ND | ND |
| pi353 | 43.38 | ND | NA | NA | NA |
| pi354 | 24.97 | very stable | very stable | ND | ND |
| pi355 | 31.15 | ND | 11.43 | ND | ND |
| pi356 | very stable | very stable | very stable | ND | ND |
| pi357 | 37.31 | 39.12 | NA | NA | NA |
| pi358 | 16.71 | 17.21 | 6.92 | 10.70 | ND |
| pi359 | 22.90 | 27.76 | ND | ND | ND |
| pi360 | 12.73 | 11.95 | NA | NA | NA |
| pip | 12.71 | 9.63 | 6.65 | 6.69 | ND |
| pknB | 17.10 | 13.49 | 4.37 | ND | 5.76 |
| plpA | NA | very stable | 6.03 | ND | ND |
| plpB | NA | ND | 4.81 | ND | ND |
| plpC | NA | very stable | 5.75 | ND | ND |
| plpD | NA | ND | 5.67 | ND | ND |
| plsX | 15.65 | 13.72 | 5.92 | ND | ND |
| pmg | 14.15 | 12.02 | 5.21 | 5.68 | ND |
| pmi | NA | NA | 4.86 | 6.47 | 5.95 |
| pmpA | 10.12 | 8.31 | 3.75 | 4.15 | 4.67 |
| pmrA | 16.59 | 11.79 | 6.55 | 7.61 | ND |
| pmrB | NA | NA | 8.76 | ND | ND |
| pmsR | 24.02 | 15.89 | 6.83 | 7.95 | 9.20 |
| pmsX | 15.22 | 22.07 | 7.13 | ND | ND |
| pnpA | 18.34 | 7.23 | 3.69 | ND | ND |
| pnuC2 | very stable | 29.84 | very stable | ND | ND |
| polA | 18.14 | 9.22 | 7.18 | ND | ND |
| polC | 24.76 | very stable | 12.46 | 8.14 | ND |
| ponA | 12.44 | 11.88 | 3.69 | 3.68 | ND |
| potA | 13.69 | 12.05 | 4.72 | 4.35 | 5.73 |
| potB | ND | ND | 5.45 | 4.71 | 6.79 |
| potC | 16.13 | ND | 5.40 | 5.19 | ND |
| potD | 20.81 | very stable | 7.19 | 7.41 | ND |
| poxL | very stable | very stable | very stable | ND | ND |

| | | | | | |
|---|---|---|---|---|---|
| ppiA | 9.86 | 6.83 | 3.33 | ND | 5.53 |
| ppiB | ND | very stable | 11.29 | ND | ND |
| preA | 32.55 | 9.41 | 3.60 | ND | ND |
| prfA | very stable | 33.22 | 24.20 | ND | ND |
| prfB | 21.64 | 12.80 | 6.72 | ND | ND |
| prfC | 21.54 | 15.22 | 8.54 | ND | ND |
| priA | NA | NA | NA | NA | NA |
| prmA | 16.01 | ND | 5.91 | 6.86 | 8.35 |
| proA | 29.18 | very stable | 8.87 | ND | ND |
| proB | 18.26 | 13.41 | 3.74 | ND | ND |
| proC | 13.51 | 16.61 | 4.86 | 9.56 | ND |
| proS | 16.00 | very stable | 7.40 | 6.70 | ND |
| prsA | 20.15 | 12.02 | 4.46 | 6.10 | 4.50 |
| prsB | 15.97 | 9.28 | 4.30 | ND | 4.88 |
| ps101 | very stable | very stable | very stable | ND | ND |
| ps102 | very stable | ND | very stable | 15.06 | ND |
| ps103 | NA | NA | NA | NA | NA |
| ps104 | NA | ND | ND | ND | ND |
| ps105 | ND | very stable | very stable | ND | ND |
| ps106 | very stable | very stable | ND | ND | ND |
| ps107 | very stable | very stable | ND | ND | ND |
| ps108 | ND | ND | very stable | ND | ND |
| ps109 | very stable | very stable | very stable | ND | ND |
| ps110 | very stable | very stable | 22.33 | ND | ND |
| ps111 | NA | NA | ND | ND | ND |
| ps112 | very stable | very stable | ND | ND | ND |
| ps113 | 35.69 | ND | NA | NA | NA |
| ps114 | very stable | very stable | ND | ND | ND |
| ps115 | NA | 12.61 | 18.99 | ND | ND |
| ps116 | very stable | 22.32 | 7.44 | ND | 8.52 |
| ps117 | 12.03 | 10.17 | NA | NA | NA |
| ps118 | 14.34 | 11.02 | NA | NA | NA |
| ps119 | 20.59 | ND | 6.80 | ND | ND |
| ps120 | 16.73 | 9.75 | NA | NA | NA |
| ps121 | 9.42 | 9.58 | ND | ND | ND |
| ps122 | 40.73 | very stable | very stable | ND | ND |
| ps123 | 16.11 | ND | 8.54 | ND | ND |
| ps201 | ND | 15.62 | 11.50 | ND | ND |
| ps202 | 18.25 | 19.57 | 6.74 | ND | ND |
| ps203 | 11.45 | 12.86 | 4.84 | ND | ND |
| ps205 | ND | 41.79 | 25.44 | ND | ND |
| ps206 | 49.25 | very stable | very stable | ND | ND |
| ps207 | very stable | ND | very stable | ND | ND |
| ps209 | very stable | very stable | ND | ND | ND |
| ps211 | NA | very stable | NA | NA | NA |
| ps212 | very stable | very stable | ND | ND | ND |
| ps213 | ND | very stable | ND | ND | ND |
| ps214 | very stable | very stable | very stable | ND | ND |
| ps215 | NA | NA | very stable | ND | ND |
| ps216 | very stable | very stable | very stable | ND | ND |

| | | | | | |
|---|---|---|---|---|---|
| ps218 | ND | very stable | very stable | ND | ND |
| ps219 | ND | ND | very stable | ND | ND |
| ps220 | very stable | very stable | ND | ND | ND |
| ps221 | NA | NA | 14.95 | ND | ND |
| ps301 | 19.63 | 9.51 | 4.67 | ND | ND |
| ps302 | 13.81 | 8.23 | 3.91 | 5.67 | 4.82 |
| ps303 | 11.96 | 6.34 | 3.58 | 5.43 | 4.98 |
| ps304 | very stable | ND | very stable | ND | ND |
| ps305 | 37.28 | 12.29 | 7.93 | ND | ND |
| ps306 | ND | very stable | ND | ND | ND |
| ps307 | NA | very stable | ND | ND | ND |
| ps308 | 29.05 | ND | very stable | ND | ND |
| ps309 | NA | NA | NA | NA | NA |
| ps310 | very stable | ND | 11.09 | ND | ND |
| ps311 | NA | very stable | very stable | ND | ND |
| ps312 | very stable | NA | NA | NA | NA |
| ps313 | NA | NA | NA | NA | NA |
| ps314 | NA | NA | NA | NA | NA |
| ps315 | 12.68 | 8.51 | 3.69 | ND | ND |
| ps316 | 12.81 | 7.54 | 4.52 | ND | ND |
| pspB | 13.97 | 7.47 | 5.50 | 7.59 | 6.40 |
| pstA | 26.26 | very stable | 6.66 | 6.21 | ND |
| pstB | 16.51 | ND | 6.15 | ND | ND |
| pstC | 16.71 | ND | 6.39 | ND | ND |
| pstE | 27.40 | 24.94 | NA | NA | NA |
| pstF | 42.02 | ND | ND | ND | ND |
| pta | 15.72 | 20.23 | 5.27 | 8.02 | ND |
| ptbA | 18.62 | ND | 4.91 | ND | ND |
| ptcA | NA | ND | 4.93 | ND | ND |
| ptcB | 13.12 | 11.26 | 3.78 | ND | ND |
| ptcC | very stable | ND | ND | ND | ND |
| pth | NA | NA | 5.32 | ND | ND |
| ptk | 12.54 | ND | ND | ND | ND |
| ptnAB | 23.11 | 41.35 | very stable | ND | ND |
| ptnC | 21.52 | ND | very stable | ND | ND |
| ptnD | ND | ND | very stable | 10.25 | ND |
| ptpL | ND | 8.19 | NA | NA | NA |
| ptsH | 18.67 | 17.80 | 6.32 | 6.46 | ND |
| ptsI | 20.31 | 18.44 | 5.39 | 5.30 | ND |
| ptsK | 15.49 | 10.83 | 4.28 | ND | ND |
| purA | NA | NA | 6.39 | ND | 5.16 |
| purB | 12.22 | 7.40 | 3.65 | ND | ND |
| purC | ND | very stable | 6.85 | 12.10 | ND |
| purD | 40.56 | 12.68 | 3.98 | 6.79 | ND |
| purE | 35.57 | 17.24 | 5.32 | ND | ND |
| purF | 75.80 | 13.55 | ND | 6.96 | ND |
| purH | ND | very stable | 10.36 | 12.49 | ND |
| purK | ND | very stable | 5.33 | ND | ND |
| purL | very stable | ND | 6.73 | ND | ND |
| purM | very stable | 33.05 | 11.76 | ND | ND |

| | | | | | |
|---|---|---|---|---|---|
| purN | very stable | 16.83 | 12.07 | ND | ND |
| purQ | NA | NA | NA | NA | NA |
| purR | 11.74 | 14.14 | 7.39 | ND | ND |
| pycA | 11.30 | 15.21 | 4.45 | ND | 6.69 |
| pydA | 22.62 | 12.03 | 9.27 | 8.06 | ND |
| pydB | 43.97 | 11.33 | 3.69 | ND | ND |
| pyk | 23.04 | ND | ND | 7.54 | ND |
| pyrB | 19.39 | 23.22 | 4.94 | 7.61 | ND |
| pyrC | 17.45 | 13.80 | 4.48 | 5.62 | ND |
| pyrE | 40.17 | 14.91 | 5.96 | ND | 6.75 |
| pyrF | ND | 22.52 | very stable | ND | ND |
| pyrG | 32.74 | 14.51 | NA | NA | NA |
| pyrH | 29.19 | 14.77 | 9.81 | ND | ND |
| pyrP | NA | NA | 5.96 | ND | ND |
| pyrR | NA | 10.48 | 7.38 | ND | ND |
| pyrZ | 46.53 | ND | 5.75 | ND | ND |
| qor | 29.40 | ND | NA | NA | NA |
| queA | 17.21 | 13.36 | 8.06 | ND | ND |
| racD | 17.79 | ND | 9.60 | 7.34 | ND |
| radA | 20.10 | 13.77 | 10.75 | ND | ND |
| radC | 61.94 | ND | 15.88 | ND | ND |
| rarA | 26.11 | 19.30 | 13.40 | ND | ND |
| rbfA | very stable | very stable | NA | NA | NA |
| rbsA | ND | 17.82 | 12.87 | 10.20 | ND |
| rbsB | ND | ND | very stable | ND | ND |
| rbsC | 25.87 | 39.56 | very stable | ND | ND |
| rbsD | ND | ND | ND | ND | ND |
| rbsK | 17.25 | 16.95 | 3.79 | ND | ND |
| rbsR | 22.78 | ND | 9.69 | ND | ND |
| rcfA | ND | ND | very stable | ND | ND |
| rcfB | 15.08 | 9.65 | 5.43 | ND | ND |
| rdrA | 21.77 | 13.66 | ND | ND | ND |
| rdrB | 6.55 | 7.10 | 4.59 | ND | ND |
| recA | 13.43 | 13.68 | 7.00 | 5.14 | ND |
| recD | ND | very stable | 7.08 | 10.85 | ND |
| recJ | 22.94 | 12.88 | 9.13 | ND | ND |
| recM | ND | very stable | very stable | ND | ND |
| recN | 21.98 | ND | 6.26 | ND | ND |
| recQ | ND | very stable | 18.67 | ND | ND |
| relA | 11.53 | 10.21 | 4.10 | 5.48 | 6.24 |
| rexA | 23.55 | very stable | 9.98 | ND | ND |
| rexB | ND | very stable | very stable | ND | ND |
| rgpA | 15.63 | 18.52 | 6.79 | 10.17 | 6.44 |
| rgpB | 12.14 | 14.80 | 5.92 | 7.20 | 6.06 |
| rgpC | 11.00 | 19.56 | 5.96 | 7.04 | ND |
| rgpE | 30.17 | very stable | ND | ND | ND |
| rgpF | 12.76 | ND | ND | ND | ND |
| rgrA | 17.60 | ND | 9.10 | ND | ND |
| rgrB | 14.18 | 11.89 | 7.64 | ND | ND |
| rheA | very stable | very stable | very stable | ND | ND |

| | | | | | |
|---|---|---|---|---|---|
| rheB | 15.99 | 12.79 | 4.55 | 7.49 | 4.85 |
| ribA | 24.39 | very stable | very stable | ND | ND |
| ribC | 23.11 | 7.70 | 5.38 | ND | ND |
| ribG | NA | NA | NA | NA | NA |
| ribH | very stable | ND | NA | NA | NA |
| rimM | 33.06 | 27.77 | 8.83 | ND | ND |
| rliA | 22.81 | 17.56 | 7.04 | 8.38 | ND |
| rliB | 10.25 | 6.39 | 2.82 | 3.45 | 3.10 |
| rliC | 24.62 | 13.23 | 7.34 | ND | ND |
| rliDB | ND | ND | very stable | ND | ND |
| rlrA | 12.74 | 8.76 | 5.37 | ND | ND |
| rlrB | NA | 14.51 | 5.03 | ND | ND |
| rlrC | 12.80 | 15.37 | 4.50 | ND | ND |
| rlrD | 11.15 | 9.13 | 4.22 | ND | ND |
| rlrG | 18.18 | ND | ND | ND | ND |
| rluA | 13.19 | 12.64 | 6.96 | ND | ND |
| rluB | ND | ND | 14.18 | ND | ND |
| rluC | 13.05 | 10.24 | 8.37 | ND | ND |
| rluD | 14.49 | 7.59 | 3.28 | ND | ND |
| rmaA | very stable | 16.37 | very stable | ND | ND |
| rmaB | 27.48 | very stable | ND | ND | ND |
| rmaC | 18.53 | 15.47 | 9.22 | ND | ND |
| rmaD | 24.92 | 15.20 | 4.41 | ND | ND |
| rmaE | 39.26 | very stable | very stable | ND | ND |
| rmaF | 28.45 | 14.26 | 15.91 | ND | ND |
| rmaG | 13.05 | 8.66 | 2.99 | ND | 3.87 |
| rmaH | very stable | very stable | very stable | ND | ND |
| rmaI | 49.97 | very stable | very stable | ND | ND |
| rmaJ | 18.32 | 9.30 | 5.74 | ND | ND |
| rmeA | very stable | very stable | very stable | ND | ND |
| rmeB | 11.54 | 5.99 | 3.38 | ND | ND |
| rmeC | very stable | very stable | very stable | ND | ND |
| rmeD | ND | very stable | NA | NA | NA |
| rmlA | ND | ND | 6.07 | ND | ND |
| rmlB | 12.80 | ND | 7.16 | 7.88 | ND |
| rmlC | 28.87 | very stable | ND | ND | ND |
| rnc | ND | very stable | very stable | ND | ND |
| rnhA | 25.21 | very stable | 7.18 | 9.45 | ND |
| rnhB | 23.44 | ND | ND | ND | ND |
| rnpA | ND | ND | 17.40 | ND | ND |
| rodA | 22.74 | 9.48 | NA | NA | NA |
| rpe | very stable | very stable | 9.16 | ND | 12.39 |
| rpiA | ND | 18.42 | 7.29 | 10.32 | 8.81 |
| rplA | 30.43 | 16.09 | 7.70 | 9.25 | ND |
| rplB | 47.83 | ND | ND | ND | ND |
| rplC | NA | NA | ND | ND | ND |
| rplE | NA | NA | NA | NA | NA |
| rplF | NA | NA | ND | 11.83 | ND |
| rplI | 11.51 | ND | 4.65 | 5.65 | ND |
| rplJ | NA | NA | ND | ND | ND |

| | | | | | |
|---|---|---|---|---|---|
| rplK | NA | NA | ND | 8.46 | ND |
| rplL | NA | NA | very stable | ND | ND |
| rplM | 23.39 | 7.44 | 5.21 | 7.67 | ND |
| rplN | very stable | 25.76 | very stable | ND | ND |
| rplO | 38.26 | 13.37 | 6.76 | ND | ND |
| rplQ | 21.27 | 14.51 | 6.69 | ND | 4.65 |
| rplR | ND | ND | 10.01 | 13.84 | ND |
| rplS | 26.22 | ND | 7.19 | 10.34 | ND |
| rplT | 19.36 | ND | 8.11 | 8.97 | ND |
| rplU | 22.50 | ND | ND | 9.29 | ND |
| rplV | ND | very stable | ND | ND | ND |
| rplW | NA | NA | NA | NA | NA |
| rplX | ND | ND | very stable | ND | ND |
| rpmA | 44.89 | very stable | ND | ND | ND |
| rpmB | 24.92 | 27.16 | 7.54 | ND | ND |
| rpmC | NA | NA | NA | NA | NA |
| rpmD | very stable | very stable | ND | ND | ND |
| rpmE | 26.90 | 13.68 | 9.21 | 8.18 | ND |
| rpmF | 19.20 | 10.43 | 6.96 | 10.13 | 7.90 |
| rpmGA | ND | 9.83 | NA | NA | NA |
| rpmGB | 26.05 | 10.42 | 6.92 | ND | ND |
| rpmGC | 13.35 | 12.45 | NA | NA | NA |
| rpmH | very stable | very stable | very stable | ND | ND |
| rpmI | 16.56 | 12.33 | 6.20 | 9.02 | ND |
| rpmJ | 21.14 | 14.10 | 7.60 | 13.26 | 5.21 |
| rpoA | 22.42 | 16.47 | 8.32 | ND | ND |
| rpoB | 32.45 | 14.57 | ND | 13.48 | ND |
| rpoC | 21.77 | 26.23 | 10.75 | ND | ND |
| rpoD | 36.64 | very stable | ND | ND | ND |
| rpoE | 13.47 | 8.39 | 4.05 | 6.19 | 4.12 |
| rpsA | 25.83 | 16.65 | 6.27 | 8.15 | ND |
| rpsB | 16.52 | 16.96 | 4.94 | 6.71 | 5.09 |
| rpsC | 40.25 | ND | ND | ND | ND |
| rpsD | 28.79 | 12.01 | 4.76 | 9.09 | 5.18 |
| rpsE | very stable | ND | ND | ND | ND |
| rpsF | 20.54 | 23.51 | 8.86 | 8.62 | ND |
| rpsG | 20.26 | 16.78 | 7.44 | 15.65 | ND |
| rpsH | 26.12 | 29.22 | 8.79 | ND | ND |
| rpsI | 14.72 | 10.51 | ND | 6.19 | ND |
| rpsJ | 28.28 | 26.98 | ND | ND | ND |
| rpsK | 21.37 | 12.00 | 6.30 | ND | 5.80 |
| rpsL | 17.13 | 6.53 | 4.99 | 9.17 | ND |
| rpsM | 19.95 | 17.39 | 7.67 | 9.94 | 5.02 |
| rpsN | very stable | ND | very stable | 9.67 | ND |
| rpsN2 | 20.22 | ND | ND | ND | ND |
| rpsO | 44.50 | ND | 6.47 | 7.92 | ND |
| rpsP | 12.03 | 7.89 | 4.19 | 5.75 | 5.23 |
| rpsQ | ND | very stable | very stable | ND | ND |
| rpsR | 21.46 | 25.17 | ND | ND | ND |
| rpsS | 37.65 | 29.21 | NA | NA | NA |

| | | | | | |
|---|---|---|---|---|---|
| rpsT | 33.71 | 15.25 | 7.32 | ND | ND |
| rpsU | 32.55 | 12.05 | 7.00 | ND | ND |
| rsuA | 8.99 | 5.86 | 4.56 | ND | 5.67 |
| ruvA | 11.19 | 14.18 | 4.96 | ND | ND |
| ruvB | 12.87 | ND | 4.74 | 6.40 | ND |
| sbcC | ND | very stable | very stable | ND | ND |
| sbcD | 30.32 | 26.42 | 9.60 | ND | ND |
| scrK | very stable | very stable | very stable | ND | ND |
| sdaA | 43.88 | very stable | 8.19 | ND | ND |
| sdaB | 13.66 | 6.72 | 4.82 | ND | ND |
| secA | 11.78 | 5.79 | 3.26 | ND | ND |
| secE | 42.31 | 17.45 | very stable | ND | ND |
| secG | 41.13 | 14.74 | NA | NA | NA |
| secY | 14.75 | 11.50 | 21.21 | ND | ND |
| serA | 14.52 | 9.04 | 4.07 | 5.84 | ND |
| serB | 23.00 | ND | 5.99 | 8.49 | ND |
| serC | 11.66 | 6.20 | 4.51 | 5.65 | ND |
| serS | 20.04 | ND | 8.41 | ND | ND |
| sigX | NA | 21.93 | very stable | ND | ND |
| sipL | 13.84 | 14.99 | 7.85 | 6.82 | ND |
| smc | 19.43 | ND | 11.28 | 14.56 | ND |
| smpB | 18.11 | 11.74 | 6.55 | ND | ND |
| snf | 14.82 | 9.15 | 4.49 | ND | 4.91 |
| sodA | 11.55 | 9.77 | 4.09 | 4.46 | 3.97 |
| ssbA | NA | NA | NA | NA | NA |
| ssbB | 28.39 | 24.57 | 8.35 | ND | ND |
| sugE | 15.69 | NA | NA | NA | NA |
| sunL | 14.74 | 9.88 | 4.63 | ND | ND |
| tag | 17.00 | ND | 12.77 | ND | ND |
| tagB | 31.11 | 30.71 | very stable | ND | ND |
| tagD1 | 48.08 | ND | ND | ND | ND |
| tagD2 | very stable | ND | very stable | ND | ND |
| tagF | NA | very stable | NA | NA | NA |
| tagH | very stable | very stable | ND | ND | ND |
| tagL | NA | NA | NA | NA | NA |
| tagR | NA | ND | ND | ND | ND |
| tagX | very stable | ND | very stable | ND | ND |
| tagY | NA | NA | NA | NA | NA |
| tagZ | NA | 27.57 | NA | NA | NA |
| tenA | 18.64 | ND | ND | ND | ND |
| tgt | NA | NA | 5.44 | 7.95 | ND |
| thdF | 13.95 | 7.54 | 4.89 | ND | ND |
| thgA | ND | ND | very stable | ND | ND |
| thiD1 | 37.20 | ND | 9.13 | ND | ND |
| thiD2 | 11.93 | 11.09 | 4.39 | ND | ND |
| thiE | very stable | ND | very stable | ND | ND |
| thiL | 27.76 | 11.51 | 4.99 | ND | ND |
| thiM | 48.09 | 31.54 | 9.76 | ND | ND |
| thrA | 9.37 | 6.81 | 3.48 | 4.28 | ND |
| thrB | 33.95 | ND | 8.97 | 7.19 | ND |

| | | | | | |
|---|---|---|---|---|---|
| thrC | 11.99 | 7.54 | 4.93 | 5.26 | 6.70 |
| thrS | 20.08 | 13.45 | ND | 13.38 | ND |
| thyA | 13.50 | 7.25 | 4.73 | 5.77 | ND |
| tig | 14.05 | 12.91 | 4.57 | ND | ND |
| tkt | 10.31 | 9.42 | 4.54 | 6.04 | ND |
| topA | 13.15 | 10.60 | 3.43 | ND | ND |
| tpiA | 25.18 | very stable | 7.38 | 10.68 | ND |
| tpx | 18.61 | ND | 9.55 | ND | ND |
| tra904A | 21.62 | ND | 5.88 | 12.70 | 5.71 |
| tra905 | very stable | ND | very stable | ND | ND |
| tra981C | 32.18 | ND | 10.29 | ND | ND |
| tra983L | 32.73 | ND | 6.88 | 7.65 | 8.91 |
| tra1077B | 24.23 | 21.03 | 6.56 | 9.45 | ND |
| trmD | 12.68 | 7.48 | 5.17 | ND | ND |
| trmH | NA | NA | very stable | ND | ND |
| trmU | 31.54 | 25.06 | 13.43 | ND | ND |
| trpA | ND | ND | ND | ND | ND |
| trpB | 23.11 | very stable | 7.24 | ND | ND |
| trpC | ND | very stable | ND | ND | ND |
| trpD | ND | ND | 9.50 | ND | ND |
| trpE | ND | very stable | 8.69 | ND | ND |
| trpF | very stable | ND | 17.60 | ND | ND |
| trpG | 31.79 | 7.15 | 3.45 | ND | ND |
| trpS | 12.97 | 8.45 | 4.21 | ND | ND |
| truA | 10.67 | 5.60 | 5.03 | ND | ND |
| truB | 20.92 | 11.14 | 4.26 | ND | ND |
| trxA | 18.64 | 13.91 | 5.33 | 6.22 | ND |
| trxB1 | 10.52 | 12.96 | 4.78 | ND | ND |
| trxB2 | 16.79 | 19.92 | 4.69 | ND | ND |
| trxH | 10.12 | 9.30 | 3.11 | 4.12 | 3.75 |
| tsf | 20.03 | very stable | 6.26 | 7.08 | ND |
| tuf | 33.30 | 39.77 | ND | ND | ND |
| typA | 13.90 | 7.74 | 3.80 | 4.75 | 3.77 |
| tyrA | 13.01 | 11.87 | 5.52 | ND | 8.90 |
| tyrS | 53.95 | ND | 14.81 | ND | 7.37 |
| udk | 13.05 | 8.42 | 2.97 | ND | 5.34 |
| udp | 13.63 | 8.06 | 3.48 | ND | ND |
| umuC | 22.09 | very stable | NA | NA | NA |
| ung | 19.55 | 12.82 | 8.21 | ND | ND |
| upp | 17.34 | 15.81 | 7.63 | ND | ND |
| usp45 | 12.16 | 7.67 | 4.29 | 5.05 | 5.03 |
| uvrA | 12.03 | 11.44 | 4.62 | ND | 4.76 |
| uvrB | 22.35 | 44.50 | 10.42 | ND | ND |
| uvrC | 72.03 | 24.44 | 10.42 | ND | ND |
| uxaC | ND | ND | 11.35 | ND | ND |
| uxuA | NA | NA | 6.62 | ND | ND |
| uxuB | NA | ND | ND | ND | ND |
| uxuT | 25.73 | ND | 9.70 | ND | ND |
| vacB1 | 15.89 | 11.62 | 9.72 | ND | ND |
| vacB2 | 20.18 | 10.47 | ND | ND | ND |

| | | | | | |
|---|---|---|---|---|---|
| valS | 9.80 | 9.49 | 4.67 | ND | 7.58 |
| xerD | ND | very stable | very stable | ND | ND |
| xpt | NA | 14.47 | NA | NA | NA |
| xseA | 26.35 | 34.04 | 6.14 | 8.95 | 6.68 |
| xylA | NA | NA | ND | ND | ND |
| xylB | NA | NA | NA | NA | NA |
| xylH | 45.86 | ND | NA | NA | NA |
| xylM | ND | ND | very stable | ND | ND |
| xylR | 16.48 | 11.67 | 6.63 | ND | 7.65 |
| xylT | 39.00 | very stable | very stable | ND | ND |
| xylX | 14.78 | ND | ND | ND | ND |
| xynB | ND | ND | NA | NA | NA |
| xynD | 16.46 | 18.34 | 6.01 | 5.15 | ND |
| xynT | 32.51 | ND | 27.03 | ND | ND |
| yabA | NA | NA | ND | ND | ND |
| yabB | 60.47 | very stable | ND | ND | ND |
| yabC | 23.35 | very stable | very stable | ND | ND |
| yabD | 52.98 | very stable | ND | ND | ND |
| yabE | very stable | ND | very stable | ND | ND |
| yabF | 18.77 | 16.17 | 8.81 | ND | ND |
| yacB | 12.86 | 6.47 | 3.77 | ND | 3.54 |
| yacC | 11.88 | 8.06 | 3.93 | ND | 4.20 |
| yacG | 9.53 | 9.15 | 4.53 | 7.73 | ND |
| yacI | 13.87 | 11.49 | 5.30 | ND | ND |
| yafB | very stable | very stable | ND | ND | ND |
| yafC | 61.45 | 13.53 | 9.05 | ND | ND |
| yafD | 12.70 | 14.97 | 6.12 | ND | ND |
| yafE | 29.66 | very stable | ND | ND | ND |
| yafF | 24.18 | very stable | very stable | ND | ND |
| yafJ | ND | very stable | ND | ND | ND |
| yagA | 16.30 | 17.85 | 4.76 | ND | ND |
| yagB | 24.92 | ND | 6.12 | 10.50 | 8.37 |
| yagE | 20.47 | 12.55 | 6.78 | ND | ND |
| yahA | 18.11 | 15.94 | 5.63 | ND | 8.86 |
| yahB | 26.89 | 44.41 | 7.87 | 8.56 | ND |
| yahC | ND | 22.83 | ND | ND | ND |
| yahD | ND | very stable | 15.73 | ND | ND |
| yahG | 19.78 | 9.03 | 5.31 | 8.15 | 5.59 |
| yahI | 15.81 | 12.74 | 7.94 | 8.39 | 10.24 |
| yaiA | 18.84 | ND | 20.92 | ND | ND |
| yaiB | 25.22 | very stable | 7.65 | ND | ND |
| yaiE | 20.03 | 19.39 | 11.80 | ND | ND |
| yaiF | 48.99 | 47.20 | 18.16 | ND | ND |
| yaiG | very stable | very stable | very stable | ND | ND |
| yaiH | NA | NA | 13.21 | 11.25 | 10.21 |
| yaiI | very stable | very stable | ND | ND | ND |
| yajB | 26.35 | 25.76 | very stable | ND | ND |
| yajE | 21.98 | ND | 13.95 | ND | ND |
| yajF | NA | NA | NA | NA | NA |
| yajH | 18.58 | 18.82 | very stable | ND | ND |

| | | | | | |
|---|---|---|---|---|---|
| ybaA | very stable | very stable | very stable | ND | ND |
| ybaB | 25.91 | 8.09 | 7.08 | ND | ND |
| ybaC | 30.78 | 13.94 | 8.66 | ND | 8.45 |
| ybaD | 9.56 | 8.05 | 3.49 | ND | ND |
| ybaF | 14.29 | ND | 6.04 | ND | ND |
| ybaG | 12.67 | ND | 6.21 | 6.75 | ND |
| ybaH | 14.61 | 6.34 | 5.52 | ND | ND |
| ybaI | 18.86 | 11.76 | 4.73 | ND | ND |
| ybbA | very stable | very stable | very stable | ND | ND |
| ybbB | 20.88 | ND | 9.77 | ND | ND |
| ybbC | 13.36 | 9.90 | 3.10 | ND | ND |
| ybbE | 46.92 | 17.50 | 10.67 | 13.19 | ND |
| ybcC | ND | 36.10 | ND | ND | ND |
| ybcG | very stable | very stable | very stable | ND | ND |
| ybcH | 20.02 | 13.08 | 4.50 | ND | ND |
| ybdA | 15.13 | 9.81 | 5.03 | ND | ND |
| ybdC | 15.93 | 10.75 | 5.95 | 6.72 | ND |
| ybdD | 13.87 | 13.33 | 5.58 | 7.61 | ND |
| ybdG | NA | NA | NA | NA | NA |
| ybdH | 43.15 | 21.66 | 11.82 | ND | ND |
| ybdI | very stable | ND | very stable | ND | ND |
| ybdJ | 24.67 | 18.33 | NA | NA | NA |
| ybdK | 18.96 | 14.92 | 5.19 | ND | ND |
| ybdL | 24.69 | 20.19 | 4.88 | 6.85 | ND |
| ybeA | 17.01 | ND | 6.19 | 7.33 | ND |
| ybeB | ND | very stable | ND | ND | ND |
| ybeC | 40.38 | ND | 7.39 | ND | ND |
| ybeD | 19.60 | 21.87 | ND | ND | ND |
| ybeF | ND | very stable | 8.55 | ND | ND |
| ybeH | 15.55 | 10.41 | 6.94 | ND | ND |
| ybeI | 25.85 | ND | very stable | ND | ND |
| ybeM | 16.43 | 11.06 | ND | ND | ND |
| ybfA | 21.56 | 10.00 | 7.16 | 5.66 | ND |
| ybfB | 11.47 | 8.25 | NA | NA | NA |
| ybfC | 18.94 | 12.96 | ND | ND | ND |
| ybfD | 24.42 | very stable | ND | ND | ND |
| ybfE | 14.50 | ND | 6.57 | ND | ND |
| ybgA | 31.72 | 14.29 | 7.50 | ND | ND |
| ybgB | NA | NA | 5.22 | 7.26 | 4.63 |
| ybgD | 44.74 | ND | 9.47 | 7.82 | ND |
| ybgE | 32.96 | very stable | ND | ND | ND |
| ybhA | 25.59 | 23.29 | 7.16 | 9.79 | ND |
| ybhB | 13.32 | 10.63 | 3.83 | ND | ND |
| ybhC | very stable | ND | 7.70 | 11.79 | ND |
| ybhD | 7.74 | NA | 3.79 | ND | 6.76 |
| ybhE | very stable | very stable | 17.92 | ND | ND |
| ybiB | 41.02 | 13.94 | 8.65 | ND | ND |
| ybiC | 20.17 | 11.42 | 7.40 | ND | 6.11 |
| ybiD | 12.63 | 8.38 | 3.54 | ND | 3.39 |
| ybiE | 9.59 | 7.03 | 3.89 | 5.43 | 4.42 |

| | | | | | |
|---|---|---|---|---|---|
| ybiG | 24.29 | 16.12 | 4.54 | ND | ND |
| ybiH | 10.87 | ND | 3.94 | 4.07 | ND |
| ybiI | 14.90 | ND | NA | NA | NA |
| ybiJ | 16.27 | ND | 13.21 | ND | ND |
| ybiK | 33.63 | very stable | very stable | ND | ND |
| ybjA | 12.58 | 15.45 | 2.95 | ND | ND |
| ybjB | 14.66 | 15.20 | 7.26 | ND | ND |
| ybjD | 36.87 | 16.25 | 10.52 | ND | 11.66 |
| ybjJ | 17.45 | 12.56 | 5.77 | 9.54 | 7.02 |
| ybjK | 22.51 | 12.10 | 8.09 | 11.26 | ND |
| ycaF | 17.76 | ND | 6.64 | 6.57 | ND |
| ycaG | 12.58 | ND | 8.58 | ND | ND |
| ycbA | 22.50 | ND | very stable | 9.44 | ND |
| ycbB | 32.06 | very stable | very stable | ND | ND |
| ycbC | ND | ND | very stable | ND | ND |
| ycbD | ND | ND | very stable | ND | ND |
| ycbF | very stable | ND | ND | ND | ND |
| ycbH | ND | ND | ND | ND | ND |
| ycbI | very stable | ND | ND | ND | ND |
| ycbJ | very stable | ND | very stable | ND | ND |
| yccB | 24.42 | 18.54 | 13.70 | ND | ND |
| yccE | 26.38 | 12.32 | 10.77 | ND | ND |
| yccF | 24.21 | very stable | 7.06 | ND | ND |
| yccG | 20.02 | ND | 6.46 | ND | ND |
| yccH | 16.59 | ND | 7.69 | 7.10 | ND |
| yccI | 25.46 | ND | 15.05 | 12.24 | ND |
| yccJ | ND | very stable | 9.30 | ND | ND |
| yccK | ND | 32.25 | 12.49 | 11.78 | ND |
| yccL | very stable | very stable | ND | ND | ND |
| ycdA | 11.28 | 11.41 | 4.87 | ND | ND |
| ycdB | 19.61 | 10.13 | 3.59 | 5.78 | 4.18 |
| ycdC | very stable | very stable | very stable | ND | ND |
| ycdE | NA | ND | ND | ND | ND |
| ycdF | NA | ND | 5.60 | ND | ND |
| ycdG | 34.08 | ND | very stable | ND | ND |
| ycdH | very stable | ND | 18.08 | ND | ND |
| yceA | very stable | very stable | very stable | ND | ND |
| yceD | 49.10 | 16.59 | ND | ND | ND |
| yceE | 29.04 | 13.31 | 10.41 | ND | ND |
| yceG | NA | NA | 3.79 | ND | ND |
| yceJ | 8.66 | NA | NA | NA | NA |
| ycfA | 35.11 | 15.62 | 7.21 | ND | ND |
| ycfB | 21.28 | 9.26 | 3.78 | ND | ND |
| ycfC | NA | NA | NA | NA | NA |
| ycfD | 17.25 | 8.98 | 4.97 | ND | 5.92 |
| ycfF | 13.47 | 7.89 | 3.79 | 5.78 | ND |
| ycfG | very stable | 30.63 | 12.59 | ND | ND |
| ycfH | very stable | very stable | very stable | ND | ND |
| ycfI | NA | very stable | very stable | ND | ND |
| ycgA | NA | ND | 10.14 | ND | ND |

| | | | | | |
|---|---|---|---|---|---|
| ycgB | ND | very stable | ND | 11.61 | ND |
| ycgC | 12.70 | 14.33 | 7.23 | 6.92 | ND |
| ycgD | very stable | very stable | 27.06 | 8.51 | ND |
| ycgE | 22.25 | 26.68 | 9.01 | 7.56 | ND |
| ycgF | 11.95 | 14.71 | 3.92 | 4.87 | ND |
| ycgG | 14.01 | 13.19 | 3.65 | 4.26 | ND |
| ycgH | 20.03 | 16.14 | 8.59 | ND | ND |
| ycgI | 14.39 | 10.48 | 4.75 | 5.68 | ND |
| ycgJ | 16.90 | 11.71 | 12.27 | ND | ND |
| ychC | 14.07 | 9.35 | 4.94 | ND | ND |
| ychD | very stable | 29.19 | 9.76 | ND | 7.62 |
| ychE | very stable | very stable | 11.05 | ND | 8.67 |
| ychG | 20.08 | 13.30 | ND | ND | ND |
| ychH | NA | NA | 5.69 | 5.44 | ND |
| yciA | 13.47 | ND | 4.83 | 4.96 | ND |
| yciC | 15.59 | 7.62 | 4.70 | ND | ND |
| yciD | very stable | very stable | very stable | ND | ND |
| yciF | ND | very stable | NA | NA | NA |
| yciG | ND | ND | 19.74 | ND | ND |
| yciH | NA | NA | 4.71 | 5.72 | 6.04 |
| ycjA | 12.48 | 6.15 | 3.93 | ND | ND |
| ycjB | 14.86 | 15.68 | 4.65 | ND | 6.18 |
| ycjC | 11.35 | 9.41 | 3.75 | ND | ND |
| ycjD | 10.21 | 10.05 | 6.69 | ND | ND |
| ycjG | NA | NA | NA | NA | NA |
| ycjH | 53.99 | 30.70 | 8.88 | ND | 7.82 |
| ycjI | 16.15 | 9.25 | 6.02 | ND | ND |
| ycjM | NA | NA | 6.92 | ND | ND |
| ydaE | 10.97 | 6.43 | very stable | ND | ND |
| ydaF | very stable | very stable | ND | ND | ND |
| ydaG | 28.28 | 18.17 | 9.91 | 4.80 | ND |
| ydbA | 26.51 | ND | 20.64 | 5.25 | ND |
| ydbC | 23.15 | 14.85 | NA | NA | NA |
| ydbD | 8.56 | 6.66 | 4.33 | ND | 5.63 |
| ydbE | 20.84 | 9.07 | 4.46 | 5.55 | 5.60 |
| ydbF | 16.91 | 11.15 | 13.90 | ND | ND |
| ydbH | NA | NA | 9.52 | ND | ND |
| ydcB | 46.85 | 13.65 | 4.76 | ND | ND |
| ydcD | very stable | very stable | 11.06 | ND | ND |
| ydcE | 38.17 | 24.98 | ND | ND | ND |
| ydcF | 25.33 | 17.80 | 6.89 | 8.02 | ND |
| ydcG | 17.14 | 10.28 | ND | ND | ND |
| yddA | very stable | ND | very stable | ND | ND |
| yddB | ND | ND | ND | ND | ND |
| yddC | ND | 24.22 | very stable | 8.19 | ND |
| yddD | 34.26 | 29.48 | very stable | 9.72 | ND |
| ydgB | 11.78 | ND | 5.22 | 4.88 | 6.80 |
| ydgC | 12.01 | 10.42 | 4.66 | 5.97 | 4.67 |
| ydgD | 16.15 | 9.12 | 6.65 | 7.21 | ND |
| ydgE | 22.80 | 9.77 | 9.45 | 7.39 | ND |

| | | | | | |
|---|---|---|---|---|---|
| ydgF | 14.51 | 6.44 | 4.39 | ND | ND |
| ydgG | 16.05 | 6.56 | 4.01 | ND | ND |
| ydgH | 15.86 | 14.52 | NA | NA | NA |
| ydgI | 18.14 | 8.82 | 6.93 | ND | ND |
| ydgJ | NA | NA | 6.29 | ND | ND |
| ydhB | very stable | very stable | very stable | ND | ND |
| ydhF | ND | very stable | ND | ND | ND |
| ydiA | ND | ND | 9.55 | ND | ND |
| ydiB | very stable | 10.30 | ND | ND | ND |
| ydiC | 28.83 | 12.36 | ND | ND | ND |
| ydiD | ND | very stable | very stable | ND | ND |
| ydiE | very stable | 36.57 | ND | ND | ND |
| ydiF | 30.04 | 17.02 | very stable | ND | ND |
| ydiG | ND | NA | NA | NA | NA |
| ydjB | 12.28 | 7.70 | 3.68 | 5.14 | 4.92 |
| ydjD | 18.03 | 15.60 | ND | ND | ND |
| yeaA | 16.33 | 12.49 | very stable | ND | ND |
| yeaB | NA | NA | NA | NA | NA |
| yeaC | 27.26 | ND | 6.33 | ND | ND |
| yeaD | 36.45 | 14.97 | ND | ND | ND |
| yeaE | NA | NA | NA | NA | NA |
| yeaF | very stable | very stable | NA | NA | NA |
| yeaG | 15.97 | 13.98 | 3.32 | ND | ND |
| yeaH | 17.15 | ND | 4.88 | ND | ND |
| yebA | NA | NA | 8.20 | ND | 9.45 |
| yebB | 21.75 | ND | 4.81 | ND | ND |
| yebE | very stable | 18.35 | 5.47 | 5.63 | ND |
| yebF | 11.88 | 26.86 | 7.13 | 9.69 | 8.69 |
| yecA | 20.42 | 29.76 | 4.68 | ND | ND |
| yecD | 18.11 | 14.18 | NA | NA | NA |
| yecE | 14.28 | ND | very stable | ND | ND |
| yedA | 40.25 | ND | 4.30 | ND | ND |
| yedE | 10.11 | 10.22 | 4.40 | ND | ND |
| yedF | 12.00 | 12.08 | 10.68 | ND | ND |
| yeeA | 31.78 | very stable | NA | NA | NA |
| yeeB | very stable | very stable | NA | NA | NA |
| yeeC | 42.61 | ND | 4.61 | 6.64 | 5.89 |
| yeeD | NA | NA | NA | NA | NA |
| yeeE | 14.80 | 6.59 | 4.52 | ND | ND |
| yeeF | 42.28 | ND | NA | NA | NA |
| yeeG | NA | NA | 3.95 | 5.36 | 3.78 |
| yeiD | 22.26 | ND | 5.09 | ND | ND |
| yeiE | 10.57 | 10.51 | 5.13 | ND | ND |
| yeiF | 10.88 | very stable | ND | ND | ND |
| yeiG | 27.35 | ND | ND | ND | ND |
| yejC | 20.61 | 10.44 | 4.14 | ND | ND |
| yejD | ND | very stable | NA | NA | NA |
| yejE | 27.34 | ND | very stable | ND | ND |
| yejH | 25.74 | 15.71 | NA | NA | NA |
| yejI | 28.04 | 30.29 | 7.13 | 9.57 | ND |

| | | | | | |
|---|---|---|---|---|---|
| yejJ | 71.94 | very stable | very stable | ND | ND |
| yfaA | 25.25 | 21.68 | ND | ND | ND |
| yfbB | NA | ND | NA | NA | NA |
| yfbG | 12.22 | 9.87 | 4.59 | ND | ND |
| yfbI | 30.56 | ND | 4.54 | 6.37 | ND |
| yfbJ | very stable | ND | 4.73 | 5.44 | ND |
| yfbK | 14.32 | very stable | NA | NA | NA |
| yfbM | 16.14 | ND | ND | ND | ND |
| yfcA | NA | NA | very stable | ND | ND |
| yfcB | 15.38 | ND | NA | NA | NA |
| yfcC | NA | ND | NA | NA | NA |
| yfcD | NA | NA | NA | NA | NA |
| yfcF | 42.39 | ND | 6.98 | ND | ND |
| yfcG | 11.28 | 14.21 | NA | NA | NA |
| yfcI | 10.62 | 8.62 | NA | NA | NA |
| yfdA | 16.02 | 48.14 | 2.72 | ND | ND |
| yfdB | 70.72 | 15.27 | very stable | ND | ND |
| yfdC | NA | NA | 7.68 | ND | 7.13 |
| yfdD | ND | very stable | very stable | ND | ND |
| yfdE | very stable | very stable | 5.56 | ND | 5.98 |
| yfeA | 30.14 | 9.39 | ND | ND | ND |
| yffA | very stable | ND | 11.82 | ND | ND |
| yffB | 14.37 | 14.31 | very stable | ND | ND |
| yffD | ND | 32.78 | NA | NA | NA |
| yfgC | NA | 8.09 | very stable | ND | ND |
| yfgF | 28.26 | 14.85 | NA | NA | NA |
| yfgG | 38.07 | very stable | ND | ND | ND |
| yfgH | 12.01 | 7.63 | 7.66 | 6.88 | ND |
| yfgL | 26.94 | very stable | 3.87 | ND | ND |
| yfgQ | NA | NA | 8.49 | 9.52 | ND |
| yfhA | 17.06 | 14.97 | very stable | ND | ND |
| yfhB | 19.05 | 13.50 | 5.05 | ND | ND |
| yfhC | NA | NA | NA | NA | NA |
| yfhF | NA | NA | NA | NA | NA |
| yfhG | 29.39 | 44.70 | NA | NA | NA |
| yfhH | 16.75 | 13.97 | 6.15 | 7.56 | ND |
| yfhI | 11.19 | 8.26 | very stable | ND | ND |
| yfhJ | 12.65 | 8.84 | ND | ND | ND |
| yfhK | 15.76 | ND | 9.81 | ND | ND |
| yfhL | 25.18 | 19.51 | ND | ND | ND |
| yfiA | NA | NA | very stable | ND | ND |
| yfiC | 16.98 | 13.59 | 7.54 | 11.05 | ND |
| yfiD | NA | NA | 8.06 | ND | 7.99 |
| yfiE | 19.25 | 20.50 | 9.24 | 9.82 | ND |
| yfiG | ND | ND | very stable | ND | ND |
| yfiH | 16.82 | 8.18 | 10.81 | ND | ND |
| yfiJ | 15.72 | ND | 7.57 | ND | ND |
| yfiL | 25.63 | 19.56 | NA | NA | NA |
| yfjA | 14.38 | 10.45 | ND | ND | ND |
| yfjB | 10.49 | 6.07 | NA | NA | NA |

| | | | | | |
|---|---|---|---|---|---|
| yfjC | 12.03 | 15.31 | 16.64 | ND | ND |
| yfjD | 17.07 | 18.53 | NA | NA | NA |
| yfjE | NA | NA | ND | ND | ND |
| yfjF | 13.92 | 20.08 | NA | NA | NA |
| yfjG | NA | NA | very stable | ND | ND |
| yfjH | NA | NA | very stable | ND | ND |
| ygaB | 11.82 | ND | 6.84 | 9.04 | 8.64 |
| ygaC | 10.49 | 12.90 | NA | NA | NA |
| ygaD | 12.26 | 7.75 | very stable | ND | ND |
| ygaE | ND | 31.35 | ND | ND | ND |
| ygaF | 55.75 | 9.69 | 8.52 | ND | ND |
| ygaI | 20.22 | ND | 7.51 | ND | ND |
| ygaJ | 13.74 | 22.70 | 5.26 | 7.05 | ND |
| ygbB | 15.21 | ND | 8.95 | ND | ND |
| ygbD | 10.12 | 15.46 | 9.68 | 12.29 | 7.37 |
| ygbE | 13.92 | 13.34 | 5.09 | 5.34 | ND |
| ygbF | 27.94 | very stable | 6.46 | 10.17 | ND |
| ygbG | 26.05 | very stable | 15.10 | ND | ND |
| ygcA | 11.06 | 7.66 | 9.93 | ND | ND |
| ygcC | 26.81 | 13.19 | 6.84 | ND | 6.54 |
| ygdA | NA | NA | NA | NA | NA |
| ygdC | 37.86 | ND | 8.33 | 10.57 | ND |
| ygdD | 30.15 | very stable | ND | ND | ND |
| ygdE | 11.15 | 8.85 | very stable | ND | ND |
| ygdF | NA | NA | NA | NA | NA |
| ygeA | 14.26 | 10.85 | 6.55 | ND | ND |
| ygeB | 19.99 | very stable | ND | ND | ND |
| ygeC | very stable | ND | NA | NA | NA |
| ygeD | 16.37 | 10.81 | 10.42 | ND | ND |
| ygfA | ND | ND | very stable | ND | ND |
| ygfB | ND | ND | NA | NA | NA |
| ygfC | NA | 10.73 | 10.80 | ND | ND |
| ygfE | 18.64 | 16.82 | ND | ND | ND |
| yggA | 14.52 | 10.34 | 5.87 | ND | ND |
| yghB | 23.10 | very stable | 19.98 | 8.81 | ND |
| yghC | 31.68 | 9.61 | 9.17 | 8.65 | ND |
| yghD | 26.09 | ND | 15.68 | ND | ND |
| yghE | 18.14 | 8.34 | NA | NA | NA |
| yghG | 11.87 | ND | NA | NA | NA |
| ygiC | 13.92 | ND | 7.49 | ND | ND |
| ygiG | ND | ND | very stable | ND | ND |
| ygiH | NA | NA | very stable | ND | ND |
| ygiI | 38.09 | 24.44 | ND | ND | ND |
| ygiJ | 17.05 | 6.97 | NA | NA | NA |
| ygiK | 14.77 | 10.87 | 6.37 | ND | ND |
| ygjB | 14.48 | 10.54 | 8.83 | ND | ND |
| ygjD | 11.54 | NA | ND | ND | ND |
| yhbE | 35.90 | very stable | very stable | ND | ND |
| yhbF | NA | NA | NA | NA | NA |
| yhbH | 28.60 | 26.89 | 20.73 | ND | ND |

| | | | | | |
|---|---|---|---|---|---|
| yhcA | 17.39 | 17.13 | 7.52 | 10.70 | ND |
| yhcB | 18.56 | 11.11 | 7.99 | ND | ND |
| yhcC | 29.16 | 18.01 | ND | ND | ND |
| yhcE | 25.67 | ND | 5.43 | ND | ND |
| yhcG | NA | NA | NA | NA | NA |
| yhcH | ND | ND | 15.69 | ND | ND |
| yhcI | ND | ND | 4.43 | ND | ND |
| yhcK | 43.46 | ND | NA | NA | NA |
| yhdA | NA | ND | very stable | ND | ND |
| yhdB | 26.78 | ND | 9.74 | ND | ND |
| yhdC | 10.77 | NA | NA | NA | NA |
| yheA | 17.70 | 14.72 | 7.84 | ND | 10.01 |
| yheB | 16.44 | ND | 6.72 | 11.10 | ND |
| yheD | NA | NA | 4.15 | ND | ND |
| yheE | NA | NA | 5.11 | 8.55 | ND |
| yheG | NA | NA | very stable | ND | ND |
| yhfA | NA | NA | 4.43 | ND | ND |
| yhfB | 10.92 | 9.58 | 3.55 | ND | ND |
| yhfC | 11.15 | 7.01 | 4.12 | 6.02 | 5.53 |
| yhfD | 11.97 | 12.81 | 6.83 | ND | ND |
| yhfE | 12.49 | 14.22 | 6.70 | 10.73 | 8.45 |
| yhfF | 9.85 | 8.32 | 6.03 | 7.83 | ND |
| yhgA | 15.18 | 15.93 | ND | 9.38 | ND |
| yhgB | 14.56 | ND | ND | ND | ND |
| yhgC | 24.32 | ND | 7.98 | ND | ND |
| yhgD | very stable | ND | very stable | ND | ND |
| yhgE | 67.46 | very stable | very stable | ND | ND |
| yhhA | 37.90 | very stable | very stable | ND | ND |
| yhhB | very stable | very stable | NA | NA | NA |
| yhhC | NA | NA | 4.46 | ND | ND |
| yhhD | 29.67 | 10.42 | 7.95 | ND | ND |
| yhhE | 24.16 | ND | 5.47 | ND | ND |
| yhhG | 9.93 | 11.45 | 4.77 | ND | ND |
| yhjA | 23.30 | ND | 14.23 | 9.66 | ND |
| yhjB | NA | NA | NA | NA | NA |
| yhjC | 26.45 | 12.67 | 8.08 | ND | ND |
| yhjE | ND | 56.58 | very stable | ND | ND |
| yhjF | 33.42 | ND | 16.88 | ND | ND |
| yhjG | ND | ND | very stable | ND | ND |
| yiaA | ND | very stable | 14.12 | ND | ND |
| yiaB | 21.21 | very stable | 11.08 | ND | ND |
| yiaC | 60.84 | 16.30 | 5.98 | ND | ND |
| yiaD | 12.26 | 17.26 | 5.06 | ND | 6.97 |
| yibB | 17.43 | ND | 5.90 | ND | ND |
| yibC | 28.63 | ND | 11.08 | ND | ND |
| yibD | 20.37 | ND | 7.19 | ND | ND |
| yibE | ND | ND | very stable | ND | ND |
| yibF | very stable | ND | ND | ND | ND |
| yibG | 40.44 | 13.50 | 5.18 | ND | 6.22 |
| yicA | 21.24 | 8.77 | 6.72 | ND | ND |

| | | | | | |
|---|---|---|---|---|---|
| yicB | 9.57 | 10.71 | 5.10 | ND | ND |
| yicC | 11.08 | 9.80 | 4.78 | ND | ND |
| yicE | 11.40 | 8.47 | 4.10 | ND | 5.04 |
| yidA | 13.91 | ND | 5.48 | ND | 8.28 |
| yidB | ND | very stable | very stable | ND | ND |
| yidC | very stable | ND | very stable | ND | ND |
| yidE | very stable | very stable | very stable | ND | ND |
| yieF | ND | NA | NA | NA | NA |
| yieH | 17.85 | 14.73 | 10.82 | ND | ND |
| yifA | 21.42 | 12.51 | 5.72 | ND | ND |
| yigC | 20.94 | ND | 6.03 | ND | ND |
| yihA | NA | ND | 6.65 | ND | ND |
| yihB | NA | NA | 7.82 | ND | ND |
| yihD | NA | NA | ND | 5.02 | ND |
| yihF | 39.10 | ND | 6.81 | 9.47 | 6.28 |
| yiiB | 19.51 | ND | 9.07 | ND | ND |
| yiiD | 16.67 | very stable | NA | NA | NA |
| yiiE | 18.95 | 11.46 | 5.56 | ND | ND |
| yiiF | 13.74 | 17.08 | 6.06 | ND | ND |
| yiiG | very stable | ND | 6.01 | ND | 4.57 |
| yiiH | 27.76 | 23.65 | very stable | ND | ND |
| yiiI | 11.89 | 7.40 | 13.89 | ND | ND |
| yijB | 23.87 | ND | 19.51 | 9.64 | ND |
| yijC | 17.69 | ND | very stable | 12.38 | ND |
| yijD | 20.44 | ND | 14.67 | ND | ND |
| yijE | 16.06 | 10.98 | 5.80 | ND | ND |
| yijF | NA | NA | NA | NA | NA |
| yijG | ND | ND | NA | NA | NA |
| yijH | 14.20 | ND | 4.98 | 5.59 | 5.05 |
| yjaB | 23.38 | very stable | 15.52 | ND | ND |
| yjaD | 10.82 | 13.79 | 6.24 | ND | ND |
| yjaE | ND | very stable | very stable | ND | ND |
| yjaF | 28.97 | 15.71 | 9.49 | ND | 9.32 |
| yjaH | 25.75 | ND | 6.37 | ND | 11.03 |
| yjaI | 51.04 | very stable | 14.89 | ND | ND |
| yjaJ | very stable | very stable | 13.18 | ND | ND |
| yjbB | 13.55 | 7.76 | 5.60 | ND | ND |
| yjbC | 11.66 | 8.43 | 5.82 | ND | ND |
| yjbE | 13.08 | 14.75 | 6.78 | ND | ND |
| yjbF | 22.65 | 24.59 | 8.40 | ND | ND |
| yjcA | 12.50 | ND | very stable | 4.07 | ND |
| yjcD | 31.19 | 17.51 | 7.36 | ND | ND |
| yjcE | 11.79 | 7.10 | 5.44 | ND | ND |
| yjcF | 14.46 | 10.92 | 3.72 | ND | ND |
| yjdA | 26.92 | 16.86 | 5.68 | ND | ND |
| yjdB | 29.41 | 15.40 | 6.14 | ND | 6.51 |
| yjdE | very stable | very stable | ND | ND | ND |
| yjdI | NA | NA | NA | NA | NA |
| yjdJ | very stable | 21.50 | 14.21 | ND | ND |
| yjeA | ND | ND | very stable | ND | ND |

| | | | | | |
|---|---|---|---|---|---|
| yjeD | 62.66 | very stable | ND | ND | ND |
| yjeF | 17.42 | 21.35 | NA | NA | NA |
| yjeG | 35.53 | ND | 15.32 | ND | ND |
| yjfB | NA | NA | NA | NA | NA |
| yjfE | NA | NA | very stable | ND | ND |
| yjfG | 12.03 | 10.26 | 5.60 | 6.84 | 5.95 |
| yjfI | 29.92 | very stable | 9.68 | 12.03 | ND |
| yjfJ | 12.14 | 11.97 | 3.05 | ND | 5.03 |
| yjgB | 33.63 | 15.22 | 7.38 | ND | ND |
| yjgC | 16.22 | 13.09 | 3.07 | ND | ND |
| yjgD | 12.79 | 12.74 | 4.39 | ND | ND |
| yjgE | NA | NA | 4.62 | ND | ND |
| yjgF | 12.35 | ND | 4.73 | ND | ND |
| yjhA | 11.51 | ND | 4.53 | 6.04 | ND |
| yjhB | 31.39 | 15.15 | 10.64 | ND | ND |
| yjhC | 12.08 | 6.34 | 3.43 | ND | ND |
| yjhD | 8.47 | 5.99 | 3.70 | ND | ND |
| yjhE | NA | NA | 4.76 | 5.02 | 5.34 |
| yjhF | 9.98 | 12.36 | 4.91 | ND | ND |
| yjhH | NA | NA | 6.38 | 12.24 | ND |
| yjiB | 32.28 | ND | 7.04 | ND | ND |
| yjiE | 24.30 | ND | 9.17 | ND | ND |
| yjiF | NA | NA | 8.39 | ND | 11.82 |
| yjjA | 19.33 | ND | 7.51 | ND | ND |
| yjjB | very stable | very stable | 19.73 | ND | ND |
| yjjC | 20.69 | ND | 10.67 | ND | ND |
| yjjD | ND | very stable | ND | ND | ND |
| yjjE | 33.93 | ND | 25.73 | ND | ND |
| yjjF | 14.02 | 11.83 | 5.35 | ND | ND |
| yjjG | NA | NA | 3.44 | ND | ND |
| yjjH | 17.70 | 12.60 | 6.81 | ND | ND |
| ykaC | NA | NA | 4.98 | ND | ND |
| ykaE | 8.85 | 10.98 | NA | NA | NA |
| ykaF | 57.65 | 30.95 | very stable | ND | ND |
| ykbA | very stable | very stable | very stable | ND | ND |
| ykbB | NA | NA | 14.99 | ND | ND |
| ykbC | 41.41 | very stable | NA | NA | NA |
| ykbE | ND | ND | very stable | ND | ND |
| ykbF | very stable | ND | ND | ND | ND |
| ykcA | ND | ND | NA | NA | NA |
| ykcB | ND | very stable | very stable | ND | ND |
| ykcC | very stable | ND | very stable | ND | ND |
| ykcE | NA | NA | 5.52 | ND | ND |
| ykcF | 13.00 | 13.02 | 5.91 | ND | ND |
| ykcG | 20.94 | ND | 5.53 | ND | ND |
| ykdA | 29.79 | 23.66 | very stable | ND | ND |
| ykdB | very stable | very stable | 26.40 | ND | ND |
| ykhD | NA | NA | 3.17 | ND | ND |
| ykhE | 18.53 | ND | 6.26 | ND | ND |
| ykhF | 30.22 | 12.47 | 5.20 | ND | ND |

| | | | | | |
|---|---|---|---|---|---|
| ykhG | 49.14 | ND | very stable | ND | ND |
| ykhH | NA | NA | very stable | ND | ND |
| ykhI | very stable | ND | ND | ND | ND |
| ykhJ | NA | very stable | NA | NA | NA |
| ykhK | very stable | 17.26 | NA | NA | NA |
| ykiC | 14.58 | 12.11 | 4.52 | ND | ND |
| ykiD | 13.93 | 8.96 | 4.49 | ND | ND |
| ykiE | NA | NA | 4.60 | 4.39 | ND |
| ykiF | 16.45 | ND | 20.18 | ND | ND |
| ykiG | 11.88 | 14.77 | 4.45 | ND | 7.20 |
| ykiH | 17.42 | 7.43 | 3.53 | ND | ND |
| ykiI | NA | NA | 5.05 | ND | ND |
| ykjA | very stable | ND | very stable | ND | ND |
| ykjB | 15.18 | 8.88 | NA | NA | NA |
| ykjC | ND | ND | NA | NA | NA |
| ykjE | 10.70 | 8.22 | 3.66 | ND | ND |
| ykjF | 9.85 | 9.71 | 4.59 | ND | ND |
| ykjH | 12.60 | ND | 4.75 | ND | ND |
| ykjI | NA | NA | 6.80 | ND | ND |
| ykjJ | NA | ND | NA | NA | NA |
| ykjK | NA | NA | 6.05 | ND | ND |
| ylaC | 17.13 | 14.53 | very stable | ND | ND |
| ylaD | NA | NA | 5.99 | ND | ND |
| ylaE | 21.34 | 7.94 | 4.64 | ND | ND |
| ylaF | NA | NA | 3.73 | ND | ND |
| ylaG | NA | NA | 4.51 | ND | ND |
| ylbA | 15.92 | ND | 8.03 | ND | ND |
| ylbB | 16.07 | ND | 5.97 | ND | ND |
| ylbD | 13.00 | 6.51 | 3.11 | ND | 3.74 |
| ylbE | 16.57 | 21.38 | 6.76 | 7.27 | ND |
| ylcA | 55.78 | 17.67 | 5.18 | ND | 7.45 |
| ylcC | 14.99 | 11.36 | ND | ND | ND |
| ylcD | very stable | very stable | ND | ND | ND |
| ylcE | 22.24 | ND | very stable | ND | ND |
| ylcF | very stable | ND | very stable | ND | ND |
| ylcG | NA | NA | 2.97 | ND | ND |
| yldA | 49.76 | very stable | NA | NA | NA |
| yldB | NA | NA | NA | NA | NA |
| yldC | 20.78 | very stable | ND | ND | ND |
| yldE | 23.53 | ND | 8.99 | ND | ND |
| yleB | very stable | very stable | 13.34 | ND | ND |
| yleC | NA | NA | ND | ND | ND |
| yleD | NA | NA | ND | ND | ND |
| yleE | NA | NA | very stable | ND | ND |
| yleF | 18.91 | ND | 8.12 | ND | ND |
| ylfA | 61.08 | ND | 16.25 | ND | ND |
| ylfB | 24.98 | 19.73 | 23.00 | ND | ND |
| ylfC | 23.76 | 16.89 | 9.54 | ND | ND |
| ylfD | 15.56 | 11.45 | 6.64 | 6.54 | ND |
| ylfF | 19.33 | 11.43 | 4.98 | ND | ND |

| | | | | | |
|---|---|---|---|---|---|
| ylfH | 15.52 | 11.07 | 4.39 | ND | ND |
| ylfI | 23.35 | 13.25 | 11.17 | ND | ND |
| ylgB | NA | NA | NA | NA | NA |
| ylgC | 29.28 | 12.71 | 8.24 | ND | ND |
| ylgG | 13.85 | ND | 10.69 | 8.70 | ND |
| ylhB | 17.69 | 18.61 | 6.18 | ND | 9.00 |
| yliA | 49.68 | very stable | 10.63 | 10.54 | ND |
| yliB | NA | NA | very stable | ND | ND |
| yliC | very stable | 32.81 | very stable | ND | ND |
| yliD | very stable | very stable | ND | ND | ND |
| yliE | very stable | very stable | ND | ND | ND |
| yliF | NA | NA | NA | NA | NA |
| yliG | NA | NA | NA | NA | NA |
| yljB | ND | ND | very stable | ND | ND |
| yljC | NA | 20.22 | NA | NA | NA |
| yljD | ND | 19.71 | 6.88 | ND | ND |
| yljE | 16.15 | 8.69 | 4.69 | ND | ND |
| yljF | 15.21 | 14.71 | 5.34 | ND | ND |
| yljG | 12.89 | 16.47 | 4.08 | ND | ND |
| yljH | 22.83 | ND | ND | ND | ND |
| yljI | 13.55 | ND | 5.87 | 8.06 | ND |
| yljJ | 13.54 | ND | 5.81 | ND | ND |
| ylqL | 35.47 | 21.89 | 8.19 | ND | ND |
| ylxQ | 27.75 | 24.01 | 8.20 | ND | ND |
| ymaB | very stable | very stable | NA | NA | NA |
| ymbC | very stable | very stable | very stable | ND | ND |
| ymbD | very stable | ND | ND | ND | ND |
| ymbG | NA | NA | NA | NA | NA |
| ymbJ | very stable | very stable | ND | ND | ND |
| ymbK | ND | ND | ND | ND | ND |
| ymcA | very stable | 13.74 | very stable | ND | ND |
| ymcB | very stable | very stable | very stable | ND | ND |
| ymcC | very stable | ND | ND | ND | ND |
| ymcF | very stable | very stable | very stable | ND | ND |
| ymdC | 39.39 | ND | very stable | ND | ND |
| ymdE | NA | NA | 8.28 | 3.79 | ND |
| ymeA | NA | NA | NA | NA | NA |
| ymeB | 32.86 | very stable | ND | 9.62 | ND |
| ymfD | 23.55 | 14.26 | 11.76 | ND | ND |
| ymfE | 48.67 | very stable | very stable | ND | ND |
| ymgB | NA | NA | NA | NA | NA |
| ymgC | 32.31 | very stable | ND | ND | ND |
| ymgF | ND | very stable | NA | NA | NA |
| ymgG | 50.19 | very stable | very stable | ND | ND |
| ymgH | 17.53 | ND | NA | NA | NA |
| ymgI | 14.29 | ND | ND | ND | ND |
| ymgJ | 11.68 | ND | ND | ND | ND |
| ymgK | 14.42 | 16.68 | 8.21 | ND | ND |
| ymhA | NA | NA | ND | ND | ND |
| ymhC | 23.58 | 20.12 | NA | NA | NA |

| | | | | | |
|---|---|---|---|---|---|
| ymhG | 30.21 | 15.49 | 25.45 | ND | ND |
| ymiA | 10.54 | 7.73 | 4.79 | ND | ND |
| ymjE | 20.07 | ND | 12.53 | ND | ND |
| ymjF | 16.82 | 13.02 | 6.47 | ND | ND |
| ynaA | very stable | 22.59 | very stable | ND | ND |
| ynaB | 22.67 | 13.42 | 9.02 | 6.38 | ND |
| ynaC | 14.70 | 20.60 | 4.92 | ND | ND |
| ynaD | 15.34 | 13.09 | 4.16 | ND | ND |
| ynaE | 16.63 | 17.32 | 8.35 | ND | ND |
| ynaG | 17.88 | ND | NA | NA | NA |
| ynaH | NA | NA | ND | ND | ND |
| ynbA | 17.88 | 20.56 | ND | ND | ND |
| ynbB | 28.94 | 21.18 | 18.54 | 12.68 | 14.61 |
| ynbC | 16.77 | 16.09 | 10.58 | 8.64 | ND |
| ynbD | 19.19 | 23.77 | 9.89 | 10.26 | ND |
| ynbE | 16.34 | 16.50 | 7.17 | 7.81 | 8.68 |
| yncA | 9.49 | 6.57 | 3.57 | ND | 4.37 |
| yncB | 13.92 | 9.97 | 5.42 | ND | 7.76 |
| yndA | 21.74 | 10.66 | ND | ND | ND |
| yndB | 13.57 | 7.76 | 5.67 | 6.55 | ND |
| yndC | 21.87 | 18.98 | very stable | ND | ND |
| yndD | 14.30 | ND | 6.83 | ND | ND |
| yndE | ND | very stable | ND | ND | ND |
| yndF | 24.84 | 16.93 | ND | ND | ND |
| yndG | very stable | ND | ND | ND | ND |
| yneB | 24.09 | 18.29 | 10.16 | ND | ND |
| yneC | 18.77 | 12.72 | ND | ND | 9.73 |
| yneD | NA | NA | NA | NA | NA |
| yneE | very stable | ND | very stable | ND | ND |
| yneF | very stable | ND | 6.36 | ND | ND |
| yneG | very stable | 23.51 | very stable | ND | ND |
| yneH | ND | ND | ND | ND | ND |
| ynfC | 10.56 | 10.01 | 3.70 | ND | ND |
| ynfD | 19.83 | 31.75 | ND | ND | ND |
| ynfG | 36.04 | 13.34 | very stable | ND | ND |
| ynfH | ND | very stable | very stable | ND | ND |
| yngA | 14.16 | 11.37 | NA | NA | NA |
| yngB | 11.85 | 5.49 | 4.17 | ND | ND |
| yngE | 11.58 | 8.99 | 6.03 | ND | ND |
| yngF | 17.15 | ND | 6.83 | 7.48 | ND |
| yngG | NA | NA | 9.15 | 6.47 | ND |
| ynhA | 18.74 | 8.68 | 2.76 | ND | ND |
| ynhC | 17.81 | ND | 6.46 | 6.18 | ND |
| ynhD | 17.10 | 14.96 | 8.34 | ND | ND |
| ynhH | 16.32 | 9.05 | 6.93 | ND | ND |
| ynhI | 18.91 | 11.51 | 5.76 | ND | ND |
| yniC | 23.54 | ND | 6.37 | ND | ND |
| yniG | ND | 36.93 | ND | ND | ND |
| yniH | 12.27 | 6.87 | 4.35 | ND | 5.81 |
| yniI | NA | 14.66 | ND | ND | ND |

| | | | | | |
|---|---|---|---|---|---|
| yniJ | 28.09 | ND | 7.91 | ND | ND |
| ynjB | NA | NA | ND | ND | ND |
| ynjC | very stable | very stable | 21.52 | ND | ND |
| ynjD | ND | very stable | very stable | ND | ND |
| ynjE | ND | ND | very stable | ND | ND |
| ynjF | ND | ND | ND | ND | ND |
| ynjG | 46.50 | very stable | ND | ND | ND |
| ynjH | 21.76 | ND | 9.91 | ND | ND |
| ynjI | 11.93 | 19.64 | NA | NA | NA |
| ynjJ | 18.93 | 25.47 | 13.77 | ND | ND |
| yoaB | NA | NA | 4.98 | ND | ND |
| yoaD | 26.71 | 17.25 | 9.17 | ND | ND |
| yoaF | NA | NA | very stable | ND | ND |
| yoaG | 15.97 | ND | 8.60 | ND | ND |
| yoaH | 19.45 | 20.14 | 6.24 | ND | ND |
| yoaI | 20.83 | 14.23 | ND | ND | ND |
| yobA | 8.86 | 10.54 | 2.85 | ND | ND |
| yobC | very stable | very stable | NA | NA | NA |
| yofM | 26.85 | 9.40 | 6.58 | ND | ND |
| yogE | 26.18 | ND | 5.37 | ND | ND |
| yogG | 22.37 | 11.55 | 4.83 | ND | ND |
| yogI | 16.25 | ND | 7.45 | ND | ND |
| yogJ | 14.41 | 11.08 | 6.09 | ND | ND |
| yogL | very stable | very stable | ND | ND | ND |
| yogM | 42.61 | 27.67 | 20.42 | ND | ND |
| yohC | 12.25 | 9.64 | ND | ND | ND |
| yohD | very stable | very stable | ND | ND | ND |
| yohH | 18.13 | ND | NA | NA | NA |
| yohJ | 26.05 | ND | ND | ND | ND |
| yoiA | NA | NA | ND | ND | ND |
| yoiB | very stable | very stable | very stable | ND | ND |
| yoiC | very stable | very stable | NA | NA | NA |
| yojB | 20.94 | ND | 6.06 | ND | ND |
| yojC | very stable | ND | NA | NA | NA |
| ypaA | 16.18 | ND | 10.98 | ND | ND |
| ypaC | 14.23 | 14.04 | 5.14 | ND | ND |
| ypaD | NA | NA | NA | NA | NA |
| ypaG | NA | ND | ND | ND | ND |
| ypaH | 11.13 | 6.60 | 3.85 | ND | ND |
| ypaI | 32.68 | ND | ND | ND | ND |
| ypbB | NA | very stable | NA | NA | NA |
| ypbC | 9.58 | 13.68 | 8.87 | 6.67 | ND |
| ypbD | 8.59 | 8.82 | 4.81 | 4.03 | ND |
| ypbG | NA | NA | very stable | ND | ND |
| ypcA | 19.95 | very stable | very stable | ND | ND |
| ypcB | 23.62 | very stable | very stable | ND | ND |
| ypcC | 19.42 | very stable | very stable | ND | ND |
| ypcD | 18.43 | ND | ND | ND | ND |
| ypcG | 17.04 | ND | ND | ND | ND |
| ypcH | 12.20 | ND | ND | 8.96 | ND |

| | | | | | |
|---|---|---|---|---|---|
| ypdA | 9.71 | 22.96 | 6.10 | ND | ND |
| ypdB | 9.08 | 6.63 | 4.46 | ND | ND |
| ypdC | 9.47 | 6.73 | 4.05 | ND | ND |
| ypdD | 9.53 | 6.97 | 3.63 | 4.46 | 4.50 |
| ypfD | 10.22 | 5.74 | 5.66 | ND | ND |
| ypfE | 41.10 | ND | ND | ND | ND |
| ypfF | 18.42 | 11.03 | 6.03 | 8.90 | ND |
| ypgB | 63.20 | ND | 17.18 | ND | ND |
| ypgC | 23.52 | 23.20 | 6.80 | ND | 8.43 |
| ypgD | 25.70 | ND | 5.64 | 9.47 | ND |
| ypgH | NA | NA | 7.32 | ND | ND |
| yphA | 9.95 | 11.06 | 5.01 | ND | ND |
| yphC | ND | ND | ND | 7.48 | ND |
| yphH | very stable | very stable | very stable | ND | ND |
| yphI | ND | ND | NA | NA | NA |
| yphJ | 30.75 | ND | NA | NA | NA |
| yphK | ND | 22.35 | very stable | ND | ND |
| yphL | 26.32 | ND | ND | 9.81 | ND |
| ypiA | very stable | very stable | very stable | ND | ND |
| ypiB | 75.70 | very stable | very stable | ND | ND |
| ypiE | very stable | very stable | 11.70 | ND | ND |
| ypiH | very stable | very stable | NA | NA | NA |
| ypiJ | ND | ND | ND | ND | ND |
| ypiK | NA | very stable | NA | NA | NA |
| ypiL | ND | ND | ND | ND | ND |
| ypjA | very stable | ND | 28.20 | 11.24 | ND |
| ypjB | 22.71 | 12.22 | ND | ND | ND |
| ypjC | 27.17 | 17.95 | 8.45 | ND | ND |
| ypjF | 17.24 | 15.65 | 7.16 | ND | ND |
| ypjG | NA | NA | 5.24 | 7.15 | ND |
| ypjH | 15.13 | ND | 8.94 | 7.34 | ND |
| ypjI | 17.48 | 10.50 | 5.88 | ND | ND |
| yqaB | 17.36 | ND | 6.51 | ND | ND |
| yqaC | 14.44 | 11.78 | 4.82 | ND | ND |
| yqaD | 19.15 | 14.31 | 7.36 | ND | ND |
| yqaG | 6.77 | 7.59 | 2.89 | ND | ND |
| yqbA | 16.84 | 13.08 | NA | NA | NA |
| yqbF | very stable | very stable | ND | ND | ND |
| yqbH | NA | very stable | 5.53 | ND | ND |
| yqbI | 27.57 | ND | NA | NA | NA |
| yqbJ | very stable | NA | NA | NA | NA |
| yqbK | NA | NA | 9.14 | ND | ND |
| yqcA | NA | NA | very stable | ND | ND |
| yqcB | 41.39 | ND | very stable | ND | ND |
| yqcC | very stable | very stable | ND | ND | ND |
| yqcD | ND | very stable | ND | ND | ND |
| yqcE | ND | 23.26 | very stable | ND | ND |
| yqcF | 15.37 | 13.84 | 9.01 | 10.11 | ND |
| yqcG | 35.96 | 18.01 | 8.64 | ND | 8.57 |
| yqdA | 25.18 | very stable | ND | ND | ND |

| | | | | | |
|---|---|---|---|---|---|
| yqeA | 14.30 | 11.35 | 4.23 | ND | ND |
| yqeB | 20.65 | very stable | 5.65 | ND | ND |
| yqeC | NA | NA | 21.67 | ND | ND |
| yqeD | 16.25 | 7.90 | 5.27 | ND | ND |
| yqeH | 24.26 | ND | NA | NA | NA |
| yqeI | NA | NA | very stable | ND | ND |
| yqeL | 16.26 | 12.09 | 5.25 | ND | ND |
| yqfA | 25.79 | 11.17 | 5.64 | ND | ND |
| yqfC | 24.26 | 13.91 | ND | ND | ND |
| yqfD | NA | NA | NA | NA | NA |
| yqfE | 14.95 | 21.53 | 5.76 | ND | ND |
| yqfF | 12.44 | 16.97 | 9.06 | ND | ND |
| yqfG | ND | very stable | ND | ND | ND |
| yqgA | 10.81 | 8.44 | 3.36 | ND | ND |
| yqgC | 10.12 | 7.13 | 4.09 | ND | 5.41 |
| yqgE | 15.99 | 17.16 | 7.90 | ND | ND |
| yqgF | ND | ND | NA | NA | NA |
| yqgG | 17.60 | ND | 8.31 | ND | ND |
| yqhA | NA | NA | NA | NA | NA |
| yqiA | NA | 11.35 | 5.83 | ND | ND |
| yqjA | NA | NA | NA | NA | NA |
| yqjB | 13.00 | 5.93 | 4.62 | ND | ND |
| yqjD | 18.57 | 18.25 | 8.53 | 8.84 | ND |
| yqjE | 12.26 | 12.06 | 5.71 | ND | ND |
| yraA | very stable | very stable | very stable | ND | ND |
| yraB | 19.34 | 25.54 | 4.14 | ND | ND |
| yraC | 14.99 | ND | 5.43 | 6.45 | ND |
| yraD | 20.11 | very stable | NA | NA | NA |
| yraE | 17.05 | 12.75 | NA | NA | NA |
| yraF | 12.47 | 7.73 | NA | NA | NA |
| yrbA | 14.77 | 8.03 | NA | NA | NA |
| yrbB | 59.97 | 19.58 | NA | NA | NA |
| yrbC | 56.22 | 16.45 | 12.70 | ND | ND |
| yrbD | ND | ND | very stable | ND | ND |
| yrbE | ND | very stable | NA | NA | NA |
| yrbF | ND | ND | ND | ND | ND |
| yrbG | NA | NA | very stable | ND | ND |
| yrbH | ND | very stable | ND | ND | ND |
| yrbI | 20.50 | 12.70 | 7.40 | ND | ND |
| yrcA | 12.33 | 9.92 | 4.74 | ND | ND |
| yrcB | very stable | very stable | very stable | ND | ND |
| yrdB | NA | NA | 5.03 | ND | ND |
| yreA | NA | NA | NA | NA | NA |
| yreB | 19.99 | 32.35 | NA | NA | NA |
| yreC | NA | NA | NA | NA | NA |
| yreD | 17.68 | 11.12 | NA | NA | NA |
| yreE | 18.43 | 12.32 | NA | NA | NA |
| yrfA | 17.96 | 10.02 | 6.18 | ND | ND |
| yrfB | 11.06 | ND | 13.39 | ND | ND |
| yrfC | 22.70 | ND | ND | ND | ND |

| | | | | | |
|---|---|---|---|---|---|
| yrfD | 19.23 | 15.59 | 7.75 | ND | ND |
| yrfE | 13.74 | 7.57 | 4.62 | ND | ND |
| yrgA | 13.03 | 7.98 | 5.25 | ND | ND |
| yrgE | 15.12 | very stable | NA | NA | NA |
| yrgF | 14.54 | ND | 8.28 | 10.96 | 9.16 |
| yrgG | 14.23 | 17.74 | 6.67 | ND | ND |
| yrgH | 9.88 | ND | 5.61 | 5.76 | ND |
| yrgI | 10.80 | ND | 6.08 | 6.26 | ND |
| yrhH | 21.83 | 11.58 | NA | NA | NA |
| yriA | NA | NA | 4.66 | ND | ND |
| yriB | 12.45 | 9.49 | NA | NA | NA |
| yriC | 10.15 | 7.24 | 2.92 | ND | ND |
| yriD | NA | NA | 4.32 | 8.45 | 5.18 |
| yrjA | 9.34 | 8.20 | 4.01 | 4.75 | 4.55 |
| yrjB | 10.93 | ND | 4.35 | ND | ND |
| yrjC | 13.21 | very stable | 5.14 | ND | ND |
| yrjD | 19.54 | very stable | 5.76 | ND | ND |
| yrjE | 40.63 | very stable | 10.48 | 9.73 | ND |
| yrjF | 18.78 | very stable | 10.10 | 11.49 | ND |
| yrjG | 8.25 | 5.19 | 3.14 | ND | 5.04 |
| yrjI | 15.97 | ND | 5.39 | ND | ND |
| ysaA | 16.24 | 7.60 | 5.68 | ND | ND |
| ysaB | 14.59 | 16.13 | 7.30 | ND | ND |
| ysaC | ND | ND | 10.25 | 7.90 | ND |
| ysaD | 13.24 | 19.56 | 6.21 | 7.97 | ND |
| ysbA | 10.70 | 21.77 | 4.54 | ND | 5.19 |
| ysbB | 12.16 | very stable | 5.10 | ND | 6.78 |
| ysbC | 13.70 | very stable | 6.64 | ND | ND |
| ysbD | 17.59 | 11.76 | 5.77 | ND | ND |
| yscA | 15.50 | 11.42 | 6.52 | 8.14 | 5.59 |
| yscB | 35.46 | 48.89 | 9.90 | ND | 7.29 |
| yscD | 23.65 | 17.98 | ND | ND | ND |
| yscE | 15.37 | 11.26 | 4.97 | ND | ND |
| ysdA | 8.88 | 12.21 | 3.19 | ND | ND |
| ysdB | very stable | 35.14 | 23.43 | ND | ND |
| ysdC | ND | very stable | ND | ND | ND |
| ysdE | 27.28 | 8.95 | 4.87 | ND | ND |
| yseA | 7.60 | 7.78 | 4.92 | ND | ND |
| yseB | NA | NA | NA | NA | NA |
| yseC | NA | NA | NA | NA | NA |
| yseD | 17.61 | ND | NA | NA | NA |
| yseE | NA | very stable | ND | ND | ND |
| yseF | 15.33 | very stable | 5.71 | ND | ND |
| yseH | 15.25 | ND | 9.07 | ND | 7.59 |
| yseI | NA | NA | 5.62 | 7.82 | 5.33 |
| ysfB | 13.40 | 9.66 | 5.11 | ND | ND |
| ysfC | 10.76 | 9.34 | 4.00 | 6.23 | 5.43 |
| ysfD | 12.27 | 7.55 | 3.43 | ND | ND |
| ysfG | ND | ND | 6.42 | ND | ND |
| ysgA | 24.75 | NA | NA | NA | NA |

| | | | | | |
|---|---|---|---|---|---|
| ysgB | 26.87 | 24.61 | 12.84 | ND | ND |
| ysgC | NA | NA | 3.74 | 7.57 | 4.13 |
| yshA | 41.52 | very stable | 15.31 | ND | ND |
| yshB | 12.29 | ND | 5.11 | 6.33 | 8.33 |
| ysiA | 11.22 | 12.63 | 4.86 | 6.44 | 5.75 |
| ysiB | 12.24 | 13.65 | 4.82 | ND | 6.34 |
| ysiC | 18.94 | 9.33 | 7.47 | ND | ND |
| ysiD | 17.16 | ND | 10.41 | ND | 14.17 |
| ysiE | 13.30 | 7.65 | 4.20 | ND | ND |
| ysiG | 29.50 | ND | ND | ND | ND |
| ysjA | 30.22 | very stable | 8.86 | ND | ND |
| ysjB | NA | NA | NA | NA | NA |
| ysjC | 11.51 | 10.03 | 3.65 | ND | 4.89 |
| ysjD | very stable | very stable | 16.67 | ND | ND |
| ysjE | 12.06 | 8.84 | 2.79 | ND | ND |
| ysjF | 11.87 | 9.94 | 4.42 | 7.69 | ND |
| ysjG | 15.30 | ND | 4.47 | 7.75 | ND |
| ysjH | very stable | ND | 13.45 | ND | ND |
| ysxL | very stable | very stable | very stable | ND | ND |
| ytaA | 17.27 | 19.07 | 5.20 | 4.42 | ND |
| ytaB | 29.81 | ND | 6.89 | 9.88 | ND |
| ytaD | 21.36 | 13.71 | 6.44 | ND | ND |
| ytbA | NA | NA | NA | NA | NA |
| ytbB | 11.57 | 6.30 | 4.46 | ND | ND |
| ytbC | 16.37 | 20.70 | 7.53 | 6.27 | ND |
| ytbD | 41.43 | 16.91 | 7.76 | ND | ND |
| ytbE | ND | very stable | 10.97 | 9.62 | ND |
| ytcA | 15.24 | ND | 5.22 | 5.61 | 7.37 |
| ytcB | 11.20 | ND | 4.46 | 6.31 | 5.11 |
| ytcC | 17.31 | 19.02 | 4.76 | ND | ND |
| ytcD | 25.31 | very stable | 8.37 | 13.65 | ND |
| ytcE | 26.46 | ND | 9.27 | ND | ND |
| ytdA | 35.35 | 17.05 | 20.69 | ND | ND |
| ytdB | very stable | very stable | ND | 7.69 | ND |
| ytdC | 8.82 | 10.10 | 4.18 | ND | ND |
| ytdD | NA | NA | NA | NA | NA |
| ytdF | 12.18 | 9.39 | 4.73 | 4.38 | 4.88 |
| yteA | 8.87 | 6.42 | 3.71 | ND | 4.31 |
| yteB | 19.56 | ND | 7.09 | ND | ND |
| yteC | 42.14 | very stable | ND | ND | ND |
| yteD | NA | ND | very stable | ND | ND |
| yteE | NA | very stable | ND | ND | ND |
| yteG | 33.51 | very stable | 8.53 | 8.54 | ND |
| ytfA | 19.49 | ND | ND | ND | ND |
| ytfB | 25.51 | ND | 10.76 | 8.81 | ND |
| ytgA | 35.07 | 11.43 | 11.56 | 6.87 | ND |
| ytgB | 19.60 | 12.30 | 9.42 | 9.94 | ND |
| ytgC | very stable | very stable | 27.85 | ND | ND |
| ytgD | 20.82 | ND | very stable | ND | ND |
| ytgE | 12.76 | 7.86 | 6.11 | ND | ND |

| | | | | | |
|---|---|---|---|---|---|
| ytgF | 16.93 | 8.74 | 6.42 | 7.75 | ND |
| ytgG | 19.51 | 27.97 | 10.51 | 6.24 | ND |
| ytgH | 25.33 | 13.81 | 11.77 | 5.65 | ND |
| ythA | very stable | very stable | 16.43 | 7.79 | ND |
| ythB | very stable | very stable | 8.53 | ND | ND |
| ythC | 20.78 | ND | 8.80 | 5.84 | ND |
| ytiA | 26.36 | 18.93 | 12.58 | ND | ND |
| ytjA | 21.91 | ND | ND | 5.85 | ND |
| ytjD | 22.75 | ND | ND | ND | ND |
| ytjE | 14.94 | 22.10 | 4.26 | ND | ND |
| ytjF | ND | ND | very stable | ND | ND |
| ytjG | ND | ND | NA | NA | NA |
| ytjH | 32.28 | 15.07 | ND | ND | ND |
| yuaA | 13.31 | 13.66 | 7.26 | ND | ND |
| yuaB | 8.95 | 8.60 | 4.15 | ND | 4.56 |
| yuaC | 15.56 | 16.19 | 7.78 | ND | ND |
| yuaD | 12.09 | 7.65 | 4.68 | ND | ND |
| yuaE | 25.46 | 12.91 | 8.39 | 5.75 | 5.57 |
| yucF | 15.51 | 9.11 | 6.21 | 7.69 | 6.00 |
| yucG | 14.54 | ND | 8.77 | ND | ND |
| yudA | 26.96 | 23.08 | 7.40 | ND | ND |
| yudB | 8.63 | 8.73 | 3.36 | ND | 6.54 |
| yudC | NA | NA | NA | NA | NA |
| yudD | 17.38 | 8.62 | 4.05 | ND | ND |
| yudE | 19.02 | 9.46 | NA | NA | NA |
| yudF | 34.55 | 7.99 | NA | NA | NA |
| yudG | 13.02 | 8.78 | NA | NA | NA |
| yudH | NA | NA | 10.08 | ND | ND |
| yudI | 30.77 | 16.77 | 10.65 | ND | ND |
| yudJ | 39.49 | 21.84 | 6.46 | 7.48 | 6.79 |
| yudK | 21.74 | ND | 11.98 | 9.46 | ND |
| yudL | 15.17 | ND | 5.69 | 8.33 | 8.16 |
| yueA | 14.05 | 14.31 | 4.20 | ND | 7.22 |
| yueB | NA | 17.06 | NA | NA | NA |
| yueC | ND | very stable | very stable | ND | ND |
| yueD | 13.83 | 6.92 | 4.25 | 5.64 | 3.87 |
| yueE | very stable | 36.58 | 5.19 | ND | ND |
| yueF | 42.33 | 10.97 | 7.67 | ND | ND |
| yufA | 17.49 | 10.24 | ND | ND | ND |
| yufC | 27.75 | 6.43 | 4.93 | ND | ND |
| yugA | 18.91 | 9.81 | 7.51 | ND | ND |
| yugB | very stable | ND | very stable | ND | ND |
| yugC | 8.76 | 9.42 | 4.58 | ND | ND |
| yugD | 11.05 | 8.48 | 5.37 | ND | ND |
| yuhA | 18.24 | ND | NA | NA | NA |
| yuhB | 19.10 | 9.96 | 5.92 | ND | ND |
| yuhC | 42.02 | 28.21 | very stable | ND | ND |
| yuhD | 13.17 | 18.18 | 6.81 | ND | ND |
| yuhE | 25.30 | 26.59 | 13.35 | ND | ND |
| yuhH | 15.05 | 10.84 | 5.90 | ND | ND |

| | | | | | |
|---|---|---|---|---|---|
| yuhI | 13.73 | 6.68 | 4.63 | ND | ND |
| yuhJ | 15.49 | 9.24 | NA | NA | NA |
| yuiA | ND | ND | NA | NA | NA |
| yuiB | 9.81 | 7.17 | 3.49 | ND | ND |
| yuiC | 24.27 | ND | ND | ND | ND |
| yuiD | 12.13 | 8.52 | ND | ND | ND |
| yuiE | 33.81 | 20.25 | 11.19 | ND | ND |
| yujA | ND | ND | NA | NA | NA |
| yujB | 19.76 | ND | very stable | ND | ND |
| yujC | NA | NA | very stable | ND | ND |
| yujD | 42.47 | very stable | NA | NA | NA |
| yujE | 76.27 | very stable | ND | ND | ND |
| yujF | very stable | very stable | NA | NA | NA |
| yujG | 49.81 | 18.83 | very stable | ND | ND |
| yvaA | very stable | 9.04 | ND | ND | ND |
| yvaB | 14.87 | 8.88 | 5.31 | ND | ND |
| yvaD | 56.74 | very stable | ND | ND | ND |
| yvcA | 10.12 | 8.31 | 7.96 | ND | 6.37 |
| yvcC | very stable | very stable | very stable | ND | ND |
| yvdA | NA | NA | 4.65 | ND | ND |
| yvdB | ND | 19.22 | 9.41 | ND | ND |
| yvdC | 14.18 | 8.85 | 4.47 | ND | ND |
| yvdD | 31.88 | 9.16 | 4.53 | 10.24 | 5.08 |
| yvdE | 16.80 | ND | 6.74 | 6.42 | 7.43 |
| yvdF | 11.68 | 19.55 | 6.90 | 6.00 | ND |
| yvdG | 30.10 | ND | 14.15 | ND | ND |
| yveA | NA | NA | very stable | ND | ND |
| yveB | very stable | very stable | ND | ND | ND |
| yveC | ND | ND | 14.15 | ND | ND |
| yveD | 11.89 | very stable | 5.37 | ND | ND |
| yveE | 30.14 | 13.45 | 27.22 | ND | ND |
| yveF | 17.60 | 9.05 | 5.12 | ND | ND |
| yveG | 21.56 | ND | 5.89 | 10.32 | ND |
| yveH | ND | ND | very stable | ND | ND |
| yveI | 19.11 | 12.19 | 6.31 | ND | ND |
| yvfA | very stable | ND | NA | NA | NA |
| yvfB | NA | NA | NA | NA | NA |
| yvgA | NA | NA | 6.32 | ND | ND |
| yvhA | 11.64 | 12.09 | 4.86 | ND | 6.59 |
| yvhB | 14.51 | 14.34 | 3.85 | 4.73 | 4.43 |
| yviA | 11.92 | 9.71 | 4.82 | ND | ND |
| yviB | NA | NA | 4.18 | ND | ND |
| yviC | 15.03 | 10.75 | 5.83 | ND | ND |
| yviH | ND | ND | 10.81 | ND | ND |
| yviI | very stable | very stable | NA | NA | NA |
| yviJ | 15.96 | 16.59 | 4.84 | ND | 6.18 |
| yvjA | very stable | very stable | very stable | ND | ND |
| yvjB | 15.00 | 10.73 | 6.19 | 7.65 | ND |
| ywaB | 33.40 | 17.09 | 10.60 | ND | ND |
| ywaC | very stable | ND | ND | ND | ND |

| | | | | | |
|---|---|---|---|---|---|
| ywaD | 14.70 | ND | 11.89 | ND | ND |
| ywaE | 23.88 | 11.76 | 5.04 | 7.69 | 8.97 |
| ywaF | 11.54 | 6.56 | 4.17 | 5.88 | ND |
| ywaG | 11.74 | 9.53 | 4.48 | ND | 5.21 |
| ywaH | ND | 28.87 | very stable | ND | ND |
| ywaI | 12.64 | 10.57 | 5.12 | 5.47 | 8.22 |
| ywbA | 15.69 | 10.34 | 4.95 | ND | 5.28 |
| ywbB | 65.81 | very stable | 13.50 | ND | ND |
| ywcC | 15.46 | 19.93 | 3.06 | 5.35 | 4.23 |
| ywdA | very stable | very stable | ND | ND | ND |
| ywdB | very stable | ND | 8.41 | ND | ND |
| ywdC | 32.13 | ND | ND | ND | ND |
| ywdD | very stable | ND | very stable | ND | ND |
| ywdE | 41.03 | 12.26 | very stable | ND | ND |
| ywdF | 11.90 | 9.88 | 4.27 | ND | 5.08 |
| ywdG | NA | NA | 4.84 | ND | ND |
| yweA | 15.46 | 14.03 | 5.66 | 7.00 | ND |
| yweB | 19.38 | 18.58 | 13.90 | ND | ND |
| yweC | 15.61 | 8.75 | 4.74 | ND | ND |
| yweD | 15.34 | 16.39 | 9.98 | ND | ND |
| yweE | 20.83 | 20.60 | 3.92 | ND | 4.13 |
| yweF | very stable | 24.10 | 8.78 | ND | ND |
| ywfA | 18.07 | very stable | 6.07 | 8.84 | ND |
| ywfB | NA | NA | very stable | ND | ND |
| ywfC | 18.83 | ND | 9.27 | ND | ND |
| ywfD | 12.54 | 14.17 | 5.13 | 5.95 | ND |
| ywfE | 11.81 | ND | 5.19 | 5.19 | ND |
| ywfF | 11.39 | 6.77 | 4.39 | 5.43 | ND |
| ywfG | NA | NA | 5.93 | 5.26 | ND |
| ywfH | 12.96 | 6.10 | 4.04 | 4.23 | ND |
| ywgA | 15.79 | 11.18 | 5.68 | 7.22 | ND |
| ywhA | ND | very stable | very stable | ND | ND |
| ywhB | NA | NA | 5.42 | 6.53 | ND |
| ywiA | 12.67 | 9.23 | 6.38 | ND | ND |
| ywiB | 16.29 | 8.42 | 4.95 | ND | ND |
| ywiC | ND | very stable | NA | NA | NA |
| ywiD | 33.03 | 17.49 | 7.10 | ND | ND |
| ywiE | 22.48 | 8.31 | very stable | ND | ND |
| ywiG | NA | NA | 22.01 | ND | ND |
| ywiH | very stable | ND | very stable | ND | ND |
| ywiI | NA | NA | very stable | ND | ND |
| ywjA | 31.82 | ND | 10.37 | 10.95 | 10.97 |
| ywjB | 10.69 | 12.74 | 8.06 | ND | ND |
| ywjC | 26.19 | 20.36 | NA | NA | NA |
| ywjD | 11.53 | 6.50 | 3.59 | 5.26 | ND |
| ywjE | 27.37 | 10.82 | 4.34 | 4.54 | ND |
| ywjF | NA | NA | 4.93 | 6.27 | ND |
| ywjG | 31.19 | 14.51 | 7.54 | ND | 8.68 |
| ywjH | NA | NA | NA | NA | NA |
| yxaB | 12.10 | 5.07 | NA | NA | NA |

| | | | | | |
|---|---|---|---|---|---|
| yxaC | 28.16 | 12.49 | 3.98 | ND | 4.56 |
| yxaF | 26.52 | ND | 8.21 | ND | ND |
| yxbA | 9.76 | 9.38 | 4.30 | ND | ND |
| yxbC | 23.81 | 8.53 | 4.97 | 5.68 | ND |
| yxbD | ND | very stable | ND | ND | ND |
| yxbE | 23.22 | NA | NA | NA | NA |
| yxbF | NA | NA | NA | NA | NA |
| yxcA | 37.75 | 7.71 | 5.53 | 8.13 | ND |
| yxcB | 16.33 | 5.35 | 3.20 | ND | ND |
| yxcD | 23.19 | 8.41 | 4.94 | 5.61 | 4.94 |
| yxcE | NA | NA | NA | NA | NA |
| yxdB | 27.25 | 7.75 | 4.22 | ND | ND |
| yxdC | 20.57 | 24.09 | 5.94 | ND | ND |
| yxdD | 19.62 | 12.10 | 4.34 | ND | ND |
| yxdE | very stable | ND | 10.24 | ND | ND |
| yxdF | 29.12 | very stable | ND | ND | ND |
| yxdG | 31.93 | ND | very stable | ND | ND |
| yxeA | 22.10 | ND | 7.95 | ND | ND |
| yxeB | very stable | 26.47 | 10.95 | ND | ND |
| yxfA | 20.30 | 9.78 | 7.09 | ND | ND |
| yxfB | very stable | 22.79 | 10.14 | ND | ND |
| yxfC | 18.49 | 9.67 | 6.21 | ND | 6.20 |
| yyaL | 14.12 | 7.14 | 3.64 | ND | ND |
| zitP | very stable | ND | ND | ND | ND |
| zitQ | 46.21 | very stable | 11.30 | ND | ND |
| zitR | 20.27 | 19.87 | 5.98 | ND | 10.33 |
| zitS | NA | NA | 7.13 | ND | ND |
| zwf | 10.54 | 12.73 | 4.95 | ND | ND |
