# Supplementary material for: Role of mRNA Stability during Bacterial Adaptation
Source: PLoS One. 2013 Mar 13;8(3):e59059. doi: 10.1371/journal.pone.0059059 (PMC3596320; doi:10.1371/journal.pone.0059059)
Supplement: Table S2 — Composition of the growth medium. (PDF) [file pone.0059059.s003.pdf]

Table S2: Composition of the culture medium

The lines separating the different products indicated those that are dissolved together. Values into brackets in front of KH_2PO_4 and K_2HPO_4 indicate the required concentrations to obtain a buffered media for pre-cultures.

Product	Concentration (g/L)	Product	Concentration (g/L)
Glucose	20	Isoleucine	0.025
Sodium acetate	1	Leucine	0.05875
Ammonium citrate	0.6	Valine	0.04125
KH_2PO_4	3 (9)	Cysteine	0.17
K_2HPO_4	2,5 (7,5)	Phenylalanine	0.28
$MgCl_2$, $6H_2O$	0.2	Tyrosine	0.29
$FeSO_4$, $7H_2O$	0.011	Adenine	0.05
$CaCl_2$, $2H_2O$	0.05	Guanine	0.05
$ZnSO_4$, $7H_2O$	0.005	Uracil	0.05
$COCl_2$, $6H_2O$	0.0025	Xanthin	0.05
Alanine	0.24	P-aminobenzoic acid	0.01
Arginine	0.12	Biotin	0.01
Asparagine	0.34	Cyano-cobalamine (B12)	0.001
Glutamine	0.51	Folic acid	0.01
Glycine	0.17	Inosine	0.025
Histidine	0.11	Nicotinic acid	0.001
Lysine	0.35	Orotic acid	0.005
Methionine	0.12	Ca panthotenate	0.001
Proline	0.68	Pyridoxamine	0.005
Serine	0.34	Pyridoxin (B6)	0.002
Threonine	0.23	Riboflavin (B2)	0.001
Tryptophan	0.05	Thiamine	0.001
		D,L-6,8-thioctic acid	0.0025
		Thymidine	0.025

Table II. Macrokinetic parameters of *L. lactis* continuous cultures at different growth rates.

D (h^{-1})	0.09	0.24	0.35	0.47
[X] (g/L)	0.76 +/- 0.02	0.79 +/- 0.01	0.75 +/- 0.01	0.75 +/- 0.01
[isoleucine] (µM)	<10	<10	<10	<10
[glucose] (mM)	43.23 +/- 4	70.9 +/- 2	81.05 +/- 5	84.41 +/- 2
[lactate] (mM)	116.52 +/- 6	77.57 +/- 4	57.12 +/- 6	51.85 +/- 2
$Y_{Gluc, Lact}$ (mol Lact/mol Gluc)	1.7	1.8	1.7	1.6
qGluc (mmol/gX/h)	8.28 +/- 1	12.99 +/- 1	15.81 +/- 2	20.25 +/- 1
νLact (mmol/gX/h)	13.33 +/- 1	22.47 +/-1	25.42 +/- 3	31.54 +/-1

D = dilution rate, [ ] = residual concentration, X = biomass, $Y_{Gluc, Lact}$ = lactate yield from glucose, qGluc = specific glucose consumption rate, νLact = specific lactate production rate.

Table III: Overlapping of the different transcriptional responses with different selection criteria on datasets.

Compared conditions	Overlapping percentages					
	p-values <0.05			ratios <1 or >1		
	ratios <1 or >1	ratios <0.8 or >1.2	ratios <0.67 or >1.5	p-values<0.05	p-values<0.03	p-values<0.02
µ and CS	29%	29%	31%	29%	25%	21%
µ and IS	24%	23%	20%	24%	22%	22%
µ and SR	10%	9%	5%	10%	11%	9%
SR and µ	16%	15%	8%	16%	18%	14%
SR and CS	15%	15%	17%	15%	14%	11%
SR and IS	68%	70%	72%	68%	65%	59%

Values in percentage represent the fraction of common genes compared to the whole growth rate response (for the three first lines) or the whole stringent response (for the three last lines). Abbreviations: SR = stringent response regulon, CS = carbon starvation response, IS = isoleucine starvation response, µ = growth rate response

Figures legends

Figure 1: Biomass production yields from amino acids in *L. lactis* IL1403 grown in continuous culture at different growth rates. Color code: = aspartic acid, = glutamic acid, = asparagine, = serine, = glutamine, = valine, = leucine, = isoleucine. Amino acids with positive yield are consumed by the bacteria whereas those with negative yields are produced.

Figure 2: Average expression profiles of genes classified in the different clusters following transcriptomic analysis of *L. lactis* IL1403 continuous culture at different growth rate. X axis: compared growth rate; Y axis: average expression ratios. Error bars represent the 5% confidence interval.

Figure 3: Distribution of genes from clusters C and D along *Lactococcus lactis* IL1403 chromosome. Dark bars indicate the global repartition of genes along the chromosome and the straight lines represent the normal repartition. Light grey and white bars are respectively representatives of the chromosomal localization of genes belonging to clusters C and D. Two division of the chromosome have been assessed: in 4 (upper graph) and 15 (lower graph) parts.

Figure 4: Venn diagrams comparing the growth rate transcriptomic response with other stress responses in *L. lactis* IL1403. Growth rate (μ) is compared with stringent response (SR) (a), isoleucine starvation (IS) (b), and carbon starvation (CS) (c). + and – respectively represents over- or under- expressed genes during IS, CS and SR. For μ stimulon the variation sense of the genes previously selected (see Materials and methods) has been inverted. Missing genes of the different set of analysis have been removed. Numbers of genes in the various domains are specified on the graph.

Figure 5: Venn diagrams comparing the stringent transcriptomic response with other stress responses in *L. lactis* IL1403. Stringent response (SR) regulon is compared with isoleucine starvation (IS) and carbon starvation (CS) responses. + and – respectively represents over- and under- expressed genes in response to IS, CS or SR. Numbers of genes in the various domains are specified on the graph.

Figure 6. Ornithine biosynthesis pathways in *L. lactis*.
Ornithine can be synthesised either from glutamate or from arginine trough the arginine deiminase pathway (ADI pathway). Thick arrows indicate up regulation of the corresponding genes upon growth rate decrease (down regulation upon growth rate increase).

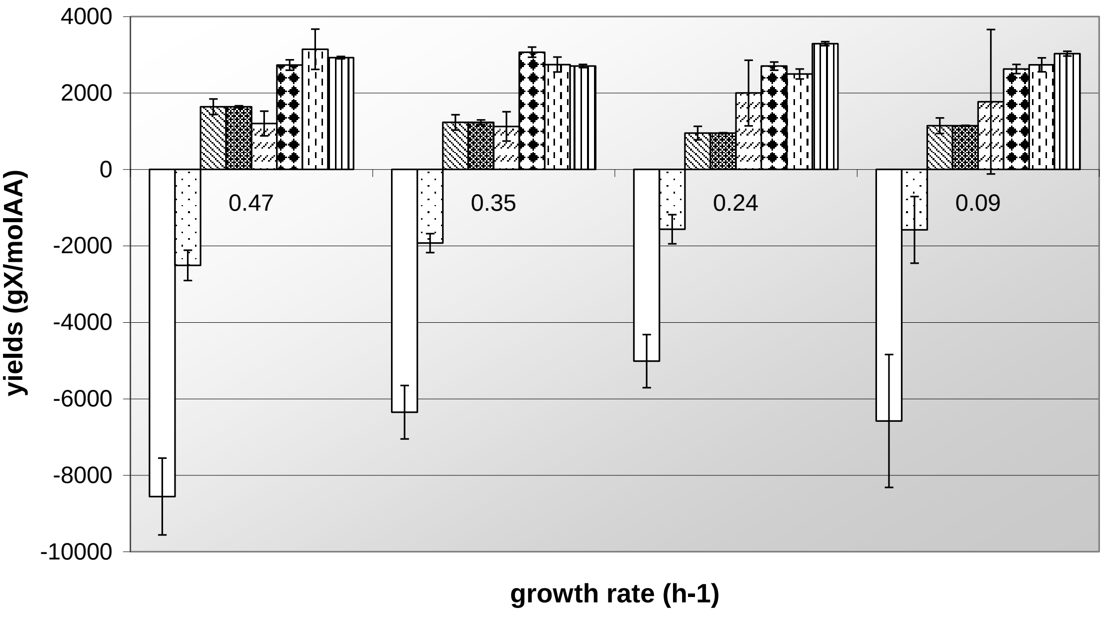
4000
2000
0
-2000
-4000
-6000
-8000
-10000
yields (gX/molAA)
0.47
0.35
0.24
0.09
growth rate (h-1)

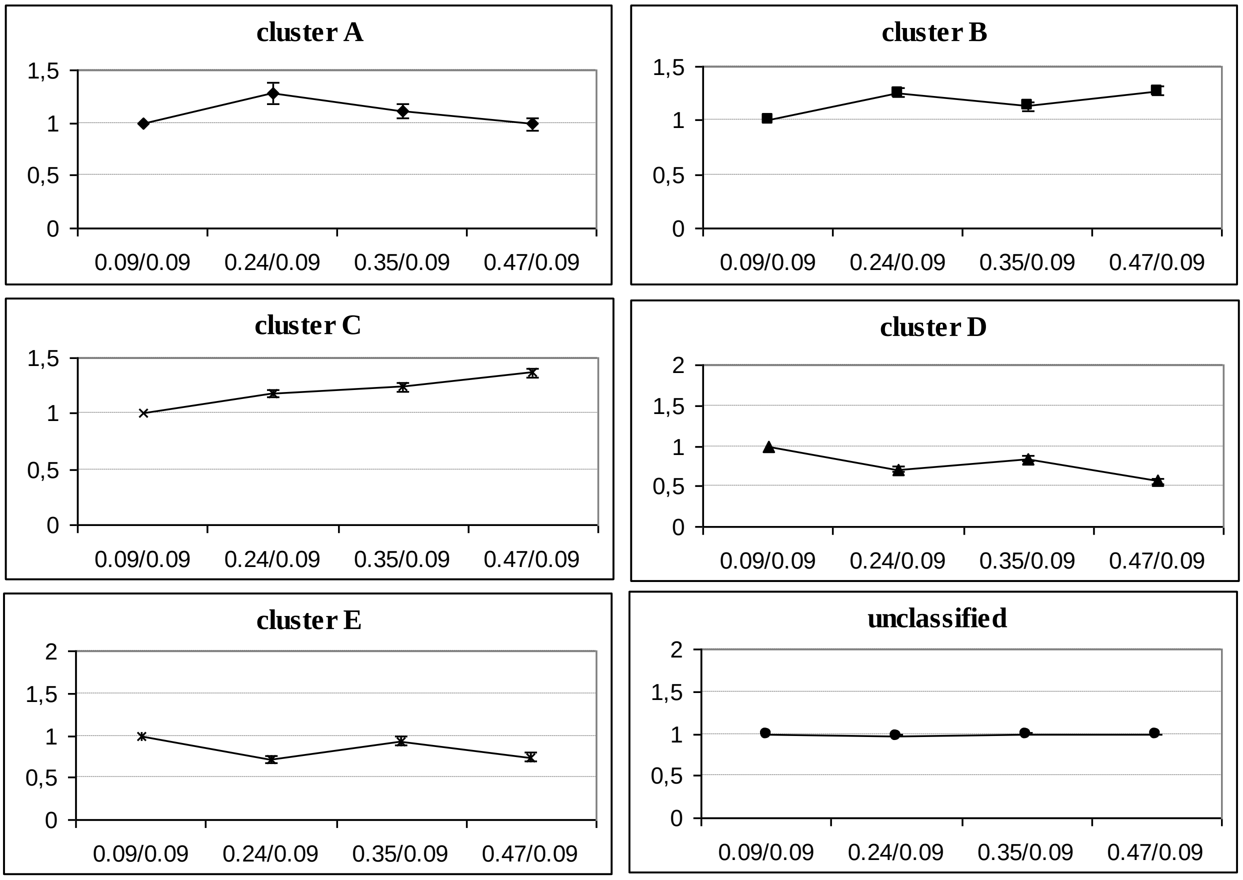
cluster A
1,5
1
0,5
0
0.09/0.09
0.24/0.09
0.35/0.09
0.47/0.09
cluster B
cluster C
cluster D
2
cluster E
unclassified

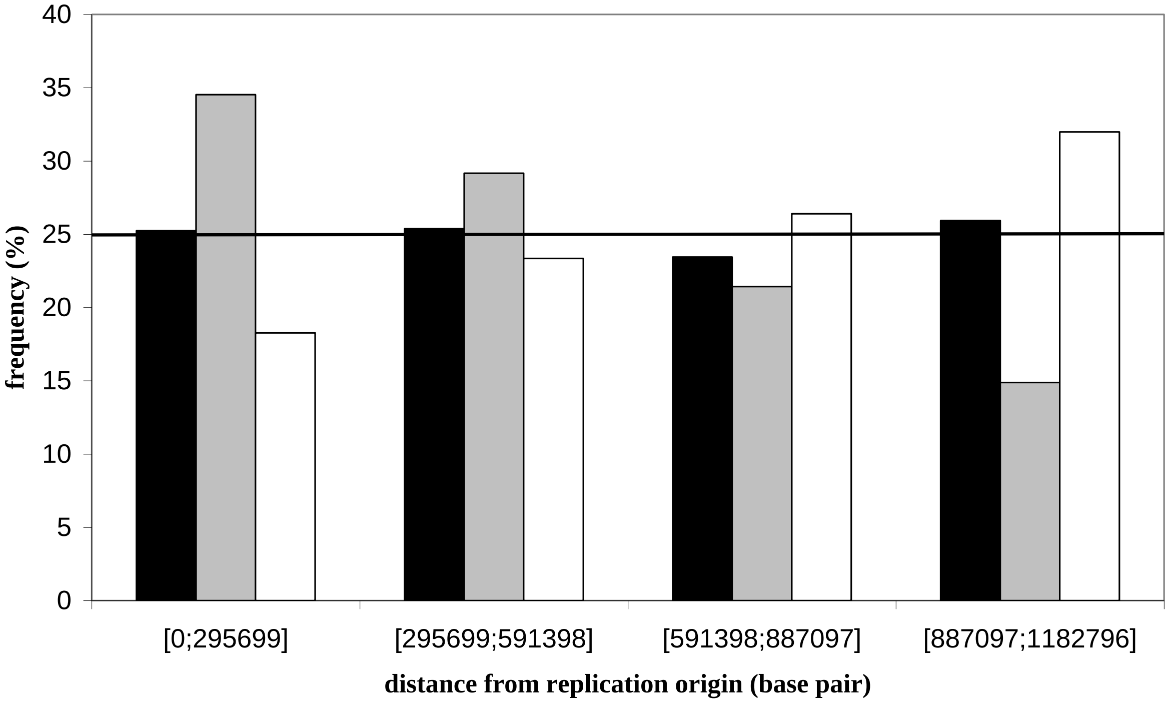

40
35
30
25
20
15
10
5
0
frequency (%)
[0;295699]
[295699;591398]
[591398;887097]
[887097;1182796]
distance from replication origin (base pair)

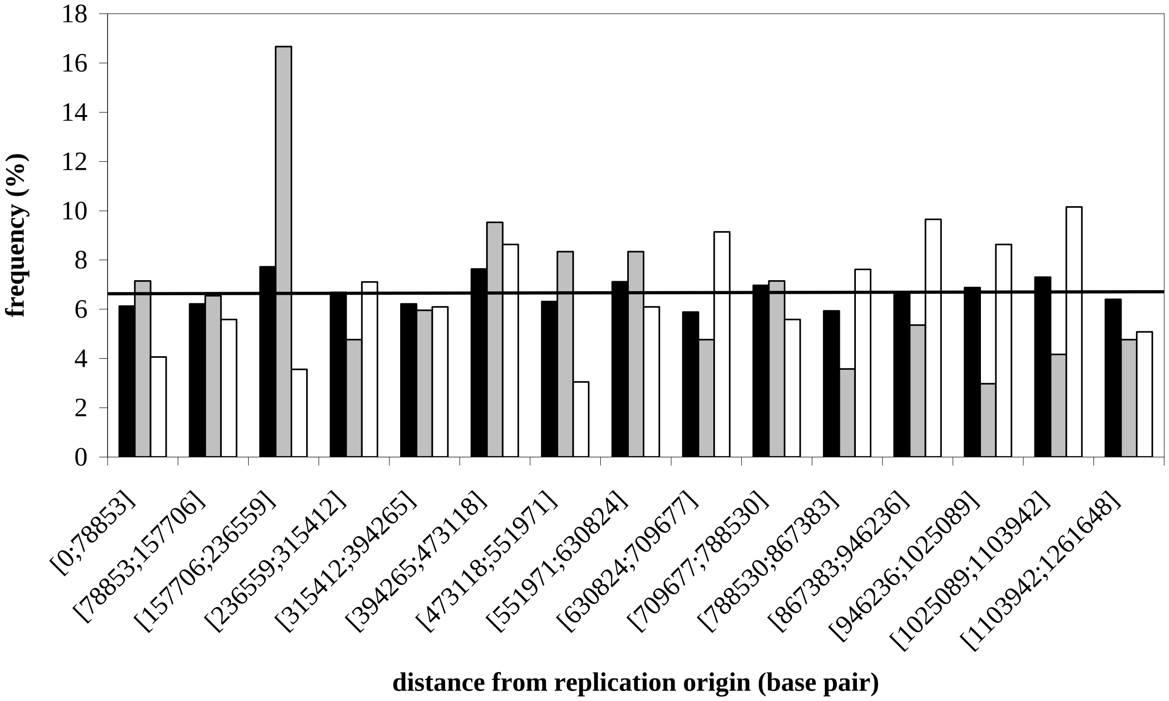

18
16
14
12
10
8
6
4
2
0
frequency (%)
[0;78853]
[78853;157706]
[157706;236559]
[236559;315412]
[315412;394265]
[394265;473118]
[473118;551971]
[551971;630824]
[630824;709677]
[709677;788530]
[788530;867383]
[867383;946236]
[946236;1025089]
[1025089;1103942]
[1103942;1261648]
distance from replication origin (base pair)

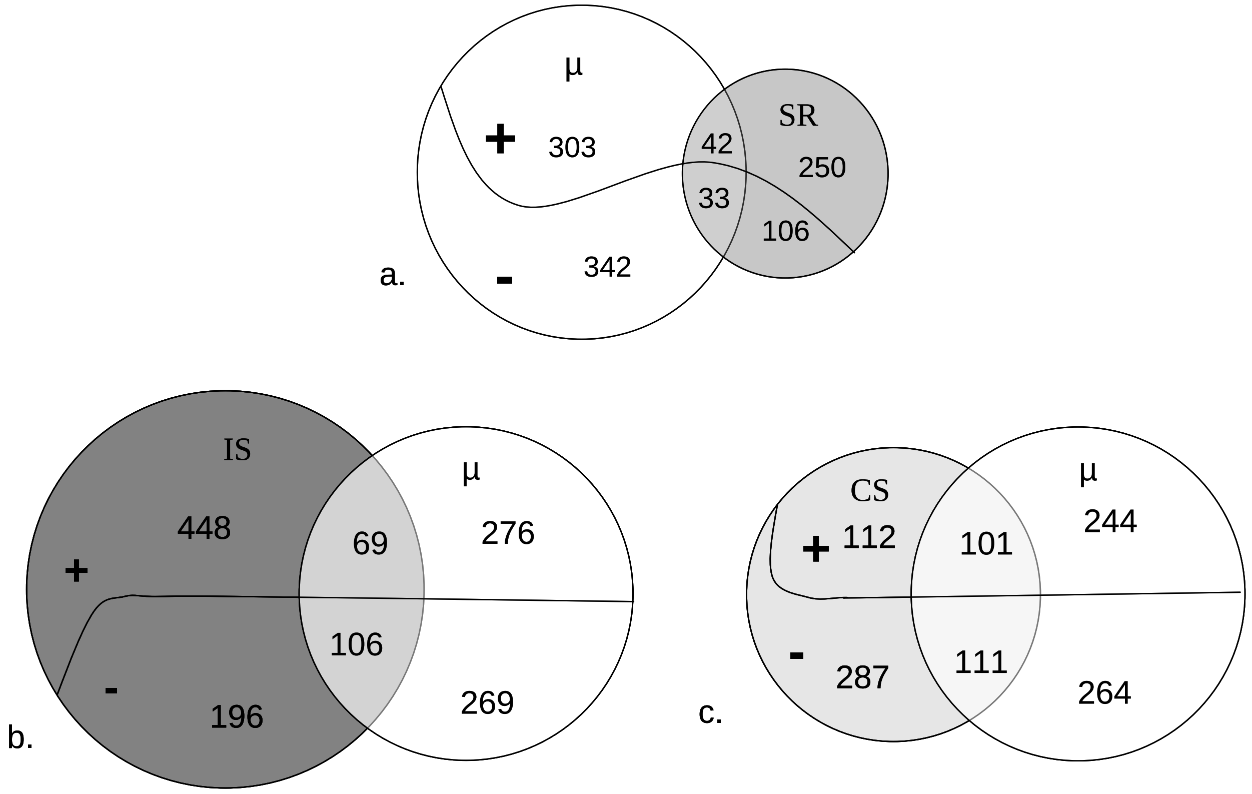

μ
SR
+
303
42
250
33
106
a.
-
342
IS
μ
448
69
276
+
106
-
269
196
b.
CS
μ
112
101
244
+
-
111
287
264
c.

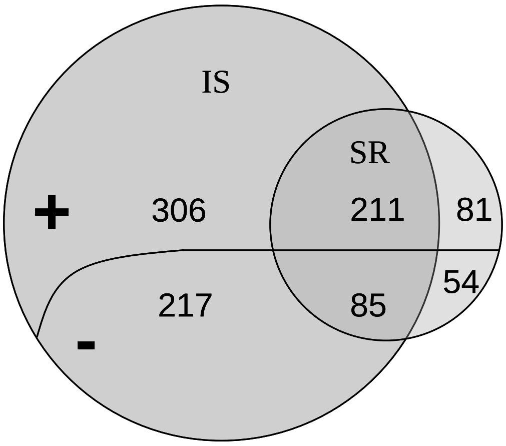

a.

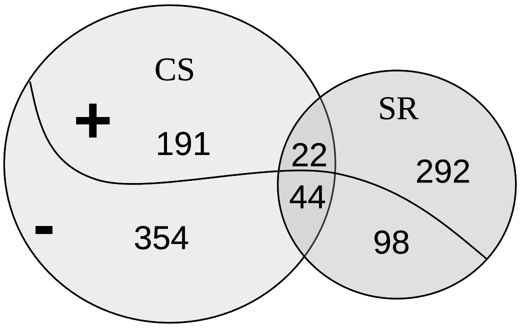

b.

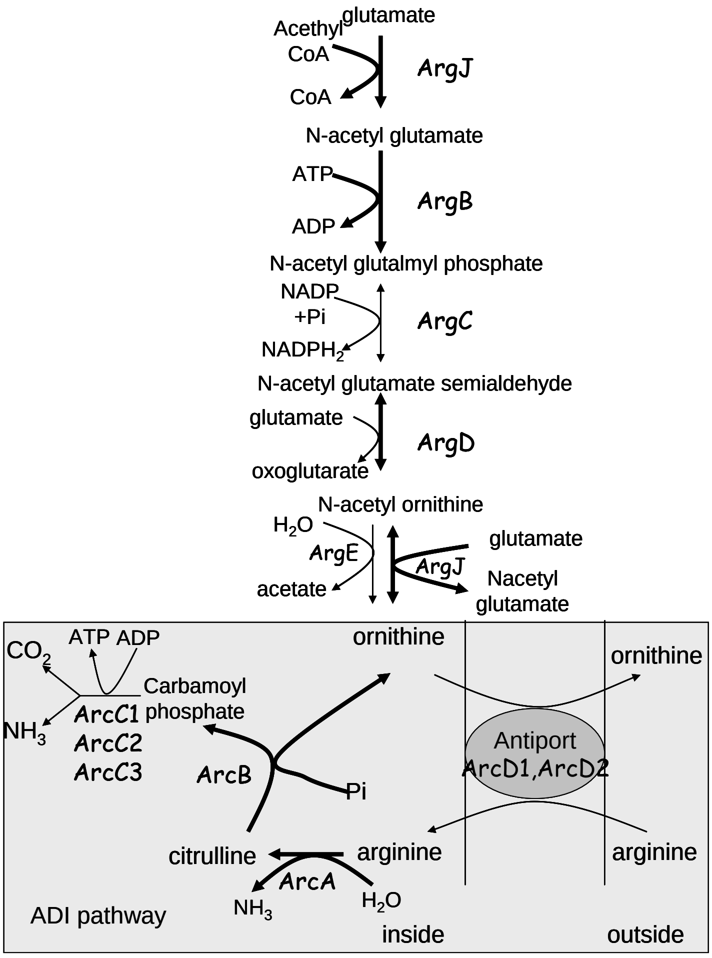

glutamate
Acethyl CoA
CoA
ArgJ
N-acetyl glutamate
ATP
ADP
ArgB
N-acetyl glutalmyl phosphate
NADP +Pi
NADPH2
ArgC
N-acetyl glutamate semialdehyde
glutamate
oxoglutarate
ArgD
N-acetyl ornithine
H2O
ArgE
acetate
glutamate
ArgJ
Nacetyl glutamate
CO2
ATP ADP
Carbamoyl phosphate
NH3
ArcC1
ArcC2
ArcC3
ornithine
ornithine
Antiport
ArcD1,ArcD2
ArcB
Pi
citrulline
arginine
arginine
ArcA
NH3
H2O
ADI pathway
inside
outside
